# The barriers and facilitators influencing the sustainability of hospital-based interventions: a systematic review

**DOI:** 10.1186/s12913-020-05434-9

**Published:** 2020-06-28

**Authors:** Julie Cowie, Avril Nicoll, Elena D. Dimova, Pauline Campbell, Edward A. Duncan

**Affiliations:** 1 Nursing, Midwifery and Allied Health Professions Research Unit (NMAHP RU), Glasgow Caledonian University, Govan Mbeki Building, Cowcaddens Road, Glasgow, G4 0BX Scotland; 2 Health Services Research Unit, University of Aberdeen, 2nd Floor, Health Sciences Building, Foresterhill, Aberdeen, AB25 2ZD Scotland; 3 Department of Nursing and Health, School of Life Sciences, Glasgow Caledonian University, Govan Mbeki Building, Cowcaddens Road, Glasgow, G4 0BX Scotland; 4 Nursing, Midwifery and Allied Health Professions Research Unit (NMAHP RU), Unit 13 Scion House, University of Stirling Innovation Park, Stirling, FK9 4NF Scotland

**Keywords:** Barriers, Dynamic, Facilitators, Hospital-based interventions, Implementation, Sustainability, Systematic review

## Abstract

**Background:**

Identifying factors that influence sustained implementation of hospital-based interventions is key to ensuring evidence-based best practice is maintained across the NHS. This study aimed to identify, appraise and synthesise the barriers and facilitators that influenced the delivery of sustained healthcare interventions in a hospital-based setting.

**Methods:**

A systematic review reported in accordance with PRISMA. Eight electronic databases were reviewed in addition to a hand search of Implementation Science journal and reference lists of included articles. Two reviewers were used to screen potential abstracts and full text papers against a selection criteria. Study quality was also independently assessed by two reviewers. Barriers and facilitators were extracted and mapped to a consolidated sustainability framework.

**Results:**

Our searching identified 154,757 records. We screened 14,626 abstracts and retrieved 431 full text papers, of which 32 studies met the selection criteria. The majority of studies employed a qualitative design (23/32) and were conducted in the UK (8/32) and the USA (8/32). Interventions or programmes were all multicomponent, with the majority aimed at improving the quality of patient care and/ or safety (22/32). Sustainability was inconsistently reported across 30 studies. Barriers and facilitators were reported in all studies. The key facilitators included a clear accountability of roles and responsibilities (23/32); ensuring the availability of strong leadership and champions advocating the use of the intervention (22/32), and provision of adequate support available at an organisational level (21/32). The most frequently reported barrier to sustainability was inadequate staff resourcing (15/32). Our review also identified the importance of inwards spread and development of the initiative over time, as well as the unpredictability of sustainability and the need for multifaceted approaches.

**Conclusions:**

This review has important implications for practice and research as it increases understanding of the factors that faciliate and hinder intervention sustainability. It also highlights the need for more consistent and complete reporting of sustainability to ensure that lessons learned can be of direct benefit to future implementation of interventions.

**Trial registration:**

The review is registered on PROSPERO (CRD42017081992).

## Background

Hospitals are challenging and complex environments that have been the focus for a series of implementation projects in recent years [1]. However, even when successfully implemented, interventions frequently stop being delivered after the initial funding has ceased [2]. Despite calls in the literature for guidance on sustaining interventions [3] such research remains sporadic [4]. This lack of guidance means that the NHS may make significant investment in evidence based interventions only to find that their delivery drifts and/or ceases over time to the detriment of patients. Sustaining effective interventions in practice is essential to improve health outcomes, reduce research waste, and build practitioners’ confidence in the value of adopting new interventions. However, methodological issues including a lack of agreed terminology and access to long-term data continue to hamper research in this field [5]. A universal definition of sustainablity is still lacking. Different studies have described it as maintenance, continued use, institutionalised, routine use, durability and achieving stability [4, 6, 7]. In this review, we are guided by the recent work of Moore and colleagues (2017) [8] which defines five key constructs to help define sustainability (as discussed later in the paper). At a rudimentary level, we define sustainability as being the enduring implementation of an intervention after its initial roll-out in practice.

Understanding factors that lead to sustained implementation in hospital settings is therefore of considerable research and practice benefit. Structured approaches using theories, models and frameworks to identify factors that influence *implementation* outcomes can provide an understanding of why implementation can succeed or fail [9]. Multiple systematic reviews have been conducted to identify such influencers [1, 10, 11]. Geerligs et al. [1], for example, included 43 papers investigating staff experience of implementation of patient-focused interventions in hospitals. They extracted barriers and facilitators and organised them into 12 categories making three key and dynamically interacting domains for implementation: the system, staff, and intervention. However, less attention has been given to promoting intervention *sustainability* after initial roll-out in practice, and most studies have focused on community and public health settings rather than hospitals [12]. Agreed sustainability research priorities include testing frameworks for their empirical utility, and understanding the relationship between sustainability and context [5]; as with the process of implementation, the inherently dynamic nature of sustainability [13] makes this work important but methodologically challenging.

Collectively, previous studies have identified the need to explore the application of sustainability frameworks, and address the gap in knowledge relating to intervention sustainability in hospitals [12]. Sustainability frameworks are structures that seek to define factors that influence implementation outcomes. They are useful in providing a theoretical underpinning to sustaining interventions such that success or failure of an intervention can be explained and better strategies for future studies can be adopted. Although theoretical frameworks have been used to understand some aspects of implementation, there has been less attention given to issues of intervention sustainability [13, 14].

In this paper, we present our findings from a systematic review of empirical studies, where theoretical frameworks were used to address sustainability of hospital-based interventions.

## Methods

### Study design

We conducted a systematic review using well established Cochrane methodology [15] to identify the barriers and facilitators that influence the delivery of sustained healthcare interventions in a hospital-based setting. This review followed the decisions and procedures that were prespecified in advance, and published in detail in our study protocol [16, 17]. Data was reported using the Preferred Reporting Items for Systematic review and Meta-Analysis (PRISMA) statement [18] (see Additional file 15) and the protocol developed using the PRISMA protocol checklist (PRISMA-P) [19]. The review is registered on PROSPERO (CRD42017081992).

### Information sources and search strategy

We employed a four-step approach to the development of the search strategies including the identification of search strategies from previous reviews of sustainability [3, 4, 6, 7, 20–23]; team consensus on which terms to use as part of the search strategy; identification of relevant search strategies published in high quality peer-reviewed systematic reviews; combining of key terms and different MEdical Subject Headings (MESH) and piloting and refining the search using MEDLINE (Ovid) database before adapting the search strategy for use in other databases. Further details are reported in Cowie et al. (2018) [17]**.**

We combined a series of free-text terms and MEdical Subject Headings (MESH) for: (a) framework (eg, frameworks, theories, models), (b) sustainability (eg, durability, long-term implementation) and (c) hospital (eg, ward, patient). Boolean operators and wild-cards were used to account for plurals and variations in spelling. The search strategy was peer-reviewed by an academic librarian in accordance with PRESS guidelines [24]. The search string used for MEDLINE (Ovid) is shown in Additional file 1.

#### Electronic searches

Eight electronic databases were systematically searched from January 2008 to December 2017: MEDLINE (Ovid), AMED (Ovid), CINAHL (EBSCO), Embase (Ovid) and Cochrane Library (e.g. CENTRAL, CDSR, DARE, HTA). We applied a date restriction in line with the development of Medical Research Council’s (MRC) revised complex intervention framework published in 2008 [25]. This framework provides a comprehensive structure for the development and testing of any complex interventions, and it is likely that the most relevant studies to our review would have been conducted following the framework’s development. It is also likely that interventions developed using the framework are theoretically sound and clearly defined thus allowing us to better understand and extrapolate how the frameworks are used in practice.

#### Other searches

We did not conduct any supplementary searches of grey literature due to resource and time constraints. However, we hand searched Implementation Science as we noted in our preliminary scoping work that a number of relevant papers had been published in this journal. Reference lists of all included articles were also searched.

### Eligibility criteria

Our predefined selection criteria are summarised in Additional file 2. We included peer-reviewed empirical studies published in English which reported using some form of theoretical framework to address the sustainability of hospital-based interventions. We defined a hospital-based intervention as any intervention that is delivered within a hospital environment, is aimed at improving patient care, and that directly involves care delivery to patients or staff, but not including ambulatory care, virtual or lab-based interventions. Non-research study designs (e.g. unstructured reviews or overviews, theoretical papers, commentaries or opinion papers, protocol, case study, editorial, audit, letter) were excluded.

In the case of studies performed across multiple settings, studies were excluded where results pertaining to the hospital setting were not clearly identifiable. In addition, if the service provided was regarded as an out-patient clinic, then the study was also excluded. Studies that did not discuss a specific intervention or programme (i.e. solely reported programmes at a general systems level) or only discussed sustainability (enduring use of an intervention after initial roll-out) prospectively (i.e. an empirical study had not been carried out) were excluded. Similarly, studies were excluded where sustainability was not a specific concern of the study (i.e. it was concerned only with adoption and initial implementation of the intervention / programme) or where no reference was made to theories, frameworks or models related to sustainability .

### Selection of studies

Study records were imported from the different databases into an Endnote file. Records that were published before 2008 were removed, and remaining records were de-deduplicated using a method recommended by Bramer et al. (2016) [26]. One reviewer screened all titles (PC) removing any clearly irrelevant papers. Two pairs of reviewers then independently screened any potential abstracts (JC, PC, AN, EDD). The abstracts were independently ranked as relevant, irrelevant or unsure. Studies ranked as irrelevant by both reviewers were excluded. We obtained the full papers for the remaining studies; two reviewers (JC, PC, AN, EDD) then independently assessed these against the selection criteria (Additional file 2). Disagreements were resolved initially through discussion, followed by a third independent reviewer as required. All of the review authors are highly experienced systematic reviewers.

### Data extraction

We used a standardised pre-piloted form based on the TIDieR reporting guidelines which were selected as they allowed us to profile the intervention (and those delivering the intervention) in significant detail using the following headings: why, what, how, where, when and how much, tailoring, modifications and fidelity [27]. We also extracted details about the study population, participant demographics, study design and methods used; study setting and other relevant contextual information; intervention / programme aims, theoretical frameworks (including justification for the use of the framework), and details of the intervention / programme, and comparison conditions.

Data was also extracted for any evidence of sustained change (e.g. length of time that the intervention was delivered, any associations reported by the authors about intervention and sustained effectiveness), which outcomes were measured and a brief summary of key findings.

Data identified as a barrier or facilitator to the sustainability of hospital-based interventions was extracted (author, year, country, direct quotes, page numbers) verbatim and coded by one reviewer (EDD or AN), and independently checked by a second review author (PC, JC). Any ambiguity identified was resolved through discussion with other members of the review team. We define a facilitator as any factor that contributes to the sustainability of an intervention beyond the implementation period. We define a barrier as any factor that obstructs the sustained delivery of an intervention. These definitions are in line with those proposed by Bach-Mortensen et al. (2018) [28].

### Data coding

#### Theories, models and frameworks

The terms theory, model and framework are used widely and often interchangeably. We therefore took a pragmatic decision to refer to ‘frameworks’, but used the taxonomy of theories, models and frameworks developed by Nilsen 2015 [29] to help define what theory/model/framework was employed. In addition, we drew on the typology described by Bradbury-Jones et al. 2014 [30] to assess the level of visibility of the framework used. This allowed us to better understand the role and level of influence of frameworks in trying to sustain interventions. The typology proposed by Bradbury-Jones et al. (2014) [30] defines a range of theoretical visibility which can be applied to studies to asses the level of theory evident in qualitative research. Use of theory can be defined across 5 categories ranging from highly visible and used throughout to an apparent absence of theory. The typology is defined further in Table 1.

**Table 1:** Table of included studies

Study 1. First author 2. Year ^(ref)^ 3. Design 4. Country	Aim 1. Aim 2. Focus	Study population and setting 1. Participants 2. Setting	Framework 1. Name 2. Category of implementation theory, model and framework 3. Theoretical visibility
1. Ament 2. 2017 [31] 3. QS 4. Netherlands	1. To explore key factors of the sustainability of two multidisciplinary hospital-based surgical care programs (ERAS and SSP). 2. Sustainability	1. MDT members (*n* = 26) incl. Surgeons, NP and nurses, 14 hospitals; 10/14 for ERAS, 4/14 for SSP 2. Surgical care	1. CFIR 2. Determinant framework 3. Level 5
1. Belizan 2. 2011 [32] 3. QS 4. South Africa	1. To understand the processes involved in initiating and implementing an audit programme, as well as factors contributing to the sustainability of the programme. 2. Implementation	1. Clinicians, regional and provincial coordinators, and other experienced stakeholders (*n* = 48) 2. Public hospitals	1. Stage-of-change conceptual framework 2. Classic theory 3. Level 5
1. Bergh [33] 2. 2014 3. MMS 4. South Africa	1. To systematically evaluate implementation status of facility-based kangaroo mother care services in four African countries 2. Non-sustainability	1. Key stakeholders incl. Government, program developers and coordinators, regulatory bodies, professional associa- tions, training and research institutions, health facilities, United Nations and other funding agencies, and non- governmental organizations involved in the improvement of newborn care or the implementation of KMC (*n* = 11–13/ country). Health facilities (*n* = 39; 3 teaching, 4 regional, 23 districts, 4 non profit, 1 rural, 4 health centres) 2. Health facilities in Malawi, Mali, Rwanda and Uganda	1. Implementation framework (6 stages) 2. Evaluation framework 3. Level 5
1. Bernstein 2. 2009 [34] 3. MMS 4. USA	1. Reports the dissemination and evaluation of SBIRT on systems of care in EDs using RE-AIM framework 2. Implementation	1. 24 participants incl. HPAs and their supervisors, clinicians, nurse managers, and ED directors 2. Five ED	1. Knowledge translation framework (RE-AIM) 2. Evaluation framework 3. Level 5
1. Bhanbhro 2. 2016 [35] 3. QS 4. UK	1. To explore the factors associated with variation between ‘units’ in sustaining the intended recovery-oriented practice during the recovery-focused staff training intervention (GetREAL) 2. Non-sustainability	1. Team on unit incl. Psychiatrist, psychologist and OT. Some exec management (ward manager, senior service manager, unit manager) attended ward training. Management support measured. Reaction of service users to intervention also reported. Three units: 2 hospital and 1 community, no. beds range: 15–31 2. Mental health rehabilitation units	1. CMO 2. Evaluation framework 3. Level 5
1. Bouamrane and Mair 2. 2014 [36] 3. QS 4. Scotland	1. To analyse the perspectives of key stakeholders involved in the rationalisation of surgical pre-assessment clinics (PACs) in NHS GGC and the integrated care pathway (ICP) design, development and implementation; identifying the complex sociotechnical factors that have influenced the successful adoption of the electronic preoperative ICP across NHS GGC in order to inform future implementations in this sphere 2. Implementation	1. 3 main stakeholder interviews: eForm 1: a member of the NHS GGC electronic patient record programme (EPR) eForm team involved in the development of design requirements and technical specifications for the preoperative ICP, −Anaesthetist 1: a consultant anaesthetist involved in the consensus process which led to development of the structured clinical content of the preoperative ICP, including the selection of guidelines underpinning the context dependant, adaptive behaviour of the eForm. -POA nurse 1: a senior nurse involved in the PCIP review of the NHS GGC PACs and the dissemination of information relating to the programme implementation across the health-board. In addition, the nurse was involved in the eForm user-testing, reporting user requirements and change requests to the eForm development team. 1 case study interviewing the service lead nurse and 3 nurses working in the clinic. 2. Acute Care hospital, pre-op clinics.	1. NPT 2. Implementation theory 3. Level 5
1. Brady 2. 2014 [37] 3. QI 4. USA	1. To increase the proportion of patients with acute haematogenous osteomyelitis admitted to the hospital medicine service who were discharged on oral antibiotics within 120 days. 2. Implementation	1. 12 hospital medicine faculty and 53 residents and medical students. Education targeted at medical faculty, residents, students. Wider aim was to increase rapid adoption of evidence-based decision making, and value in general paediatrics as a model of spread across city’s health care system and beyond. 2. Academic Children’s hospital	1. List of key drivers (i) Knowledge and implementation of evidence for osteomyeltis treatment (ii) Local expert opinion and treatment (iii) Understanding among hospital medical team which patients need consults (iv) Physician ordering system and decision support for evidence-based care (v) Engagement of family and patient in shared decision making (vi) Physician feedback on performance and outcomes (identify and mitigate) (vii) Engagement of community physicians 2. Process model 3. Level 5
1. Bridges 2. 2017 [38] 3. QS 4. England	1. To more thoroughly investigate the process of implementing an intervention aimed at supporting the delivery of compassionate care by hospital teams; to identify and explain the extent to which CLECC was implemented into existing work practices and to identify how CLECC can be optimised to support sustained compassionate care delivery in acute settings. 2. Sustainability	1. Wards: older people (3), trauma and orthopaedics (1). Participants: 25- ward managers (4), deputy ward managers (2), staff nurses (8), healthcare assistants (7), senior hospital nurses (2), PDNs (2) 2. Four inpatient wards in 2 general hospitals	1. NPT 2. Implementation theory 3. Level 5
1. Campbell 2. 2011 [39] 3. QS 4. Canada	1. To understand how hospitals using the Ottawa Model for Smoking Cessation (OMSC) addressed sustainability, and determine if there were critical factors that should be addressed before expansion across Canada. 2. Sustainability	1. Six hospitals. One decision maker and one smoking cessation coordinator at hospital with 2 exceptions (1 DM at one hopsital and 2 DMs at one hospital). DMs held senior administrative roles such as director, clinical manager, chief nursing officer. SCCs were 4 unit nurses, 1 program manager and 1 dedicated SCC. Not all of these were involved in the initial program implementation. 2. Three general inpatient unit and 3 special care units	1. OMSC 2. Determinant framework 3. Level 5
1. Fleiszer 2. 2015 [40] 3. QS 4. Canada	1. How a nurse best practice guidelines (BPG) program was sustained over a long period of time in an acute healthcare centre: 1. How was program sustainability characterised? 2. What factors influenced sustainability? 3. How was the program sustained? 2. Sustainability	1. 14 organisational key informants (all registered nurses). 350 documents. 40 observations and exchanges. Nursing department level of the organisation. Acute academic health centre incorporating 6 hospital sites. Best practice guidelines (BPG) examined from executive level to front line level of the acute health centre. 2. Nursing department of an acute health centre	1. Developed their own conceptual framework proposing 3 charactieristics of sustainability (i.e. benefits, institutionalization and develoment) influences from 4 chacategories of factors (i.e. innovation, context, leadership and process) and relationships between characteristics and factors. 2. Determinant framework 3. Level 5
1. Fleiszer 2. 2016 [41] 3. QS 4. Canada	To understand how a nursing program was sustained over a long-term period in an acute healthcare center. 1. How was program sustainability characterized; 2. What were the factors that most influenced program sustainability; and 3. How was the program sustained over the long-term? 2. Sustainability	1. 4 inpatient nursing units. 25 interview participants. Sustainability examined at nursing department level of the health centre and then across 4 unit subcases. Looked at organizational/unit contexts 2. Hospital (a large tertiary/ quaternary urban academic health centre) As described in Fleiszer 2015.	1. Developed their own framework (as described in Fleizer 2015 paper) 2. Determinant framework 3. Level 5
1. Frykman 2. 2017 [42] 3. QS 4. Sweden	1. The aim of this study was to uncover the mechanisms influencing the sustainability of behavior changes following the implementation of teamwork at an ED 2. Sustainability	1. Participants for interviews: 2 physicians, 2 RNs, and 2 LPNs. 2.Emergency Department, Internal medicine at a university hospital	1. Integrated theoretical framework DCOM® Johnson et al. 2008 ^i^ 2. Implementation theory 3. Level 5 i. Johnson J, Dakens L, Edwards P, Morse N. SwitchPoints: Culture Change on the Fast Track to Business Success. John Wiley & Sons, Hoboken, NJ.
1. Glasgow 2. 2013 [43] 3. MMS 4. USA	To examine how a collection of survey measures of hospital characteristics related to QI success during a QI collaborative 2. Implementation	1. 100 hospitals. Survey 1: *n* = 130 participants, survey 2: *n* = 160 participants 2. Veterans hospitals providing inpatient care	1. General systems engineering model 2. Determinant framework 3. Level 5
1. Gould 2. 2016 [44] 3. QS 4. Wales	1. To explore the meaning of IPC ownership to health workers, and to evaluate the impact of an action plan to encourage IPC and IPC ownership throughout a National Health Service (NHS) health board in Wales, UK. 2. Implementation	1. 20 participants (7 doctors, 8 nurses, 3 general managers, 1 cleaner) and individuals involved in infection prevention and control 2. Acute care in four hospitals	1. NPT 2. Implementation theory 3. Level 4
1. Gramlich 2. 2017 [45] 3. QS 4. Canada	1. What are the barriers and enablers to ERAS implementation within a health system? 2. Implementation	1. 15 patients, 56 nurses, 13 clinical nurse educators, 1 unit clerk, 2 patient safety officers, 16 surgeons, 12 anaesthetists, 6 dietitians, 31 unit managers, 1 occupational therapist, 1 physiotherapist, 1 enterostomal therapist, 33 AHS (Alberta Health Services) managers, 6 site coordinators, 3 internal medicine doctors, 5 knowledge consultants, 3 pharmacists 2. Surgery units in 6 hospitals in the Alberta Health Services	1. TDF and QUERI 2. Determinant framework 3. Level 5
1. Green 2. 2017 [46] 3. QS 4. England	1. To identify factors that supported the successful implementation of two care bundles in the acute medical setting that used quality improvement methods. 2. Implementation	1. Data sources: progress review meetings and review reports and audio recordings of the review meetings 2.Acute medical unit/ward in 2 hospitals	1. CFIR 2. Determinant framework 3. Level 3
1. Hommel 2. 2017 [47] 3. QS 4. Sweden	1. To explore successful factors to prevent PUs in hospital settings. 2. Implementation	1. Six hospitals, 39 persons (managers, physicians, registered nurses, enrolled nurses with different kind of responsibilities) 2. Hospitals	1. PARIHS and Hsieh and Shannon (2005)^i^ 2. Determinant framework 3. Level 3 i.Hsieh HF & Shannon SE. Three approaches to qualitative content analysis. 2005. Qualitative Health Research, 15, 1277–1287.
1. Hovlid 2. 2012 [48] 3. QS 4. Norway	1. Not explicitly stated but to explore factors contributing to sustained improvement 2. Sustainability	1. 20 (9 physicians, 7 nurses, 2 secretaries, 2 administrators) 2. Surgical departments (ophthalmology, general surgery, gynaecology, orthopaedics, ENT) at a District General Hospital	1. ELO 2. Process model 3. Level 3
1. Ilott 2. 2016 [49] 3. QS 4.England	1. To understand the processes, mechanism and outcomes associated with the spread and sustainability of a safety initiative 2. Sustainability	1. 7 wards (5 in hospitals, 2 in community). 22 front-line staff, 12 trainers. 2.see (3) 3.Data collected at the organisational and clinical level. There were senior managers with an organisation-wide remit. These are referred to as Education Strategic Leads (ESL) and Professional Strategic Leads (PSL). On the care pathways, there were Clinical Leads (CL), Education Leads (EL) and Trainers (T) who completed the train-the-trainer course. 4. Hospitals and community	1. Frameworks for spread and sustainability 2. Determinant framework 3. Level 5
1. Jangland and Gunningberg 2. 2017 [50] 3. MMS 4. Sweden	1. To conduct an evaluation of an implementation project on patient participation, using two specific research questions: How did the patients report their perception of quality of care, with a specific focus on patient participation after the implementation project? How did the nurse managers describe patient participation and their learning experience from the implementation project in the unit? 2. Non-sustainability	198 patients; The patients’ mean age was 61.6 years (range 23–92, SD 15.4), the gender distribution was even and the majority stayed in the surgical care unit between 2 and 6 days. 5 nurse managers (41 to 48 years of age (mean 45 years) and had held their position in their unit from 2 to 16 years (mean 6 years). They were all RNs (1–10 years’ experience; mean 8.5 years). 2. Surgical department in a large hospital	1. PARIHS 2. Determinant framework 3. Level 5
1. Matthew-Maich 2. 2013 [51] 3. QS 4. Canada	(1) What processes are involved in the implementation and uptake of the RNAO Breastfeeding BPG in three acute care hospitals? (2) What is the impact of the BPG implementation and uptake for clients, nurses, other professionals, units, organizations and the broader system? 2. Sustainability	1. maternal-child units in three diverse acute care hospitals. 112 participants (54 mothers and 58 health professionals). 58 health professionals - 32 staff nurses, administrators and managers (7), lactation consultants (5), educators (5), physicians (3), midwives (3) and public health nurses (3). 2. Acute care hospital sites	1. SUNG 2. Implementation theory 3. Level 5
1. Mazzocato 2. 2012 [52] 3. MMS 4. Sweden	1. The objectives of the quantitative component were to track operational performance changes over time and to compare performance before and after the lean intervention. The objectives of the qualitative component were both to describe the lean intervention and to provide data to help us explain how the intervention worked based on four theoretical lean principles. 2. Implementation	1. n = 13 (1 resident, 3 senior physicians, 3 nurses, 1 coach, the director of the pediatric division, 2 first line managers, 2 administrative staff members) 2.Paediatric A&E at a hospital	4. Theoretical LEAN principles, empirically (derived by Spear and Bowen^i^). According to these principles, LEAN (a) standardizes work and reduces ambiguity (b) connect people who are dependent on one another (c) creates seamless, uninterrupted flow of work through the process and (d) empowers staff to investigate process problems and to develop, test and implement countermeasures using a “scientific method”. 2. Determinant framework 3. Level 5 i.Spear S, Bowen HK. Decoding the DNA of the Toyota Production System. Harvard Business Review 1999, 77 (5):96–106.
1. McClung 2. 2017 [53] 3. QS 4.USA	1. To examine health care worker motivation for reducing HAI 2. Implementation	1. 10 respondents (6 physicians, 2 nurses, 1 nursing assistant, and 1 manager of environmental services, and the respondents came from a variety of departments, including internal medicine, critical care, hematology oncology, general surgery, and orthopedic surgery. Three physicians held administrative roles, including 2 within quality improvement efforts in the hospital. Two physicians held HAI champion roles, including surgical site infection, CAUTI, and CLABSI, whereas 1 physician with an administrative role also held a champion role. The nursing personnel, including the nursing assistant, also held similar champion roles in CDI and CAUTI). 2. Large academic research institution with 592 staffed beds and a level 1 trauma centre	1. CFIR 2. Implementation theory 3. Level 5
1. Mitchell 2. 2017 [54] 3. QS 4. USA	1. characterizes contextual factors influencing their decision-making process and motivations behind adaptations of the RED protocol and the impact of context and adaptations on implementation and sustainment of RED in these settings 2. Sustainability	1. 5 hospitals (suburban/urban, 2 suburban, 2 urban). 64 participants (11 senior leadership/executive, 22 clinical implementation team, 19 non-clinical implementation team, 9 non-RED staff, 3 community based partners) 2. Hospitals	1. Conceptual model of contextual factors 2. Determinant framework 3. Level 5
1.Naldemirci 2. 2017 [55] 3. QS 4.Sweden	1. To explore the deliberate and emergent strategies of key stakeholders to specific contextual challenges encountered when implementing the GPCC framework 2. Sustainability	1. 18 researchers, 17 healthcare practitioners (5 registered nurses, 4 assistant nurses, 4 ward managers, 4 physicians). Patients (20) who had recently been hospitalised. 2.Hospital wards	1. Mintzberg & Water’s taxonomy of types of strategies^i^ and NPT 2. Implementation theory 3. Level 3 i. Minzberg H, Walter, J. Of Strategies, Deliberate and Emergent. Strateg Manag J. 1985;6 (3):257–72.
1. Nordmark 2. 2016 [56] 3. QS 4. Sweden	1. The aim of this study was to explore the embedding and integration of the DPP from the perspective of registered nurses (RNs), district nurses (DNs) and homecare organizers (HCOs). 2. Implementation	1. Five hospital wards with the highest frequency of DPs were identified: geriatric/palliative, infection, surgical, orthopaedic and pulmonary medicine/ endocrinology- gastrology.12 Registered Nurses 2. Hospital wards	1. NPT 2. Implementation theory 3. Level 5
1. Parand 2. 2012 [57] 3. QS 4.UK	1. Offering strategies that are reported to promote sustainability of an organizational safety improvement programme: the UK Safer Patients Initiative (SPI) 2. Implementation	1. 34 coordinators of the Safer Patients Initiative Programme: 20 interviews at the end of the programme and 14 a year later. Focus on sustainability of intervention across the organisation 2. UK NHS Hospitals	1. Model for Improvement plus PDSA cycles 2. Process model 3. Level 5
1. Robert 2. 2011 [58] 3. MMS 4. England	1. To explore the local adoption, implementation and assimilation of an innovation into routine nursing practice by applying an evidence-based diffusion of innovations framework to a national quality improvement programme 2. Implementation	1. Survey: 150 responses, 56 project leaders/facilitators, 19 manager of the PW, 14 working in the PW most of the time, 70 either a ward manager/ sister/ charge nurse, staff nurse or matron. Case studies: 58 2. Acute hospitals	1. Adapted the model produced by Greenhalgh et al. (2005)^i^ 2. Classic theory 3. Level 5 i. Greenhalgh T, Robert G, Bate SP, Macfarlane F & Kyriakidou O (2005) Diffusion of Innovations in Health Service Organisations. Blackwell, Oxford.
1. Rotteau 2. 2015 [59] 3. QS 4. Canada	1. To describe the hospital-based implementation teams’ experiences during program implementation, and the team’s perceptions of the key factors that influenced the program’s success or failure. 2. Implementation	1. 10 hospitals (6 with greatest improvement and 4 with least improvement), 52 participants (10 executive sponsors, 19 physician leads, 23 team leads) 2. Emergency Departments in hospitals with greatest (3 hospitals) and least (2 hospitals) improvement in wait times.	1. LEAN 2. Determinant framework 3. Level 2
1. Sanchez 2. 2014 [60] 3. QS 4. USA	1. To perform a qualitative examination of the medication reconciliation planning process in two healthcare organizations 2. Implementation	1. 13 interview respondents: 12 participating directly in the medication reconciliation planning process and one became involved after implementation was underway. Respondent roles: quality improvement (4), information technology (4), medication safety (3), and education (2). They had on average 5.9 (SD = 3.7) years of experience in their current position and all except one were present in their current position at the time the medication reconciliation implementation process had taken place. By professional training, there were four physicians, four nurses, four pharmacists, and one information technologist. 2. Large urban academic tertiary care center and an affiliated Veterans Affairs (VA) hospital in New York City	1. CFIR 2. Determinant framework 3. Level 4
1. Stacey 2. 2015 [61] 3. MMS 4.Canada	1. To evaluate a sustainable approach for implementing the lung transplant referral patient decision aid into clinical practice in adult cystic fibrosis (CF) clinics 2. Sustainability	1. 31 healthcare professionals (18 nurses, 12 physicians, 1 pharmacist) 2.Adult CF clinics within 8 different provincial healthcare systems in Canada (*n* = 18)	1. Knowledge-to-Action Framework 2. Process model 3. Level 5
1. White 2. 2011 [62] 3. QI 4. USA	1. To develop and implement a sustained medication reconciliation process to improve patient safety and compliance with Safety Goal 8. 2. Implementation	1. NA – obtained from weekly reports which merged admitting and registration information from the primary electronic medical record with data from the electronic medication reconciliation application. 2. Large urban paediatric academic medical centre	1. Model for improvement 2. Process model 3. Level 5

Category of implementation theory, model and framework as defined in Nilsen (2015) [29] (Table 1, p3):

**● Classic Theories**: defined as theories that originate from fields external to implementation science, e.g. psychology, sociology and organizational theory, which can be applied to provide understanding and/or explanation of aspects of implementation;

**● Determinant Frameworks**: defined as types (also known as classes or domains) of determinants and individual determinants, which act as barriers and enablers (independent variables) that influence implementation outcomes (dependent variables). Some frameworks also specify relationships between some types of determinants. The overarching aim is to understand and/or explain influences on implementation outcomes, e.g. predicting outcomes or interpreting outcomes retrospectively;

**● Evaluation frameworks**: defined as those frameworks that specify aspects of implementation that could be evaluated to determine implementation success;

**● Implementation theories**: Theories that have been developed by implementation researchers (from scratch or by adapting existing theories and concepts) to provide understanding and/or explanation of aspects of implementation;

**● Process models**: Specify steps (stages, phases) in the process of translating research into practice, including the implementation and use of research. The aim of process models is to describe and/or guide the process of translating research into practice.

Levels of theoretical visibility (see Bradbury-Jones 2014 [30]):

**●** Level 1 – Seemingly absent,

**●** Level 2 – Implied,

**●** Level 3 – Partially applied,

**●** Level 4 – Retrospectively applied,

**●** Level 5 – Consistently applied

Key: *CFIR* Consolidated Framework for Implementation Research, *CMO* Context-Mechanism-Outcomes, *DM* Decision Maker, *ED* Emergency Department, *ELO* Evidence in the Learning Organization, *ERAS* Enhanced Recovery After Surgery program for colonic surgery, *HPA* health promotion advocates, *KMC* Kangaroo Mother Care, *MDT* multidisciplinary team; *MMS* mixed methods study, *NA* not applicable, *NP* nurse practitioners, *NPT* Normalisation Process Theory, *OMSC* Ottawa Model for Smoking Cessation, *OT* occupational therapist, *PARiHS* Promoting Action on Research Implementation Framework, *PPIP* Perinatal Problem Identification Programme, *RE-AIM* Reach, Effectiveness, Adoption, Implementation, and Maintenance framework, *QS* qualitative study, *QUERI* Quality Enhancement Research Initiative, *SSP* short-stay program, *SUNG* Supporting the Uptake of Nursing Guidelines, *TDF* Theoretical Domains Framework

#### Sustainability

A universal definition of sustainability, despite best efforts, is still lacking [8, 63, 64]. To standardise our reporting of sustained studies, the review was guided by Moore’s work (2017) [8] which created a five-construct definition of sustainability from over 200 studies. This posits that sustainability is achieved:

1. after a defined period of time,
2. when the intervention of interest continues to be delivered and / or
3. the intended individual behavioural change is maintained, and
4. both (2) and (3) may evolve or adapt
5. while continuing to produce beneficial outcomes.

Two reviewers (ED, EAD) mapped each included study against each construct to indicate how comprehensively sustainability was reported.

#### Barriers and facilitators

A single, comprehensive tool for identifying the barriers and facilitators for sustained interventions is currently lacking. However, a number of frameworks already exist which focus on or allude to sustainability [1, 12, 65, 66]. In our protocol, we had originally planned to identify barriers and facilitators in each paper then code them to all of these frameworks for comparison. However, in this paper we present findings from the data coded to the Consolidated Framework for Sustainability Constructs in Healthcare [12] as it was judged by all of the reviewers to provide the most relevant and useful insight into sustainability in hospital settings. A methodological paper comparing the advantages and disadvantages of these frameworks using these core data will be published elsewhere.

**Fig. 1:**
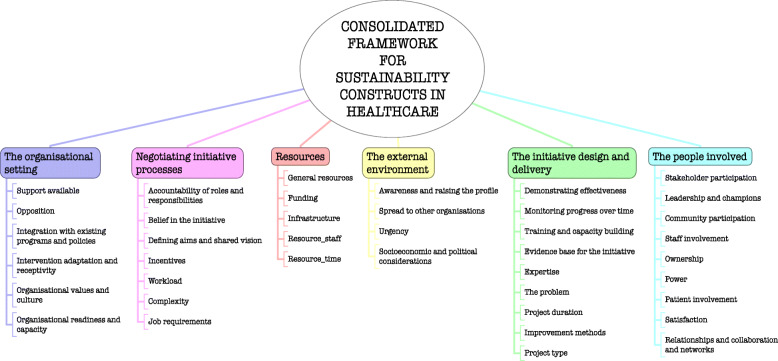
Consolidated framework for sustainability constructs in healthcare

We planned to take both deductive and inductive thematic approaches to identifying barriers and facilitators. The deductive approach used a predefined list of 40 constructs from the Consolidated Framework for Sustainability Constructs in Healthcare [12], for which Lennox et al. [12] provided helpful descriptions, definitions and examples in an additional file (see Fig. 1).

Data were initially extracted and categorised as either a barrier, facilitator or (rarely) neutral. Each was then coded according to the predefined constructs [12]. A second reviewer (JC, PC) cross-checked the data and coding. Barriers or facilitators that we could not categorise or find a best fit for using the predefined constructs were coded as ‘other’. An inductive approach was used to compare these additional data to develop additional constructs or principles important for sustainability in a hospital setting.

### Methodological quality assessment of included studies

Study quality was assessed independently by two reviewers, using tools appropriate to the design of the study (i.e.) the Critical Appraisal Skills Programme [67] for qualitative studies, Mixed Methods Appraisal Tool [68] for mixed method and quantitative studies, and Standards for Quality Improving Reporting Excellence [69] for quality improvement studies. All studies, regardless of methodological quality, that met the selection criteria were included in the data synthesis.

### Data synthesis

Descriptive data (i.e., year, country, professional groups involved, hospital setting and other contextual factors, theoretical frameworks, and sustainability factors) were tabulated within evidence tables. We did not plan to conduct a meta-analysis as we had anticipated that it would not be possible to pool data due to the heterogeneity between studies and outcomes. Key findings were instead brought together within a narrative synthesis.

Evidence relating to barriers and facilitators to sustainability of hospital-based interventions were brought together using a narrative synthesis supported by tables and figures organised around the six themes reported in Lennox et al. (2018) Consolidated Framework for Sustainability Constructs in Healthcare [12]. This included: (1) Initiative design and delivery (see Fig. 5, Additional file 9); (2) Negotiating initiative processes (see Fig. 6, Additional file 10); (3) The people involved (see Fig. 7, Additional file 11); (4) Resources (see Fig. 8, Additional file 12); (5) The organisational setting (Fig. 9, Additional file 13) and (6) External environment (see Fig. 10, Additional file 14).

## Results

### Study selection and characteristics

**Fig. 2:**
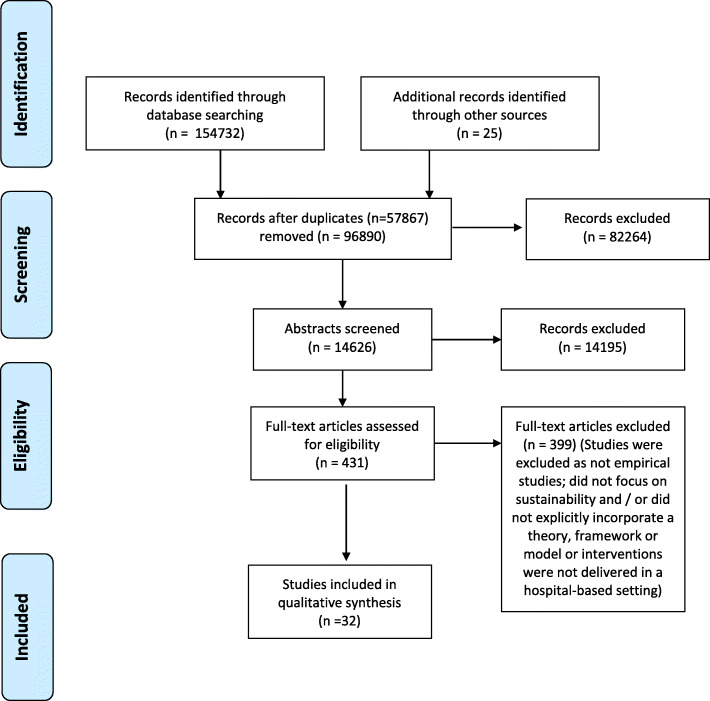
Flowchart of records identified for relevant studies for inclusion in the review

Our search identified 154,757 records. Figure 2 shows the flow of literature throughout the study. We screened 14,626 abstracts, retrieved 431 full text papers, of which 32 studies met the selection criteria [31–62]. The key characteristics of the included studies are summarised in Table 1.

Most of the studies were conducted in the UK (*n* = 8/32) [35, 36, 38, 44, 46, 49, 57, 58] or in the USA (*n* = 8/32) [34, 37, 43, 53, 54, 60–62]. Other studies were conducted in Canada (*n* = 6/32) [39–41, 45, 51, 59], Netherlands (*n* = 1/32) [31], Norway (*n* = 1/32) [48], South Africa (*n* = 2/32) [32, 33], and Sweden (*n* = 6/32) [42, 47, 50, 52, 55, 56] (Table 1).

The majority of included studies (23/32) employed a qualitative design [31, 32, 35, 36, 38–42, 44–49, 51, 53–57, 59, 60]. Seven studies employed a mixed methods design [33, 34, 43, 50, 52, 58, 61] and two studies used quality improvement methods [37, 62]. A variety of methods were used including semi-structured interviews, focus groups, observation, and surveys. Methods are detailed in Additional file 3.

Fifteen studies examined sustainability by drawing on established implementation frameworks including the Normalisation Process Theory (NPT) [36, 38, 44, 55, 56]; Consolidated Framework for Implementation Research (CFIR) [31, 46, 53, 60]; Promoting Action on Research Implementation in Health Services (PARiHS) framework [47, 50]; Reach, Effectiveness, Aim, Implementation and Maintainence (RE-AIM) framework [34]; Knowledge-to-Action Framework [61]; Greenhalgh et al’s 2005 Model of Diffusion of Health Service Innovations [58] and Quality Enhancement Research Initiative (QUERI) [45].

Seven studies drew on frameworks associated with improvement science including Stages of change [32, 33]; Model for Improvement [37, 62]; General systems engineering model [43]; Evidence in the Learning Organisation model [48] and LEAN continuous improvement principles [52].

The remaining ten studies examined sustainability either by employing a specific framework or developing one through the research approach including Gruen’s Sustainability Model [39]; Buchanan et al’s process of sustainability in context [49]; Conceptual framework for the sustainability of healthcare innovations [40, 41]; Supporting the Uptake of Nursing Guidelines (grounded theory) [51]; Realistic evaluation (context-mechanism-outcome configurations) [35, 42] and Bespoke frameworks with inductive analysis [54, 57, 59].

Three authors combined the primary framework with additional frameworks to achieve their intended aim [45, 48, 49]**.**

### Participant characteristics

Studies were conducted in a variety of hospital settings, some more generic than others. They included: acute care (*n* = 3/32) [44, 46, 51]; surgical care (*n* = 4/32) [31, 45, 48, 50]; emergency departments (*n* = 4/32) [34, 42, 52, 59]; inpatient units (*n* = 4/32) (e.g. Cystic Fibrosis [61], mental health rehabilitation [35], paediatric units [37, 62]); hospitals (e.g. general hospital wards) (*n* = 4/32) [38, 39, 47, 58] and across a combination of hospital settings (*n* = 13/32) [32, 33, 36, 40, 41, 43, 49, 53–57, 60] (Additional Table 4).

The majority of studies (27/32) involved a combination of participants from frontline and higher organisational levels (unit, ward, department) [31, 33, 35–46, 48, 49, 52–62]. The remaining five studies were aimed at individual healthcare professionals or key stakeholders [32, 34, 47, 50, 51] (Table 1).

### Study quality assessment

#### Qualitative studies

Quality appraisal judgements for qualitative studies (*n* = 23/32) [31, 32, 35, 36, 38–42, 44–49, 51, 53–57, 59, 60] are presented in Additional file 5. All of the qualitative studies clearly reported 9/10 CASP criteria [31, 32, 35, 36, 38–42, 44–49, 51, 53–57, 59, 60]. However a consistent exception was the underreporting of the relationship between researcher and participants in 13/23 qualitative studies [31, 35, 36, 38, 40, 41, 48, 51, 53–55, 57, 59].

#### Mixed-methods studies

Four of the studies [34, 43, 50, 58] that employed a mixed-method design were judged to have clearly reported across all of the criteria outlined in the MMAT tool [68]. In the remaining three studies [33, 52, 61], most of the criteria were clearly reported, however two studies did not discuss the potential for researchers influence on the qualitative data [33, 52] and potential recruitment bias was identified in one study [33]. Insufficient details were also reported in these three studies on some criterion which meant that we were unable to make a judgement about whether the qualitative data analysis was relevant in Bergh (2014) [33]; or whether the response rate in Mazzacato et al. (2012) [52] was acceptable; or whether the groups were comparable in Stacey et al. (2015) [61]. Detailed quality assessments for studies employing a mixed method or quantitative design (*n* = 7/32) [33, 34, 43, 50, 52, 58, 61] using the MMAT tool [68] are shown in Additional file 6.

#### Quality improvement studies

All of the 18 items from the SQUIRE statement [69] were reported in the quality improvement studies [37, 62] (Additional file 7); however the funding statement in White et al. (2011) [62] was not specifically reported. Quality assessment for studies using a quality improvement method (*n* = 2/32) [37, 62] using SQUIRE [69] are presented in Additional file 7.

### Interventions

Intervention components, delivery regime and key findings from each of the included studies are shown in Additional file 4. The aims of the interventions broadly fell into one of three categories: (1) to enhance the quality of patient care and/ or safety (*n* = 22/32 studies) [31–33, 35–41, 45–47, 49–51, 55, 57, 58, 60–62] (2) to improve flow of patients through the hospital by reducing waiting times, shortening the length of stay or improving discharge planning (*n =* 7 studies) [42, 43, 48, 52, 54, 56, 59] or (3) improving hospital processes (e.g. screening and referrals or reducing healthcare infections and improving infection control (*n* = 3 studies) [34, 44, 53].

All of the interventions / or programmes delivered were multicomponent, and used several different modes of delivery. A diverse range of stakeholders were frequently involved in the development and delivery of the intervention alongside a variety of frontline health care professional groups (Additional file 4). Interventions were reported as tailored to patient needs and/or local factors in 25/32 studies [31, 33, 34, 37–42, 45–51, 54–62]. Fourteen studies reported that interventions were modified, however specific details about the changes to the intervention, and when this took place, were often limited [31, 33, 34, 37, 42, 45, 48, 49, 52–56, 59].

#### Interventions that aimed to to enhance the quality of patient care and/ or safety

The majority of sustained interventions sought to improve patient care and /or safety using a variety of interventions (*n* = 22/32 studies) [31–33, 35–41, 45–47, 49–51, 55, 57, 58, 60–62].

Belizan et al. (2011) [32] introduced Health Care Professionals (HCPs) to an audit tool with a feedback system identifying where deaths had occurred to help improve perinatal care. Best practice guidelines and policies were the basis of the intervention delivered in Matthew-Maich et al. (2013) [51] to improve breastfeeding. Bergh et al. (2014) also delivered a context-appropriate, outreach Kangaroo-care intervention using training and education to improve breastfeeding and mother-infant care outcomes.

Implementation of nursing best practice guidelines for falls prevention, pressure ulcer prevention, and pain management were the basis of the intervention described in Fleiszer (2015, 2016) [40, 41]. The intervention described in Hommel et al. (2017) involved nurse coaches implementing multicomponent interventions also aimed at improving pressure ulcers in hospitals based on clinical guidelines [47]. Green et al. (2017) also sought to improve patient care and described two initiatives: a) COPD care bundle and b) diabetic foot care bundle. Parand et al. (2012) described a collaborative methdology to implement a large scale complex intervention aimed at improving patient safety by standardising care and reducing variation in practice. They used collaborative learning, improved data sharing mechanisms, and employed a “buddy” system. Campbell et al. (2011) [39] delivered a hospital based smoking cessation program which involved identifying smokers on admission, documenting smoking status, offering support to quit and following up at discharge. This was a national initiative led locally by a dedicated smoking cessation co-ordinator.

Two other studies reported interventions based on an evidence based guideline program called the Enhanced Recovery after Surgery (ERAS) care system for colonic surgery [31, 45]. These guidelines are a bundle of 22 interventions that are delivered variously before, during and after surgery. Ament (2017) [31] also reported another program called the short-stay program for breast cancer surgery, which sought to increase efficiency of breast cancer surgery care by renewing the patient information strategy, standardising the care processes, while maintaining the perceived quality of care by patients.

The intervention decribed in Naldermirci (2017) [55] focused on developing a person-centred care plan with patient and carers within 12–24 h after admission or at the first outpatient attendance. Jangland Gunningberg (2017) [50] also reported the use of a person-centred care plan to improve patient-healthcare professional communication in surgical care units. Patients were encouraged to use “tell-us” cards as a tool for documenting their concerns and listing their daily goals. The intervention described in Stacey et al. (2015) [61] involved the use of a patient decision aid to help adults living with cystic fibrosis make decisions about lung transplantation.

Robert (2011) [58] described a national quality improvement study aimed at improving nurse-patient contact time drawing on LEAN principles to reduce activities that don’t add value or making changes to the ward space. Bhanbhro et al. (2016) [35] reported an intervention called “GetREAL” which aimed to increase the confidence and skills of staff working in inpatient mental health rehabilitation units in engaging service users in activities. The intervention was supported by the use of an intervention manual, a fidelity checklist, an induction programme and training materials.

Bridges et al. (2017) [38] used a workplace educational intervention, focused on developing sustainable leadership and work-team practices (dialogue, reflective learning, mutual support, role modelling), designed to support team relational capacity and compassionate care delivery. This multicomponent intervention used regular meetings, action plans, climate analysis and values clarification; peer observations of practice; team study days; mid-shift 5 min cluster discussions; and twice weekly reflective discussions.

Ilott et al. (2016) [49] aimed to raise awareness of dysphagia as a safety issue ensuring that any staff member working with dysphagic patients has the knowledge and skills needed to support safe swallowing.. A “train-the-trainer” intervention was delivered on the ward based on input from speech and language therapists who provided 3 h training alongside teaching resources, online learning modules and a toolkit.

In White et al. (2011) an improvement team plus a quality improvement consultant and data analyst worked with ward-based nurses and doctors to improve medication reconciliation at admission for inpatient services. They described implementing a multi-component tool using electronic medication reconciliation tool, improved electronic communication processes, reminders and education tools. Medical reconciliation aimed at reducing prescribing errors was also a key part of the intervention in Sanchez (2014) [60] and involved three steps: verification, clarification and documenting any changes. Brady (2014) [37] also describe a bundle of seven interventions including timely patient identification and staff education, which aimed at increasing the number of children with osteomyelitis leaving the hospital on oral antibiotics as opposed to antibiotics given intravenously.

Boumrane and Mair (2014) implemented an electronic preoperative integrated care pathway (eForm) allowing all hospitals to access a comprehensive patient medical history via a clinical portal on the health-board intranet. This electronic pathway resulted in a streamlined, standardised and integrated preoperative assessment process [36].

#### Interventions aimed at improving the flow of patients through the hospital

Seven studies sought to reduce waiting times, length of stay or improving discharge planning [42, 43, 48, 52, 54, 56, 59]. Hovlid (2012) [48] asked 40 healthcare professionals to redesign the elective surgery pathway in order to reduce the number of cancellations. Changes implemented included refining referral entry points, earlier clinical assessments, improvements in communication and information flow and agreement with patients about selection of their date for their surgery. Mazzocato reported a similar approach to staff-wide involvement in improving patient flow in the paediatric emergency department. The LEAN inspired intervention included changes in work schedules, new roles and job descriptions, team problem solving alongside regular monthly meetings with management groups [52]. Rotteau et al. (2015) [59] also aimed to improve the flow of patients in the emergency department using LEAN based quality improvements and a dedicated hospital improvement team. The interventions included system wide pay-for performance incentives, monthly reports of common data sets and setting targets for length of stay. External LEAN coaches were appointed to train and mentor improvement teams. Glasgow (2013) [43] also reported the use of improvement coaches to work with quality improvement teams to help improve in-patient hospital flow. This was a national programme involving 26 hospitals who were tasked with identifying a solution to each hospital's flow concerns guided by the VA–TAMMCS improvement framework (i.e. vision analysis, identifying a team, developing clear aims, flow mapping, and running plan, do, study, act change cycles followed by working to sustain and spread improvements).

A multicomponent intervention aimed at improved discharged planning to reduce the need for readmission was described in Mitchell (2017) [54]. They used a combination of patient education, identifying patient language needs, and planning for follow-up appointments including follow-up telephone calls to “reinforce” the discharge plan.

Nordmark [56] used technology solutions (e.g. shared calendars, video-conferencing, electronic information systems) to improve the discharge planning process. This was implemented by the registered nurse at the hospital who provided continuity of care by performing the discharge plan from admission to discharge.

The implementation of multi-professional team work, a behaviour-change intervention reported in Frykman (2013) which sought to reduce waiting times. The intervention involved room allocation, meetings at the start and end of each shift with reflection and feedback. External performance consultants assisted the dedicated change facilitator.

#### Interventions aimed at improving hospital processes

Three studies sought to improve hospital processes and infection control [34, 44, 53]. In Bernstein et al. (2009), local champions across seven sites used a brief intervention technique (based on motivational interviewing) to develop action plans and referrals for patients with substance abuse treatment that were identified in the emergency department [34]. Champion groups were also used in Gould (2016) to improve infection control and promote improved hand hygiene behaviours through a targeted multicomponent action plan with clear targets for reducing infection (e.g. deep cleaning, hand hygiene campaign, monthly metrics) [44]. A similar approach was reported in McClung (2017) [53] who used healthcare bundles to improve adherence to evidence based practices aimed at reducing healthcare associated infections.

### Theoretical frameworks

**Fig. 3:**
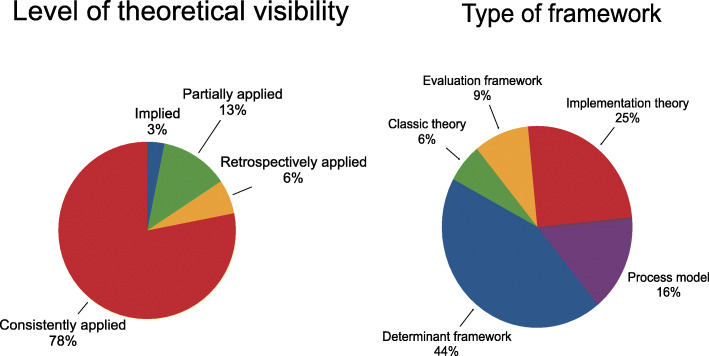
**a** Level of theoretical visibility; **b** Framework categorisation

The majority of studies (24/32) were judged to have consistently applied and articulated the chosen framework (Fig. 3a) (Table 1). However, because the use of different terminology to describe theoretical approaches is often confusing, we mapped the identified frameworks from each of the included studies against Nilsen’s taxonomy of theories, models and frameworks [29] to bring some clarity about the different types of frameworks that have been described in the included studies. The definitions for each of the five categories are summarised in Table 1. Two independent reviewers judged 14/32 studies as using a determinant framework [31, 32, 39–42, 46–48, 50, 53, 54, 58, 60] (Fig. 3b). The remaining studies were judged as implementation theory (*n* = 8/32) [36, 38, 42, 44, 51, 53, 55, 56]; process model (*n* = 5/32, 48, 54, 55, 58, 65]; evaluation framework (*n* = 3/32) [33–35] and classic theory (*n* = 2/32) [32, 58].

### Sustainability

**Fig. 4:**
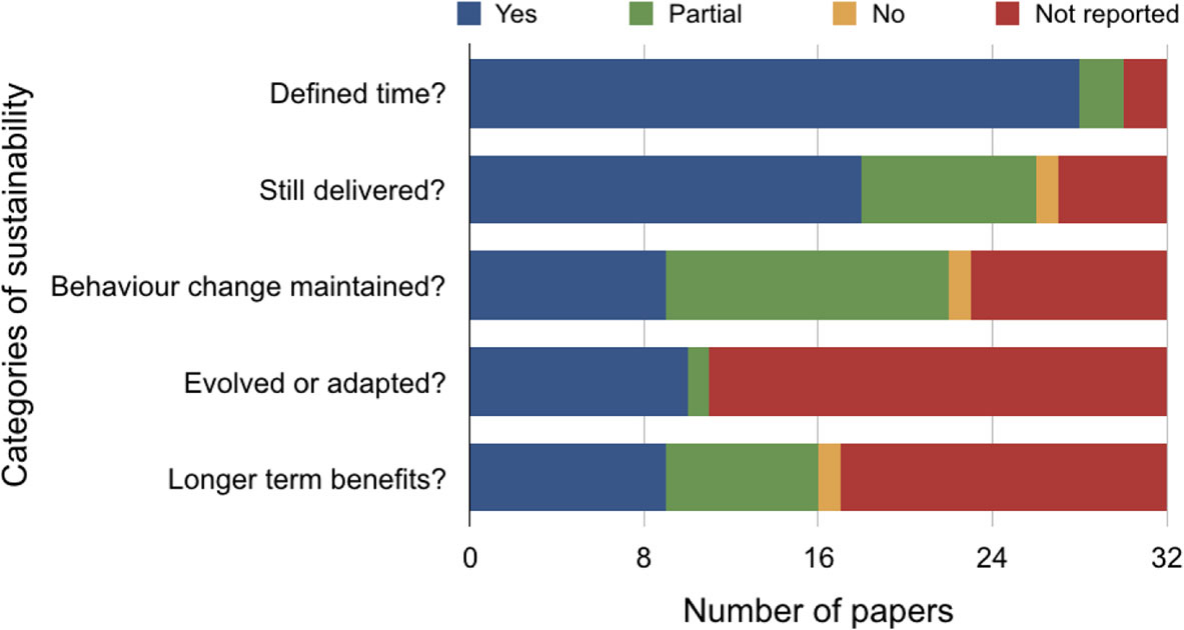
Bar chart showing the included studies mapped to the five constructs of sustainability

Two studies [36, 40] reported sustainability across all five key constructs identified by Moore’s (2017, 8] definition of sustainability. Figure 4 shows a graph of the studies mapped to the five constructs of sustainability. The length of time interventions were sustained was clearly documented in 28/32 studies, and ranged from 6 months [46, 51, 59] – 8 years [40, 41] (Additional file 8).

Interventions, programs and/or implementation strategies continued to be delivered in 18/32 studies and individual behaviour was reported as maintained in 9/32 studies (Additional file 8). Interventions were also reported to continue producing longer term benefits for individuals and / or systems in 9/32 studies and were reported to have evolved or adapted in 10/32 studies (Additional file 8).

### Barriers and facilitators

Barriers and facilitators that influenced the delivery of sustained healthcare interventions in hospital-based settings were reported across all included studies [31–62]. Multiple barriers and facilitators were identified within each study, and were extracted and mapped to the 40 sustainability constructs across the six themes from the Lennox (2018) Consolidated Framework for Sustainability Constructs in Healthcare (Fig. 1) [12].

**Fig. 5:**
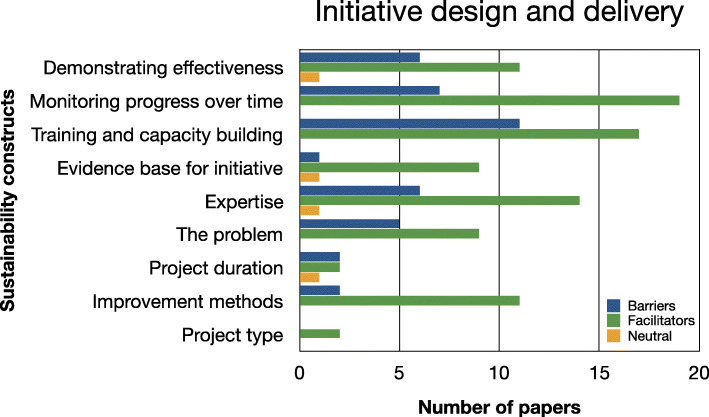
Bar chart showing the volume of evidence for barriers and facilitators reported within the initiative design and delivery theme

**Fig. 6:**
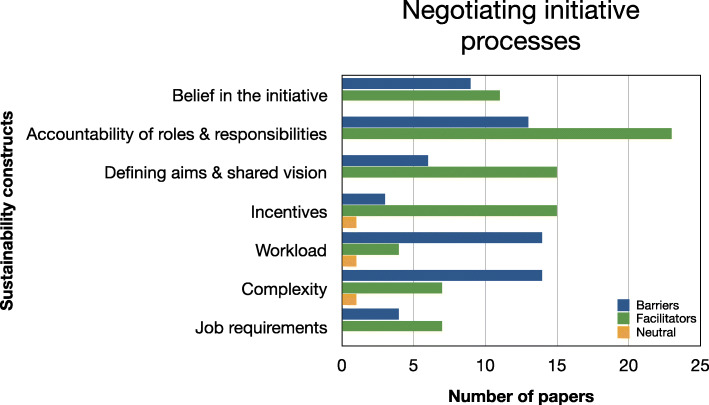
Bar chart showing the volume of evidence for barriers and facilitators reported within the negotiating initiative processes theme

**Fig. 7:**
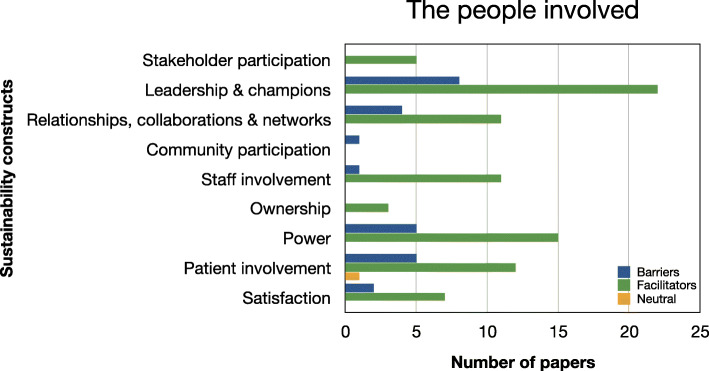
Bar chart showing the volume of evidence for barriers and facilitators reported within the people involved theme

**Fig. 8:**
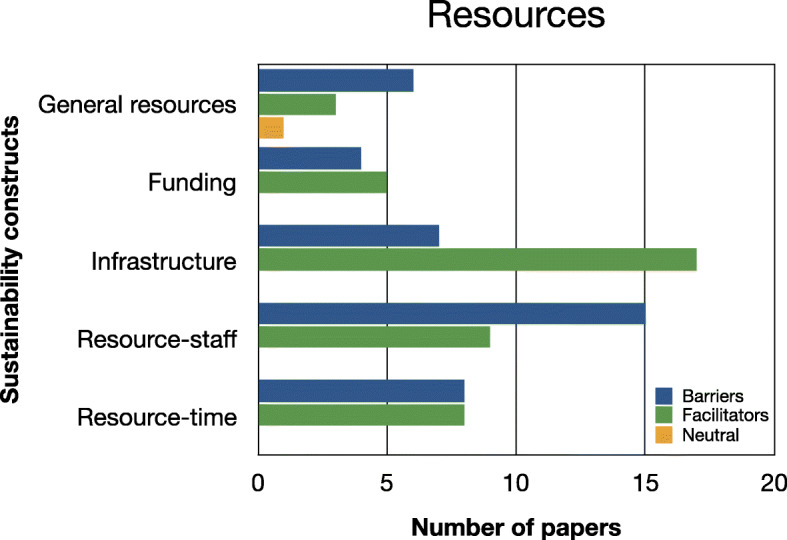
Bar chart showing the volume of evidence for barriers and facilitators reported within the resources theme

**Fig. 9:**
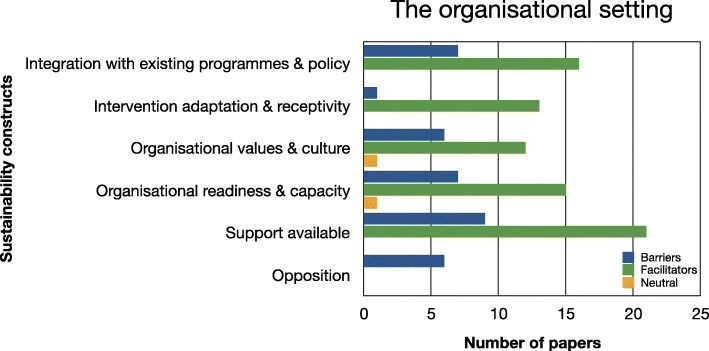
Bar chart showing the volume of evidence for barriers and facilitators reported within the organisational setting theme

**Fig. 10:**
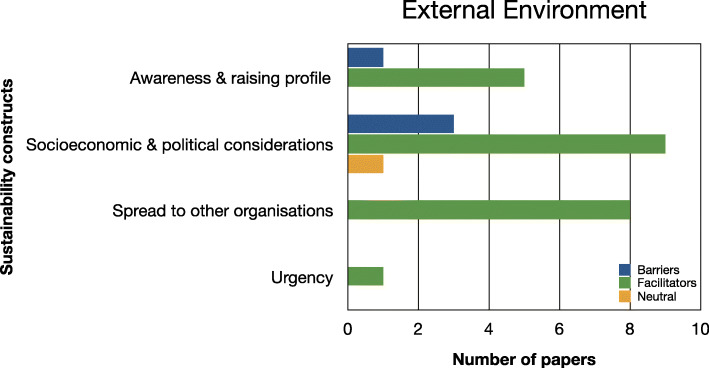
Bar chart showing the volume of evidence for barriers and facilitators reported within the external environment theme

**Fig. 11:**
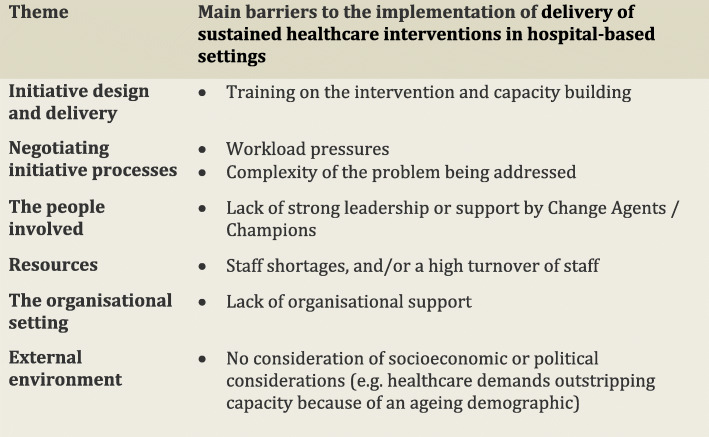
Key barriers reported within each of the themes from the Lennox (2018) Consolidated Framework for Sustainability Constructs in Healthcare [12]

**Fig. 12:**
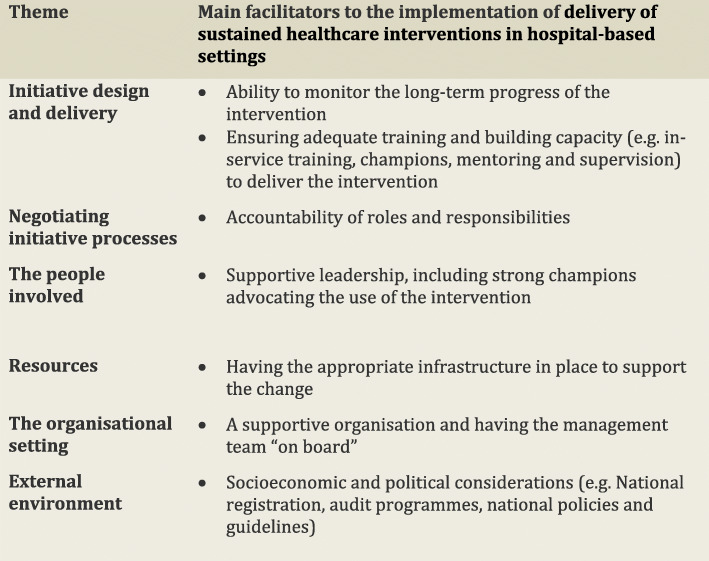
Key facilitators reported within each of the themes from the Lennox (2018) Consolidated Framework for Sustainability Constructs in Healthcare [12]

In the following subsections, we consider the volume of evidence reported by the included studies using a series of graphs (see Figs. 5, 6, 7, 8, 9 and 10) and key examples that were identified for each of the themes (see Additional files 9, 10, 11, 12, 13 and 14). Data categorised as ‘neutral’ was reported across 11 studies [31, 35, 39, 41, 43–45, 48, 49, 53, 59], represented less than 2% of all of the extracted data, and is summarised in Figs. 5, 6, 7, 8, 9 and 10. The main barriers and facilitators within each theme are summarised in Fig. 11 and Fig. 12 respectively.

#### Initiative design and delivery

##### Barriers

Fifteen studies reported barriers within this theme [31, 33, 34, 37, 39, 41, 42, 45, 49, 51, 54–56, 58, 61] (Fig. 5). The most commonly reported barrier was training on the intervention and capacity building [31, 33, 35, 37–39, 44, 45, 53, 56, 60]. Ament 2017 [31] emphasised the recurring nature of this barrier given that *“knowledge of and experience with the program is lost in the institution, as new doctors enter training every year”* (p1139, [31]).

Other studies described no formal training, or confusion surrounding training processes and expectations. Seven studies highlighted the lack of longitudinal monitoring to measure progress over time [35, 38, 42, 45, 56, 58, 60]. Challenges sustaining the intervention were also observed in six studies when staff were unaware of the impact of the intervention or the effectiveness of the intervention was not clearly demonstrated [31, 33, 41, 42, 54, 58].

Individual staff barriers including limited experience of delivering interventions, having a poor knowledge-base, and/or a lack of confidence were described in six studies [34, 42, 45, 49, 56, 61]. Other barriers included a lack of awareness of the problem [37, 39, 49, 51, 55]; the poor evidence-base underpinning the intervention [44] or projects viewed as time-limited, and linked to grant funding [33, 54]. Two studies reported that the lack of improvement methods, such as poor quality record keeping, were obstacles to sustained success of the intervention [33, 42].

##### Facilitators

Initiative design and delivery was identified as a facilitator for sustainability in all of the included studies (*n* = 32) (Table 1) (Fig. 5). The ability to monitor the sustained progress of the intervention [31, 32, 34, 37, 39, 40, 43–48, 51–54, 57, 60, 62], and ensuring adequate training and building capacity (e.g. in-service training, champions, mentoring and supervision) [33, 34, 36–39, 41, 43, 45–49, 51, 55, 60, 61] to deliver the intervention were the most frequently reported factors.

Fourteen studies stressed the value of having appropriate expertise and knowledge in order to deliver the intervention [31, 33, 34, 36, 44, 45, 47–50, 55–57, 60]. Eleven studies noted the importance of establishing the credibility of the intervention, ensuring a strong evidence base and being able to demonstrate evidence of effectiveness and benefit to patients and staff [31, 32, 34, 41, 44, 45, 51, 57, 59, 60, 62]. The severity and relevance of the problem that the intervention was targeting was also identified as a key facilitator in 9/32 studies [36, 38–40, 44, 48–50, 56]. The type of project [36, 46] and project duration [40, 42] were less frequently cited.

### Negotiating initiative processes

#### Barriers

Failure to negotiate initiative processes was identified as a key barrier in 24/32 studies [31–39, 41, 42, 44–46, 49–52, 54–56, 59–61]. Two main barriers were identified within this theme: workload pressures (*n* = 14/32) [31–34, 37–39, 44, 49, 50, 54–56, 59] and complexity of the problem being addressed (*n* = 14) [34, 36–39, 42, 44, 45, 49, 50, 54, 55, 60, 61]. Studies reported concerns that the interventions would increase the burden on staff already stretched by their current, often heavy, workload, with the intervention viewed as an additional task with little added benefit. The complexity of understanding, implementing and sustaining the intervention was also frequently reported. Role ambiguity and a lack of clarity around responsibilities involved in the intervention was also described as hindering sustainability in 13/32 studies [32, 36, 38, 41, 42, 45, 46, 50–52, 56, 59, 60]. The lack of staff confidence or belief in the intervention and whether it would improve current practice or patient outcomes was reported as a barrier in 9/32 studies [34, 37, 45, 49, 51, 54, 55, 59, 61]. Other challenges identified within this theme included confusion about how the intervention would work or be implemented (i.e. a lack of mutual shared vision) (*n* = 6/32 studies) [35, 42, 49, 54, 56, 59]. Other studies reported that specific job requirements were not clear and had not been agreed with staff or incorporated into their role [33, 45, 46, 52]. Studies also described no formal recognition or involvement as a disincentive [42, 46, 60].

#### Facilitators

Thirty studies reported that successfully negotiating initiative processes was a key facilitator [31–42, 44–60, 62]. Accountability of roles and responsibilities was the most frequently reported facilitator (*n* = 23/32) [31–34, 37, 38, 40, 41, 44–50, 52–56, 59, 60, 62].

Mazzacato 2012 [52], for example, stated that *“Before the hospital-initiated improvement efforts, different actors assumed their roles and responsibilities based on spheres of expertise … The lean intervention brought new roles and responsibilities (flow managers, team nurse and nurse’s aide, and team physician) which were further formalized in job descriptions. This contributed to reduce ambiguity and variation in how individuals carried out their work.”* [52] (p8–9).

Fifteen studies highlighted the importance of engagement with stakeholders and frontline staff, and including their perspectives to help define the objectives and shared vision of the intervention [32, 35–38, 45, 46, 52, 54–60]. Incentives including positive feedback, meeting targets for high quality care and certification were described as facilitative in 11/32 studies [31, 34–36, 38–42, 44, 46, 51, 53, 55, 58]. An equal number of studies also pointed out the importance of staff belief in the intervention as a positive contributor to sustainability [32, 34, 37–39, 48, 49, 51, 58–60]. Study authors reported that interventions were more sustainable when they reduced the complexity of an existing task by simplifying and streamlining processes [34, 36, 38, 41, 49, 54, 62]. As a result of the co-ordinated strategies, studies reported an improvement in job requirements [31, 32, 34, 44, 45, 57, 58] which enhanced team working or a reduced workload [34, 36, 52, 55].

### The people involved

#### Barriers

Sixteen studies identified the people involved as barriers to sustainability of the intervention [31, 33–35, 39–41, 45, 46, 49–52, 54, 55, 59]. A lack of strong leadership or support by change agents/champions was mentioned in 8/32 studies [31, 35, 41, 46, 49, 51, 54, 59]. The power distribution in relationships between professionals (inter-and -intra professional hierarchies) and power dynamics between professionals and patients were identified as a major barrier in 5/32 studies [34, 39, 45, 50, 55].

Failure to involve patients or consider their views and perspectives also negatively impacted on sustainability of the intervention [33, 45, 50, 52, 55]. Four studies pointed to the breakdown in relationships, collaborations and networks [39–41, 52] as a threat. Satisfaction [35, 52], community participation [33] and staff involvement [54] were least frequently cited as barriers across the studies. Stakeholder participation and ownership were not identified as barriers within this theme.

#### Facilitators

Thirty studies described the people involved as key to the success of the intervention [31–42, 44–60, 62]. Supportive leadership, including strong champions advocating the use of the intervention, was the most frequently reported facilitator in this category (*n* = 22/32) [32, 35, 36, 38–41, 44–47, 50–55, 57–60, 62]. This often included identifying “agents of change” (i.e.) staff who were committed to the intervention and who would take “ownership” [32, 44, 62] of the programme or intervention, helping embed the intervention into daily routine practice.

The distribution of “power” and the importance of engaging “all expertise in the team (the patient included)” [55] (p4) was also viewed as major factor underpinning sustainability in 15/32 studies [32, 33, 38, 39, 44–46, 48, 49, 51, 55, 58–60, 62]. Eleven studies [35–37, 39, 41, 45, 48, 51, 57, 59, 60] pointed to staff involvement from the inception of the intervention as key “to ensure acceptance and ownership of change in practice” (p11) [35].

Consideration of patient needs and satisfaction [31, 33–35, 45–49, 54, 55, 59] was reported as important, particularly when patients gave positive feedback on the intervention. Eleven studies also highlighted the importance of relationships, collaborations, partnerships and networks and their positive impact on sustainability [32, 34, 35, 38, 41, 45, 47, 49, 51, 56, 58].

### Resources

#### Barriers

Twenty two studies described resources as an obstacle to the sustainability of hospital-based interventions [31, 33–35, 37–44, 46, 49, 50, 52–56, 60, 61] (Fig. 8). Staff shortages, and /or a high turnover of staff were reported as the main barriers in 15/32 studies [31, 35, 37–39, 41–44, 46, 49, 50, 53, 55, 60]. For example, *“the turnover of newly graduated nurses as one barrier to success in the implementation project and sustainability of the new ‘routine’”* [50]*(p272).*

Other barriers included a lack of time, with staff being too busy or struggling to find time to implement the intervention in 8/32 studies [31, 35, 38, 39, 49, 53, 55, 61]. Seven studies pointed to poor infrastructure as negatively impacting on ability to sustain the intervention [33, 39, 44, 46, 52, 53, 56]. Other studies described delivery of the intervention (or components) as threatened, or abandoned altogether, if general resource issues were unavailable [35, 38, 39, 49, 54, 60]. Four studies described the challenge of securing long-term funding in the absence of any dedicated finance [33, 34, 39, 40].

#### Facilitators

Twenty three studies identified resources as an important facilitator [31–39, 45–47, 49, 50, 52–58, 61, 62]. Having the appropriate infrastructure in place to support the change, for example, a suitable work space or access to ‘good’ IT systems and software for documenting patient care, was the most frequently reported facilitator (17/32 studies [32–37, 46, 47, 49, 50, 52, 54, 56–58, 61, 62]). Other facilitators considered essential included appropriate staffing levels [31, 32, 37, 38, 46, 49, 55, 57, 58], and dedicated, protected time (staff or volunteer) [31, 32, 39, 46, 47, 49, 52, 62]. Adequate funding [34, 39, 45, 57, 58] and the availability of general resources [38, 46, 53] were critical, with one study arguing that *“national resourcing and regional support have undoubtedly boosted the rapid and widespread adoption and implementation of the programme”* [58] (p1205).

### The organisational setting

#### Barriers

Twenty four papers reported barriers in the organisational setting [31–35, 38–43, 45, 46, 48–51, 53–56, 58–60] (Fig. 9). Nine studies described the lack of support available as a major barrier [35, 38, 41, 42, 45, 46, 49, 51, 56]. Lack of organisational readiness and limited capacity to deliver the intervention was an obstacle to successful sustainability in 7/32 studies [42, 43, 45, 49, 54, 58, 59]. Failure to integrate the intervention within existing programmes and policies impeded sustainability in 7/32 studies [31, 33, 34, 42, 45, 53, 59]. Six studies reported barriers related to hospital culture and values [31, 38, 40, 45, 50, 54] including “changing long-held practices” [45] (p7). Conflicting objectives, competing priorities, or fatigue from previous implementation projects resulted in organisation opposition in six studies [39, 42, 45, 48, 55, 60]. Intervention adaptation and receptivity was the least frequently reported barrier [32].

#### Facilitators

Facilitators in the organisational setting were identified in 31/32 studies [31–49, 51–62]. The importance of a supportive organisation and having the management team “on board” was most frequently reported (*n* = 21/32) [32–35, 38, 40, 41, 44–49, 51, 52, 55, 57–59, 61, 62]. The ability and ease of the intervention to be embedded and integrated within existing services and policies was reported as a facilitator in sixteen studies [32, 33, 35, 36, 38–40, 45–49, 51, 52, 54, 57].

The capacity and organisational readiness to deliver the intervention was also highlighted as an important facilitator in 15/32 studies [32, 33, 35, 37, 41–45, 47, 48, 56–58, 62]. Glasgow 2013 stressed the importance of strengthening organisational strategies, in order to build a stronger ‘system’, and not solely rely on the number of staff available to deliver an intervention [43]. The ability of an intervention to be flexible or adjusted depending on local factors or contexts was key to sustainability in 13/32 studies [31, 32, 34, 38, 39, 41, 45–48, 54, 57, 60]. These studies also highlighted the longer-term value of being able to monitor and modify the intervention over time as local requirements changed. Twelve studies linked the sustained success of an intervention with the compatibility of the organisation’s beliefs, values and culture [31, 32, 34, 35, 37, 38, 41, 43, 51, 53, 54, 58].

### External environment

#### Barriers

The external environment was identified as a barrier in 4/32 studies [31, 32, 54, 56]. Three studies identified issues related to socioeconomic and political considerations [32, 54, 56]. For example, one reported that society had not kept pace with healthcare demands and highlighted multiple challenges (e.g. lack of sheltered homes, increasing ageing demographics) which impeded the sustainability of a discharge planning intervention [56]. A decreased awareness and a lowered profile, as a result of less intensive communications between hospitals in the post-implementation phase, was identified as a barrier to sustainability in one study [31] (Fig. 9, Additional file 13).

### Facilitators

Seventeen studies described the external environment as a facilitator of sustainability [31–38, 40, 44, 45, 48, 53, 54, 57, 58, 60] (Fig. 10). Socioeconomic and political considerations were most frequently reported (*n* = 9/32) [31–33, 36, 40, 44, 53, 54, 57]. National registration, audit programmes, national policies and guidelines which encouraged greater transparency and improved performance were identified as factors positively impacting on sustainability.

The ability of an effective intervention to spread to other sites – within and across organisations - was also considered important in 8/32 studies [31, 35–38, 48, 54, 58]. Five studies highlighted the value of media reports, publicity campaigns and marketing to raise the profile and improve public awareness [31, 34, 44, 45, 60]. Motivation (or urgency) to sustain an intervention was reported as a facilitator in one study [54] (Fig. 10, Additional file 14).

### Other factors

Our review identified other barriers and facilitators reported in 14/32 studies [31, 33, 35, 39, 41–43, 45–49, 54, 55] that we could not map to the predefined constructs [12]. We organised the data that we could not map into two new constructs to refine the Lennox framework for hospital settings and two linked principles about sustainability in hospitals that complement it.

The first new construct is the spread *from* other sources including other parts of an organisation [33, 42] (External environment theme) is a key influence for sustainability. This could include educational influences, for example from invited speakers (e.g. 44) or a site visit to observe practice (53). While Lennox (2018) include a construct of Spread *to* other organisations, as observed by Frykman et al. (2017) *“High interdependencies with organizational processes outside of the ED / the ED was part of a complex system in which ED work processes and ED staff changes at the hospital level affected the conditions for teamwork at the ED” (p73)* [42]*.*

The second proposed new construct is initiative development, to acknowledge explicitly that sustainability may require adding to or developing the intervention (37, 57). The data pointed to the need to accurately capture the adaptations that occurred to the intervention over time in order to fully understand sustainability [31]. Adaptation of the organisational context is catered for within the Lennox (2018) framework (Intervention adaptation and receptivity), however, it does not specifically cover adding to the initiative over time, for example *“Some respondents also felt that program champions were necessary to …be able to add to the program”* [39].

The first principle to complement the Consolidated Framework is the extent to which sustainability is unpredictable in hospital settings (refs [32, 41, 46, 47, 62]). One study reported that “Across sites, there was no discernible pattern in the proportion of barriers and enablers identified or the nature of barriers and enablers related to sustainability considerations, such as culture, capacity, supportive environment, or clinical features” (p.10) (ref [32]), while another found that *“knowing performance and organizational details for one hospital would not aid in predicting performance at another hospital”* (p199, [43]).

A linked principle to accompany the Consolidated Framework is that approaches to sustainability need to be multifaceted (refs [47, 50, 54, 64]). Studies described the importance of the combination and interaction of measures to sustain long-term change [41]. For example, Bhanbhro et al. (2016) commented that “*there was no single measure that sustains long-term change in practice for NHS rehabilitation units. Rather, that several interconnected measures need to be considered prior, during and after a new programme is introduced.”* (p12) [35].

## Discussion

### Summary of the main results

Our systematic review identified 32 empirical studies where theoretical frameworks were explicitly used to address sustainability of hospital-based interventions. Of these, 72% of studies employed a qualitative design, and 50% were conducted in the UK or USA. Most of the studies (84%) involved a combination of participants from frontline and higher organisational levels (unit, ward, department).

All of the hospital-based interventions were multicomponent and delivered in diverse healthcare settings, with most of them aimed at improving patient care and/or safety. Interventions were frequently reported as tailored (25/32) and/or modified (14/32) but details about how and when they were changed were poorly described. Only two studies (6%) reported all sustainability constructs as defined by Moore (2017) [8]. The most frequently reported sustainability contruct (88%) was the length of time interventions were sustained which ranged widely across studies, from 6 months – 8 years.

Our review documented a variety of theories, frameworks and models, with almost half of the studies (15/32) examining sustainability by drawing on established implementation frameworks (e.g. NPT [36, 38, 44, 55, 56], CFIR [31, 46, 53, 60]). Further coding of frameworks reported in the included studies using Nilsen’s taxonomy of theories, models and frameworks [29] showed that 13/32 studies had used a determinant framework to provide better understanding and explanation of how and why sustainability succeeds or fails. Determinant frameworks have been argued to provide a generic structure to the implementation process, but have also received criticism as their stucture lacks sufficient detail to translate into meaningful guidance that can be tailored to the specifics of individual ward contexts [29]. In addition, determinant frameworks do not address the dynamic process elements of sustainability, but rather frame implementation as a one-off event [29]. The Consolidated Framework for Sustainability Constructs in Healthcare is a determinant framework; although in looking for best fit with existing constructs we found it to be comprehensive, this may explain why the data we could not code to it all refers to sustainability as a dynamic process.

Barriers and facilitators were reported in all studies. However, we were surprised to identify twice as many facilitators reported in the literature compared with barriers. When mapped to the Consolidated Framework for Sustainability Constructs in Healthcare, three constructs were reported as key influencers in sustaining change in practice in hospital settings: clear accountability of roles and responsibilities while negotiating initiative ​processes (72%); ensuring the availability of strong leadership and champions advocating the use of the intervention (69%), and provision of adequate support available at an organisational level (66%). The high prevalence of these three facilitating factors across all studies provides a helpful indication of aspects that should be addressed to help support sustaining an intervention over time. This combination of ward level champions who have clear responsibilities for facilitating ongoing implementation along with strong support provided at an organisational level is regarded as hugely impactful in achieving sustained intervention use.

The most frequently reported barrier to sustainability in almost half of the included studies was inadequate staff resourcing (15/32 studies, 49%) usually as a result of staff shortages, and /or a high turnover of staff. This finding reflects those of implementation studies where issues around staff resourcing also feature strongly as a barrier. It is important that capacity in terms of ‘how much’ a ward can do is addressed to ensure that staff are given the time and space to engage with interventions. If staffing levels are low and/or in constant fluctuation it is clear why sustaining an intervention cannot be given the time required. Organisations need to ensure appropriate investment in infrastructure to ensure staff can have sufficient time in their role to engage with improvement work, not just at point of delivery but on a daily basis, such that interventions become embedded in practice. The fact that the most frequently reported barrier was only discussed in less than half of the studies reveals a large variability in what can potentially impact on sustainability. This finding reiterates the importance of addressing context and recognising factors specific to a given organisation/setting that could potentially impact on sustained intervention use.

Parallel to our review of sustainability in hospitals, Penno et al. (2019) [70] have made a welcome contribution to this emerging field by conducting a systematic review and theory analysis to understand sustainability specifically in acute settings. Like us, they selected Moore et al’s [8] definition of sustainability and chose to draw on Lennox et al’s [12] consolidated framework, suggesting that these may become index papers in the field. Penno et al. (2019) [70] compared eight frameworks designed specifically to support sustainability of evidence based practices in healthcare settings; interestingly only three of these featured in the 32 papers in our sample, which likely reflects our more inclusive approach towards authors’ choice of frameworks. Different methods and interpretation of the Lennox constructs limit the extent to which our findings can be directly compared. Penno et al’s review reinforces our finding of the centrality of strong leadership to sustainability, and the need to regard it as a process. It did not identify our key combination of facilitators or proposed new constructs, which again may be a consequence of differences in inclusion criteria.

The review of implementation in hospitals by Geerligs et al. is also an opportunity to add to previous findings that sustainability is a distinct phase [1]. Geerligs et al. identified key interacting domains of the system, staff and intervention. Although again not directly comparable due to different methods, the three most frequent facilitators for sustainability across papers included in our review were accountability of roles and responsibilities, strong leadership and champions advocating the use of the intervention, and adequate organisational support. Roles has synergy with Geerligs et al’s staff domain, which focuses on staff beliefs, engagement and skills. Organisational support aligns with their system domain, which highlights the importance of understanding the organisational context. Our third most prevalent facilitator – leadership – suggests that this may assume more importance for the sustainability phase than the intervention itself.

Our work builds on the comprehensive framework published by Lennox and colleagues [12], by identifying two new constructs and two new linked principles applicable to hospital settings. These were the spread from other sources initiative development; the (un) predictability of sustainability; and that sustainability plans need to be multifaceted. We see these emergent constructs and principles as also augmenting the work of Chambers et al. (2013, [13]) because they provide tangible elements that enable use of a theoretical framework as a practical tool. This has potential for supporting staff to ensure interventions are used and sustained in the work place.

In common with the Geerlig’s et al. review of implementation in hospitals [1], and Penno et al’s [70] review of sustainability frameworks for acute settings, we have demonstrated that any attempt to support sustainability must work with its dynamic nature. We would advocate for continued research exploring the dynamic nature of sustained implementation, trying to better understand this evolutionary process and the role of frameworks in identify fluctuating factors that influence sustained success and ways in which they can dynamically be addressed. In addition, both our reporting of barriers and facilitators and our emergent constructs and principles highlight that influencers on sustainability need to be considered in a reciprocal manner. In other words, the interplay between factors and the compensatory manner in which a facilitator of sustainability can outweigh a potential barrier to sustainability needs to be addressed. For example, a lack of adequate resourcing may not impact on sustainability if there is sufficient presence of facilitating factors on the ward (e.g. an enthusiastic, motivated workforce). It is important therefore to review both potential barrier and facilitator factors as a whole, assessing each factor’s level of influence and relative influence on the likelihood of an intervention being sustained.

Our findings also highlight the need for ongoing assessment of the readiness of a ward in terms of its ability to support sustained intervention use. The importance of planning and contingency planning cannot be overstated in ensuring that an intervention is being delivered in the least hostile environment possible. This is an aspect that should be addressed pre-intervention but also continuously over time [13]. Implementation and sustainability are dynamic processes, not one-off occurrences, and need to be recognised as such through the provision of continuous resources and processes to support them.

### Strengths and limitations

This systematic review was performed with a high level of rigour as outlined in our protocol paper [17]. We sought studies where the authors had an empirical interest in sustainability of interventions in a hospital setting, which they structured explicitly through use of a framework. Apart from this commonality, the 32 included studies were heterogeneous in every respect. For example, sustainability was examined through an implementation science lens, an improvement science lens, and a sustainability lens. We integrated them through extracting reported barriers and facilitators to sustainability and mapping these to an existing consolidated framework. While this risks a charge of comparing apples with pears, hospital staff face multiple competing demands for sustainability, which is reflected in the included papers and our methods.

It is possible despite our best efforts that we may not have identified all of the relevant literature. In part, this may be due to the breadth of the original research question, and magnitude of the literature returned from our systematic searching. Furthermore the literature in this area is dogged by inconsistent definitions (e.g sustainability) and/or little or no methodological detail regarding frameworks reported within abstracts to warrant inclusion in the final studies reviewed.

We only included studies published in English and studies included were limited to peer-reviewed literature only. Consequently there is likely to be a large body of unpublished literature or publications in other languages relating to sustainability, and it is possible that other facilitators and barriers would have been identified if such literature had been included. In addition, the review was conducted in December 2017, so literature published after this date was not included. However, as the research included here is largely qualitative, any further research would be unlikely to alter the findings significantly [71, 72].

Both when reporting and exploring results relating to barriers and facilitators we have focused on data relating to the frequency of reporting across the included studies. This is a major limitation of our study, as it focuses solely on the number of reports. Frequency of reporting of a barrier or facilitator should not be interpreted as a measure of the size or extent of the problem relating to that influencer. Despite the limitations of our approach, it enabled a focus on barriers and facilitators which may be amongst the most frequent in clinical practice. The heterogeneity of study designs, and large proportion of studies with qualitative data only, mean that there are currently few alternatives to quantifying main issues in this field.

Despite acknowledged limitations, this review provides the first comprehensive overview to our knowledge of the evidence around how to support sustained use of interventions in hospital settings. It therefore has potential practical impact for both policy makers and the implementation research community.

### Implications for practice

Three factors should be proritised when delivering any innnovation in a ward setting: ensuring there is stong senior organiational leadership to support the innovation; having ward level champions to promote the innovation; and having clear accountability of roles and responsibilities for delivery of the innovation. Many innovations require staff to do more, and even doing the same things differently can take more time. It is important to ensure that sufficient resource is continually available to support the delivery of the planned innovation if it is to be sustained over time. It is also important to consider the local context and, when introducing a new innovation, consider that what has worked well in one location may not automatically work as well in a different setting. Ward situations continually change, so it is important to routinely monitor the factors that support or inhibit innovation delivery.

It should be noted that despite these clear recommendations for practice, we recognise the majority of included studies were conducted in developed countries. Potentially this may limit the generalisability of our results to low and middle income countries where health systems can be very different from those in developed nations.

### Implications for research

To achieve increased understanding of how to facilitate sustained use of interventions, it is important that studies reported in the literature adopt a more consistent and complete approach to reporting of sustainability and provide specifics of what happens to an intervention over time. Many studies are limited by the funding available, therefore, we would also argue that funding bodies need to support long-term evaluation work to allow the evidence base around sustainability to grow.

The scope of our review was limited to hospital in-patient settings. The narrow focus of the review allows for findings to be of practical benefit to this setting and ensure results are not too generalised as to be unbeneficial. Recommendations for future research include extending the scope to other areas, such as primary care, out-patient facilities and ambulatory care. It would be interesting to compare factors that impact on sustainability in such settings to identify if context plays a role in influencing the main facilitators and barriers to sustainability.

Factors which impact on implementation are not necessariliy the same factors that impact on sustainability. Similarly, it cannot be assumed that the factors influencing intervention sustainability remain constant. As highlighted in this review, sustainabilty is very much a process, and as such can be influenced by different contextual or mechanistic factors at different times. Frameworks to support sustainability must therefore support this changeable landscape, capturing the different patterns of sustainability in order to help support continued intervention use.

## Conclusions

Our review provides a contemporary insight into the use of frameworks for sustaining interventions in hospitals. Key components for sustaining an intervention have been identified and inform what aspects any sustainability frameworks need to address if they are to have a positive impact on practice and patient care. In particular, we reflect on the dynamic nature of implementation, and promote the need for a framework to address implementation not as a one-off event, but a continuous, evolving process.

This review is a valuable addition to the evidence base around the use of frameworks to support sustainability. It has also highlighted the need for more consistent and complete reporting of sustainability if we are to learn best practice for implementation work going forward.

## Supplementary information

**Additional file 1.** Search string example.

**Additional file 2.** Selection criteria.

**Additional file 3.** Summary of methods used in the included studies.

**Additional file 4.** Summary of the interventions or programmes delivered.

**Additional file 5.** Methodological quality assessment for qualitative studies.

**Additional file 6.** Methodological quality assessment for mixed method design studies.

**Additional file 7.** Methodological quality assessment for quality improvement studies.

**Additional file 8.** Table of included studies mapped to Moore (2017) definition of sustainability.

**Additional file 9.** Key examples of barriers and facillitators within Initiative design and delivery.

**Additional file 10.** Key examples of barriers and facilitators identified within the Negotiating initiative processes theme.

**Additional file 11.** Key examples of barriers and facilitators identified within the people involved theme.

**Additional file 12.** Key examples of barriers and facilitators identified within the Resources theme.

**Additional file 13.** Key examples of barriers and facilitators identified within the organisational setting theme.

**Additional file 14.** Key examples of barriers and facilitators identified within the external environment theme.

**Additional file 15.**

## Data Availability

All data analysed or produced as a result of this review are included in the main file and Additional files.

## Abbreviations

CFIR: Consolidated Framework for Implementation Research
MRC: Medical Research Council
NPT: Normalisation Process Theory
PARiHS: Promoting Action on Research Implementation in Health Services
PRISMA: Preferred Reporting Items for Systematic review and Meta-Analysis
PRISMA-P: Preferred Reporting Items for Systematic review and Meta-Analysis Protocol
QUERI: Quality Enhancement Research Initiative
RE-AIM: Reach, Effectiveness, Adoption, Implementation, and Maintenance
TIDieR: Template for Intervention Description and Replication
