# Supplementary material for: The barriers and facilitators influencing the sustainability of hospital-based interventions: a systematic review
Source: BMC Health Serv Res. 2020 Jun 28;20:588. doi: 10.1186/s12913-020-05434-9 (PMC7321537; doi:10.1186/s12913-020-05434-9)
Supplement: Supplementary file 1 — Additional file 1. Search string example. [file 12913_2020_5434_MOESM1_ESM.docx]

**ADDITIONAL FILE 1. SEARCH STRATEGY AS DESIGNED FOR MEDLINE (Ovid) AND ADAPTED FOR THE OTHER DATABASES**

**Medline(R) ALL (Ovid) from 2008 to 18 December 2017**

1. (theor$ or model or models or principle$ or construct$ or framework$).mp
2. exp Patient care/
3. exp Patient admission/
4. exp Inpatients/
5. exp Hospitals/
6. (hospital$ or inpatient$ or in-patient$ or ward$ or unit$).mp
7. or/2-6
8. (sustain$ or implement$ or long-term implement$ or long term implement$ or routini?ation or discontinue$ or de-adoption or deadoption or durabil$ or institutionali?ation or maintenance or capacity building or knowledge utili?ation).mp
9. 1 and 7 and 8
10. limit 9 to (English language and year=“2008-Current”)

Note: [mp=title, original title, abstract, name of substance word, subject heading word, unique identifier]
