# Supplementary material for: The barriers and facilitators influencing the sustainability of hospital-based interventions: a systematic review
Source: BMC Health Serv Res. 2020 Jun 28;20:588. doi: 10.1186/s12913-020-05434-9 (PMC7321537; doi:10.1186/s12913-020-05434-9)
Supplement: Supplementary file 2 — Additional file 2. Selection criteria. [file 12913_2020_5434_MOESM2_ESM.docx]

**ADDITIONAL FILE 2. SELECTION CRITERIA**

| **Inclusion criteria** | **Exclusion criteria** |
| --- | --- |
| Peer reviewed publications  Language limitation: English  Date limitation: 1 January 2008 – Dec 2017 | Studies that are not published in English or are not peer-reviewed publications.  We will not include any non-research study designs (e.g., unstructured reviews or overviews, theoretical papers, commentaries or opinion papers, protocol, case study, editorial, audit, letter). |
| Empirical studies relating to sustainability of an intervention or programme conducted in a hospital setting. | Studies that are not conducted in hospital-based settings. In the case of studies performed across multiple settings, studies will be excluded where results pertaining to the hospital setting are not clearly identifiable. In addition, if the service provided is regarded as an outpatient clinic, then the study will also be excluded. |
| Studies should focus on sustainability intended to improve patient care and must incorporate a framework or theory or model. | We will not include any studies that do not discuss a specific intervention or programme (ie, solely reports programmes at a general systems level) or only discusses sustainability prospectively (i.e, an empirical study has not been carried out). Finally, we will not include studies where sustainability is not a specific concern of the study (i.e, concerned only with adoption and initial implementation of the intervention/ programme) and does not make any reference to frameworks, theories or models that relate to sustainability. |
