# Supplementary material for: The barriers and facilitators influencing the sustainability of hospital-based interventions: a systematic review
Source: BMC Health Serv Res. 2020 Jun 28;20:588. doi: 10.1186/s12913-020-05434-9 (PMC7321537; doi:10.1186/s12913-020-05434-9)
Supplement: Supplementary file 3 — Additional file 3. Summary of methods used in the included studies. [file 12913_2020_5434_MOESM3_ESM.docx]

**Additional File 3. Summary of methods used in the included studies**

**Key**: ERAS: Enhanced recovery after surgery; NR: not reported, SSP: short stay program; SSI: Semi-structured interviews; MDT: Multi-disciplinary team; B/F: Barriers/Facilitators; MM: mixed-methods; QCS: Qualitative Case Study; RA: Realist Approach; CMO: Context- Mechanisms-Outcome; PO: Purposive sample; RCT: Randomised Controlled Trial; NPT: Normalisation Process Theory; RE: Realist Evaluation

| **Study**  1. First author  2. Year ^(ref)^ | **Method** | **Sampling** | **Reported time period** | **How is sustainability measured** |
| --- | --- | --- | --- | --- |
| 1. Ament  2. 2017 (1) | 21 SSIs (26 respondents). Interview guide based on CFIR. Interview focus: reasons why outcomes and program elements were maintained after initial successful implementation. | Hospital sampling: The primary implementation strategy must have shown early postimplementation effectiveness. Respondents: key members of the current MDTs of ERAS and SSP. Surgeons at each location were invited but if not able, the interview was held with a nurse practitioner. Additional respondents were collected via snowballing. | 3-6 years following implementation. ERAS- implementation:2005-09. SSP implementatio: 2005-07. Current study: March 2012- May 2013. | Content analysis perfomed. Data collection and analysis were performed simultaneously. First 5 interviews analsyed followed by subsequent interviews using deductive thematic coding approach for each of the 2 implementation cases, using the CFIR as a coding scheme. After codes were assigned to CFIR domains, an inductive coding was used at the construct level to explore themes not covered by CFIR but considered relevant. |
| 1. Belizan  2. 2011 (2) | Qualitative approach: data collection workshops "devoted to implementation and maintenance of audit programmes". | Sites that reached the stage of sustaining the audit programme took part in the workshops. Unclear how they sample individual participants. | For PIPP >= 5 years, Child POP >=3-4 years. | Participants divided according to the level of care (district hospital, regional and a mix of regional and provinvial hospital) for workshop session 1 and then mixed for session 2. Began with grounded theory approach, then used stages-of-change conceptual framework. |
| 1. Bergh (3)  2. 2014 | Across 3 countries, stakeholders attended a national plenary meeting. In Rwanda: qualitative interviews. 11-13 delegates per country. Focus: history/status of KMC, role of stakeholders in implementation.  Standaritised key informant interview q’nnaires and observation inventory used to measure types of practices and aspects of services. Local assessors trained to use q’nnaire and conduct interviews with focal people and staff at each facility and observed KMC practices. Feedback and written report were provided to each facility. | Countries: 4 countries purposively selected (Malawi, Mali, Rwanda, Uganda). Key informants: stakeholders selected representing higher level structures (e.g. government, program developers and coordinators) and health facilities representing grass root structures. A convenience sample to represent all levels of care and different geographical location was chosen for the health facilities. | Unclear | Each assessor completed their q’nnaire and discrepancies resolved through discussion. Descriptive statistics conducted based on 6 stages of change (a total score of 30). creating awareness (2 points); adopting the concept (2 points); taking ownership (mobilizing resources) (6 points); evidence of practice (7 points); evidence of routine and integration (7 points); sustainable practice (6 points). Results from analysis and facility q’nnaires were compared with the themes recorded in the stakeholder meetings and interviews tp determine development of KMC implementation and B/F. The final interpretation was confirmed by key role players in each country. |
| 1. Bernstein  2. 2009 (4) | MM. Quantitative descriptive statistics to measure implementation (services provided): negative and positive screens. Qualitative semi-structured interviews and a yearly progress report. 24 interviews conducted in sites that showed sustainability. | Hospitals: process of applications and proposals. | 18 months | Interviews were analysed according to the RE-AIM elements. |
| 1. Bhanbhro  2. 2016 (5) | QCS analysis using a blend of traditional ‘framework’ analysis and RA. Multiple sources of existing data collected during the REAL programme: transcripts of staff focus groups (3) and service user interviews (4); getREAL team members' daily reflective practice dairies (26), unit staff evaluation forms (9), fidelity monitoring sheets (3), supervisor notes (6). Data analysed using RA and emerging themes. Identified programme theories to be tested, which were articulated in the form of CMO configurations. The data were interrogated by the identified candidate theories to see if they could explain the complex footprint of outcomes left by the intervention. | PS to achieve multiplicity of unit chracteristics rather than representatieveness. Unit selection criteria: took part in cluster RCT and received GetREAL intervention; The unit took part in a staff focus group (2-9 months post intervention (nine of the 19 units that received the GetREAL intervention participated in staff focus groups)); The unit had high, mid or low scores in the trial’s primary outcome measure, service user activity as assessed using the Time Use Diary (TUD) at 12 month follow-up; The unit had a complete dataset containing GetREAL team reflective diaries, staff focus group  and service user interview transcripts, unit action plan and fidelity sheet. | NR | Data were interrogated by candidate theories to see if they could explain the complex footprint of outcomes left by the intervention. They have described B/F using CMO. |
| 1. Bouamrane and Mair  2. 2014 (6) | 3 SSIs with stakeholders, 1 case study at a preoperative clinic in an NHS GGC Acute Care Hospital (ACH), a focus group with team members of the eform, 2 forums with members of ehealth programmes across health boards. | Interviews: Contacts made with NHS GGC via email to identify key individuals behind the development and implementation of the electronic preoperative  clinical portal. Three main stakeholders were identified and contacted by email. | The e-form was rolled out in 2009 and interviews took place in 2011 | Data analysis used NPT to map factors that contributed to the deployment of the eform |
| 1. Brady  2. 2014 (7) | Survey – baseline and post-interactive education sessions held to assess knowledge and practice behaviour intentions for the medical treatment of a patient with acute haematogenous osteomyelitis. Clinical vignette. Survey was completed by hospital medicine faculty members, residents and medical students.  Observational time series study was conducted using medical record audits. Data analysis: Wilcoxon signed rank test for survey results, Fisher's exact test for frequency of infections on orthopaedic consultations, a run chart of discharged patients rates, counts for post discharge outcomes, t-tests for charge data. | Unclear | Practice change was sustained for > 12 months | Surveys and comparing pre-and-post implementation results |
| 1. Bridges  2. 2017 (8) | Qualitative process evaluation, SSIs at different time points (3-6 and 7-12 months), observations of intervention activities (2 study days and 5 action-learning sets), staffing data through q’nnaires. Constant comparative method of data analysis, not aligned with an existing qualitative data analysis approach | PS to capture variations in staff grade and ward | Over a 12 month period; of which 4 months were intervention implementation | Through NPT from data collection to data analysis |
| 1. Campbell  2. 2011 (9) | Telephone SSIs with SCC and DM. Focus on implementation factors, organizational setting, hospital reactions to OMSC and perceived sustainability of the program. | Hospital units selected based on staff interest, ability to redeploy resources and patient smoking rates. Information mailed to each hospital SSC who was asked to identify the hospital DM. | Unclear | Combined data from DM and SSC and used open coding. Data then linked to Gruen et al.'s model (2008) (DOI: [10.1016/S0140-6736(08)61659-1](https://doi.org/10.1016/S0140-6736(08)61659-1)) |
| 1. Fleiszer  2. 2015 (10) | Framework guided SSIs, document analysis and observations | E-mailed invitation letters to nurses who have participated in the program. Unclear how they obtained documents or attended conferences and meetings. | 8 years after program initiation | Qualitative content analysis- deductive and inductive coding based on the framework |
| 1. Fleiszer  2. 2016 (11) | Embedded, multiple, comparative case study - comparing units with high/low sustainability. Interviews, site visits, program-related documents. Interview questions were initially open. More specific questions were posed if informants did not address main components of the framework, or to generate details about salient topics. Qualitative content analysis with a combination of deductive and inductive data | Units: Nominations for ‘‘high’’ and ‘‘low’’ sustainability units obtained from organizational informants in  first part of the study. Additional inclusion criteria were that units: had implemented at least two guidelines, more than two years prior; were not specialized; did not have idiosyncratic circumstances; and were not participating in other major studies, narrowing the pool to five ‘‘high’’ and four ‘‘low.’’ Then reviewed unit-specific patient outcome data collected by the organization since program inception, to ensure that the nominations of high/low sustainability units were consistent with the outcome trends. Participants: PS informants from the four units, seeking variety in job position and involvement in program implementation. | 7-8 years | Analyses considered participants’ perspectives on the importance of, frequency of reference to, and detail of description about issues related to program sustainability. Documents and observations were used as secondary sources of information about organizational/unit contexts. |
| 1. Frykman  2. 2017 (12) | Non-participant observations, interviews (individual and group). RE with phases 1 and 2: generation of a program theory and development of hypotheses as CMO configurations; and phases 3 and 4: testing of the CMO configurations and refinement of the program theory. | Staff members from all professional groups who had the experience of working at the ED both before and after the implementation of teamwork. | 2 years since end of implementation period | Outcome: sustainability of teamwork behaviours. Observations to assess sustainability of teamwork behaviours measured as the frequency of teamwork behaviors. Observations focused on the key aspects of sustainability: coordinate work, communicate decision to change plan, assemble when tasks have been performed, work in parallel, and communicate the work plan.Interviewsto develop and test the CIMO configurations used to explain the sustainability of teamwork.Interview transcripts analysed to identify changes in context, intervention and mechanisms. Existing observation data from a previous study in 2011 was used as a baseline for evaluation. |
| 1. Glasgow  2. 2013 (13) | Two surveys were conducted. Survey 1 measuring organisational characteristics. Survey 2 measuring clinical practice characteristics that may be associated with quality of care and high performance. Decision tree models. Data mining. | Use available data from hospitals that took part in a bigger study | NR | Data mining |
| 1. Gould  2. 2016 (14) | Retrospective evaluation. Qualitative interviews, open ended questions exploring evidence of change and B/Fs to promoting ownership. Data were analysed inductively. Routine data on c difficille rates | Maximum variability sampling to include a variety of types of staff in terms of occupational group and seniority | 18 months | Infection rates and mapping factors onto NPT constructs |
| 1. Gramlich  2. 2017 (15) | 1 survey (provider level), meeting notes, 3 learning collaboratives (patient, provider and system level), 2 focus groups (patient, provider and system level), 4 interviews (patient, provider and system level), status reports, and memos (patient, provider and system level). Table 1 does not match what is says in the text about the number of focus groups, interviews etc. All the qualitative data were aggregated for data analysis. | Unclear | Data were analysed after all six participating sites had been in implementation for at least 32 months. | Applied QUERI considerations articulated by Stetler et al. 2011 (DOI: 10.1186/1748-5908-6-99) |
| 1. Green  2. 2017 (16) | Retrospective analysis of data. Deductive coding. | Unclear | 6, 12 and 18 months | By mapping onto CFIR during deductive coding of the data. |
| 1. Hommel  2. 2017 (17) | Qualitative descriptive design. Individual interviews and focus groups | Criteria for hospitals: (1) hospitals with changes of the prevalence of PU categories II–IV between 2011–2014 (PU category I excluded as they can be difficult to detect), (2) number of participating patients in the prevalence study (Uni. hospitals > 300 patients, central hospitals > 100 patients and local hospitals > 50 patients and (3) the percentages of patients at risk for PU remained unchanged for the hospitals during 2011– 2014. Criteria for respondents are unclear. | 3 years. Programme was implemented in 2011 and the inclusion criterion was the PU rates have come down between 2011 and 2014 and interviews/focus groups were done in 2014 | Not explicitly reported |
| 1. Hovlid  2. 2012 (18) | Explorative QCS grounded in a theoretical framework. Interviews with key informants (open ended questions about identification of a need to change,  planning the change, actions taken to induce change, outcomes of change, and adaptations of interventions). Data were analysed according to Cresswell (Cresswell, J.W., 2002) | PS with informants of different roles in the hospital | Authors report that changes were sustained for 2 years. The timeframe for the interviews is given but not for the programme | Not explicitly stated but used theory to design the interviews and analyse data in order to explore factors that affect sustainability |
| 1. Ilott  2. 2016 (19) | Prospective longitudinal design. SSIs (group/individual) and document reviews. Interviews covered understanding of the dysphagia recommendations, experience of the interventions and examples of change in clinical practice. Documents included a field journal, education and training policies, essential skills logbook. Analysis: a framework approach. Data brought together from the outset using the small theory they created. | Purposive based on roles in the organisation | 34 months | Mapped onto a small theory developed from previous theories |
| 1. Jangland and Gunningberg  2. 2017 (20) | Quantitative data collected using the questionnaire ‘Quality from the Patient’s Perspective’. Analysis: descriptive statistics. Interviews: nurses, topic: patient participation. Analysed using directed content analysis; focused on the nurse managers’ perspective on patient participation and the learning process of the project and sorted data based on PARiHS | Consecutive sample of patients admitted from surgical waiting list/ED included in the survey. Inclusion criteria: adult patients (≥18 years) with a hospital stay of  at least 1 day. The nurse managers (n = 5), one from each unit, were invited to participate in individual interviews. | Intervention lasted 2 years (2011-2013). Data were collected in 2014 | PARiHS and identified barriers to sustainability based on PARiHS components |
| 1. Matthew-Maich  2. 2013 (21) | 64 in-depth interviews with health professionals, 56 phone interviews with new mothers. | PS of acute care hospital sites based on: (1) implemented the RNAO Breastfeeding BPG (RNAO 2003; 2007) in last 2 years with min. of 6 months since initial implementation; (2) intentional implementation process used; (3) site leaders willing to support research process; and (4) hospital offered variation from other sites. Purposive, criterion-based, and maximum variation sampling strategies were used with initial interviews, followed by theoretical sampling. Snowball sampling used to identify information-rich professionals (Patton 2002). Maximum variation sampling used for health professionals. All participants had to be English speaking. Inclusion criteria for mothers were adapted from a previousBPGevaluation (Edwards et al. 2003): singleton, term, healthy infant birth, discharged home together after min of 24-hours hospital stay, no documented use of street drugs or majormental illness and not a midwife birth. | 6-24 months | Separated sites into uptake and minimal uptake with uptake sites showing consistent use of the breasfeeding BPG in practice. Used grounded theory. |
| 1. Mazzocato  2. 2012 (22) | Mixed-methods explanatory single case study. Quantitative aspect: hospital’s weekly averages data of a) proportion of patients leaving A&E <=4 hours, b) waiting time from triage to first A&E physician consultation, and c) patient volume. Qualitative aspect: data on the intervention planning phase were collected retrospectively (interviews and documents). The implementation phase was studied prospectively (observation, interviews, documents); non-participant observation. Interviews focused on individuals’ work tasks; the way individuals’ work related to that of others; factors hindering effective delivery of care; intended and actual process hanges; and expected and measured outcomes. Analysis: ANOVAs, statistical process control charts, case description and case analysis. | NR | 2 years | Examined significant differences in outcome measures |
| 1. McClung  2. 2017 (23) | Phenomenologic qualitative study of interviews with key informant health care workers. Individual face to face interviews SSIs. | Snowballing sampling to recruit respondents involved in the implementation of HAI prevention, via email. | CLABSI bundle - 4 years, CAUTI bundle 1 year, CDI bundle 10 months | Used a thematic approach based on the CFIR model |
| 1. Mitchell  2. 2017 (24) | Interviews, focus groups, site visits. Little information given. Data analysis follows a modified grounded theory approach with constant comparative analysis | All 5 hospitals that implented RED were visited. Unclear how staff were sampled. | Implementation started in 2012 and 2013, interviews 2 years later; although each hospital has 2 years funding to implement RED. Based on their definition of sustainability the timeframe is 6 months but it doesn't seem to apply to all sites. | Not clear. Authors report asking about implementation in the interviews, but the reported timeframe for sustainability is unclear across each site. Sustainability after funding has been withdrawn is discussed. |
| 1.Naldemirci  2. 2017 (25) | SSIs with researchers and HCPs | Researchers: PS in different intervention studies, selected to represent different contexts of PCC. HCPs: PS. Each ward manager contacted with information about the study and gave their consent, recuit a nurse, an assistant nurse and a physician with experience of working with PCC. | Not reported | Intervention designed to address sustainability. Strategies mapped onto NPT components during data analysis. Performed inductively and then mapped onto NPT. |
| 1. Nordmark  2. 2016 (26) | Qualitative study: data from workshops, adverse events, system failures, online surveys, interviews. The various text units in each core area were compared and discussed during data analysis | Surveys: All RNs from the central county hospital invited to participate. Those who had a temporary position and/or night-only workers excluded. Interviews: RNs working at 5 different hospital wards. | Discharge planning implemented in 1992 and in place for 12 years. Study was part of a project conducted between 2009-12. | Used NPT as a framework during data analysis to explore embedding and integration. |
| 1. Parand  2. 2012 (27) | Qualitative repeated samples research; SSIs | PS with coordinators at each hospitals | At the end of the programme and a year later | Direct questions around sustainability asked in the SSIs. Then data analysed using content analysis and constant comparative technique |
| 1. Robert  2. 2011 (28) | Routinely collected data: data related to the adoption of PW (when the PW package was donwloaded and when the support package was purchased). National online survey: explore perceptions of the PW in terms of the components of the diffusion of innovation model. Organisational case studies: semi structured interviews on implementation, outcomes and sustainability. | Online survey: targeted at staff in NHS acute hospitals which had implemented or were considering implementing PW. Case studies: sites were selected on the basis of regional distribution, stage of implementation, type of support package purchased and willingness to participate. Staff for interviews were nominated by PW leads | Not clear | Assimilation. Interpretative methods, a narrative strategy |
| 1. Rotteau  2. 2015 (29) | Descriptive qualitative study, semi-structured interviews. Interviews addressed B/Fs to program implementation; analysed using Braun and Clarke | Hospitals were selected based on change in ED wait times 6 months after program implementation. 3 hospitals with greatest improvement and 2 with least improvement were selected at two waves. Participants were selected using maximum variation sampling with the aim to recruit 5 people per hospitals, who have different roles | 6-8 months | Unclear. Seems as though it is part of the interviews. |
| 1. Sanchez  2. 2014 (30) | Individual SSIs using grounded theory. Interviewers explored respondents’ perceptions  of the medication reconciliation implementation planning process . | Respondents recruited who were involved in the medication reconciliation planning process, selected from: pharmacy directors, chiefs of staff, nurse managers, quality improvement managers, information technology representatives, and physicians with administrative roles. Potential respondents were members of standing or ad hoc planning committees (e.g. patient safety, QI)  and were recruited by email and telephone. Snowball sampling strategy. | 3 years | They used grounded theory and mapped data onto CFIR. Did not measure sustainability specifically. |
| 1. Stacey  2. 2015 (31) | Prospective pragmatic observational study; 2 types of surveys (patient decision aid use and barriers assessment); tracking logs to monitor potential referrals. | HCPs who routinely counseled patients. 1-h presentation on shared decision making was provided at a conference. | Sept 2010 - Aug 2012 | Sustained use of the patient decision aid for 80% of eligible patients at end of year 2 was measured using McNemar's test. Barrier survey responses analysed with a Friedman's test. Content analysis for qualitative survey feedback and conference call notes. |
| 1. White  2. 2011 (32) | Implemented intervention activities and collected weekly data from automated reports. 2 reports run daily. All admissions within a 24 h period were extracted from the admission/ discharge/transfer electronic system. A report on the status of the medication reconciliation for each patient within 48 h of admission was obtained from the electronic medication reconciliation system. | Single hospital site | 27 months | Outcome sustainability is measured by completion rates for medical reconciliation. |

**References**

1. Ament SMC, Gillissen F, Moser A, Maessen JMC, Dirksen CD, von Meyenfeldt MF, et al. Factors associated with sustainability of 2 quality improvement programs after achieving early implementation success. A qualitative case study. J Eval Clin Pract. 2017;23(6):1135-43.

2. Belizan M, Bergh AM, Cilliers C, Pattinson RC, Voce A, Synergy G. Stages of change: A qualitative study on the implementation of a perinatal audit programme in South Africa. BMC Health Serv Res. 2011;11:243.

3. Bergh AM, Kerber K, Abwao S, de-Graft Johnson J, Aliganyira P, Davy K, et al. Implementing facility-based kangaroo mother care services: lessons from a multi-country study in Africa. BMC Health Serv Res. 2014;14:293.

4. Bernstein E, Topp D, Shaw E, Girard C, Pressman K, Woolcock E, et al. A preliminary report of knowledge translation: lessons from taking screening and brief intervention techniques from the research setting into regional systems of care. Acad Emerg Med. 2009;16(11):1225-33.

5. Bhanbhro S, Gee M, Cook S, Marston L, Lean M, Killaspy H. Recovery-based staff training intervention within mental health rehabilitation units: a two-stage analysis using realistic evaluation principles and framework approach. BMC Psychiatry. 2016;16:292.

6. Bouamrane MM, Mair FS. Implementation of an integrated preoperative care pathway and regional electronic clinical portal for preoperative assessment. BMC Med Inform Decis Mak. 2014;14:93.

7. Brady PW, Brinkman WB, Simmons JM, Yau C, White CM, Kirkendall ES, et al. Oral antibiotics at discharge for children with acute osteomyelitis: a rapid cycle improvement project. BMJ Qual Saf. 2014;23(6):499-507.

8. Bridges J, May C, Fuller A, Griffiths P, Wigley W, Gould L, et al. Optimising impact and sustainability: a qualitative process evaluation of a complex intervention targeted at compassionate care. BMJ Qual Saf. 2017;26(12):970-7.

9. Campbell S, Pieters K, Mullen KA, Reece R, Reid RD. Examining sustainability in a hospital setting: case of smoking cessation. Implement Sci. 2011;6:108.

10. Fleiszer AR, Semenic SE, Ritchie JA, Richer MC, Denis JL. An organizational perspective on the long-term sustainability of a nursing best practice guidelines program: a case study. BMC Health Serv Res. 2015;15:535.

11. Fleiszer AR, Semenic SE, Ritchie JA, Richer MC, Denis JL. A unit-level perspective on the long-term sustainability of a nursing best practice guidelines program: An embedded multiple case study. Int J Nurs Stud. 2016;53:204-18.

12. Frykman M, von Thiele Schwarz U, Muntlin Athlin A, Hasson H, Mazzocato P. The work is never ending: uncovering teamwork sustainability using realistic evaluation. J Health Organ Manag. 2017;31(1):64-81.

13. Glasgow JM, Yano EM, Kaboli PJ. Impacts of organizational context on quality improvement. Am J Med Qual. 2013;28(3):196-205.

14. Gould DJ, Hale R, Waters E, Allen D. Promoting health workers' ownership of infection prevention and control: using Normalization Process Theory as an interpretive framework. J Hosp Infect. 2016;94(4):373-80.

15. Gramlich LM, Sheppard CE, Wasylak T, Gilmour LE, Ljungqvist O, Basualdo-Hammond C, et al. Implementation of Enhanced Recovery After Surgery: a strategy to transform surgical care across a health system. Implement Sci. 2017;12(1):67.

16. Green SA, Bell D, Mays N. Identification of factors that support successful implementation of care bundles in the acute medical setting: a qualitative study. BMC Health Serv Res. 2017;17(1):120.

17. Hommel A, Gunningberg L, Idvall E, Baath C. Successful factors to prevent pressure ulcers - an interview study. J Clin Nurs. 2017;26(1-2):182-9.

18. Hovlid EB, O.;Haug, K.;Aslaksen, A. B.;von Plessen, C. Sustainability of healthcare improvement: what can we learn from learning theory? BMC health services research. 2012;12:235.

19. Ilott I, Gerrish K, Eltringham SA, Taylor C, Pownall S. Exploring factors that influence the spread and sustainability of a dysphagia innovation: an instrumental case study. BMC Health Serv Res. 2016;16(1):406.

20. Jangland E, Gunningberg L. Improving patient participation in a challenging context: a 2-year evaluation study of an implementation project. J Nurs Manag. 2017;25(4):266-75.

21. Matthew-Maich N, Ploeg J, Dobbins M, Jack S. Supporting the Uptake of Nursing Guidelines: what you really need to know to move nursing guidelines into practice. Worldviews Evid Based Nurs. 2013;10(2):104-15.

22. Mazzocato PH, R. J.;Brommels, M.;Aronsson, H.;Backman, U.;Elg, M.;Thor, J. How does lean work in emergency care? A case study of a lean-inspired intervention at the Astrid Lindgren Children's hospital, Stockholm, Sweden. BMC health services research. 2012;12:28.

23. McClung L. Health care worker perspectives of their motivation to reduce hospital-acquired infections. Journal of Investigative Medicine. 2017;65(4):824.

24. Mitchell SE, Weigel GM, Laurens V, Martin J, Jack BW. Implementation and adaptation of the Re-Engineered Discharge (RED) in five California hospitals: a qualitative research study. BMC Health Serv Res. 2017;17(1):291.

25. Naldemirci O, Wolf A, Elam M, Lydahl D, Moore L, Britten N. Deliberate and emergent strategies for implementing person-centred care: a qualitative interview study with researchers, professionals and patients. BMC Health Serv Res. 2017;17(1):527.

26. Nordmark S, Zingmark K, Lindberg I. Process evaluation of discharge planning implementation in healthcare using normalization process theory. BMC Med Inform Decis Mak. 2016;16:48.

27. Parand A, Benn J, Burnett S, Pinto A, Vincent C. Strategies for sustaining a quality improvement collaborative and its patient safety gains. Int J Qual Health Care. 2012;24(4):380-90.

28. Robert G, Morrow E, Maben J, Griffiths P, Callard L. The adoption, local implementation and assimilation into routine nursing practice of a national quality improvement programme: the Productive Ward in England. J Clin Nurs. 2011;20(7-8):1196-207.

29. Rotteau L, Webster F, Salkeld E, Hellings C, Guttmann A, Vermeulen MJ, et al. Ontario's emergency department process improvement program: the experience of implementation. Acad Emerg Med. 2015;22(6):720-9.

30. Sanchez SH, Sethi SS, Santos SL, Boockvar K. Implementing medication reconciliation from the planner's perspective: a qualitative study. BMC Health Serv Res. 2014;14:290.

31. Stacey D, Vandemheen KL, Hennessey R, Gooyers T, Gaudet E, Mallick R, et al. Implementation of a cystic fibrosis lung transplant referral patient decision aid in routine clinical practice: an observational study. Implement Sci. 2015;10(1):17.

32. White CM, Schoettker PJ, Conway PH, Geiser M, Olivea J, Pruett R, et al. Utilising improvement science methods to optimise medication reconciliation. BMJ Qual Saf. 2011;20(4):372-80.
