# Supplementary material for: The barriers and facilitators influencing the sustainability of hospital-based interventions: a systematic review
Source: BMC Health Serv Res. 2020 Jun 28;20:588. doi: 10.1186/s12913-020-05434-9 (PMC7321537; doi:10.1186/s12913-020-05434-9)
Supplement: Supplementary file 4 — Additional file 4. Summary of the interventions or programmes delivered. [file 12913_2020_5434_MOESM4_ESM.docx]

**ADDITIONAL FILE 4. TABLE OF INTERVENTIONS OR PROGRAMMES DELIVERED IN THE INCLUDED STUDIES**

Key: cRCT: cluster randomised controlled trial; ED: emergency department; ERAS: Enhanced recovery after surgery; HCP: healthcare professionals; MDT – multidisciplinary team; PPIP: Perinatal Problem Identification Programme; SSP: short stay program

| **Study**  1. First author  2. Year ^(ref)^ | **Name of intervention(s) or programmes** | **Aim of the intervention(s) or programmes** | **1. Materials**  **2. Procedures** | **1. Who provided the intervention**  **2. Mode of delivery**  **3. Location of intervention** | **1. Regimen (when and how much)**  **2. Tailoring?**  **3. Modifications?** | **Key findings** |
| --- | --- | --- | --- | --- | --- | --- |
| 1. Ament  2. 2017 (1) | Two interventions reported: 1) Enhanced Recovery After Surgery program (ERAS) for colonic surgery; 2) Short‐stay program (SSP) for breast cancer surgery | ERAS aimed to enhance functional recovery after surgery and to reduce length of stay after colonic surgery.  SSP sought to increase efficiency of breast cancer surgery care by renewing the patient information strategy and by standardization of the care process, while maintaining the perceived quality of care by patients. | 1. NR  2. Limited details reported. ERAS for colonic surgery: multidisciplinary hospital based program with 18 elements of perioperative care, implemented by using a generic implementation strategy (Breakthrough series characterised by Plan-Do-Study-Act cycles) involving the structured collaboration between hospitals that aim to attain a known best practice. | 1. Unclear. ERAS was disseminated to all “parties within hospital involved in perioperative care; planning, execution and evaluation of change)  2. ERAS: Group; face-to-face  3. ERAS: Ten hospitals were identified as implementation champions were identified for the ERAS protocol. | 1. ERAS: Site visits and formation of local MDT; followed by 2 day start up meeting to discuss ERAS protocol; 3 monthly learning sessions organised by local expert and sharing of experiences. Implementation process finished after 1 year.  2. Yes. Each hospital made an individual project plan for ERAS.  3. Yes. ERAS protocol adapted to local situation | 1. ERAS: “median LOS after colonic surgery reduced by 3 days, from 9 to 6 days after the implementation of the ERAS programme in the participating hospitals. Laparoscopy, female sex, epidural anaesthesia, postoperative use of magnesium oxide, early mobilisation and early cessation of IV fluids were identified factors associated with an increased probability of shorter LOS, whereas ASA III-IV was an identified factor associated with a decreased probability of shorter LOS. The implementation was successful, and a dramatic change in perioperative routines was achieved, abandoning nasogastric tubes, abandoning bowel preparation before surgery and starting early nutrition after surgery” (Additional file 2)  SSP for breast cancer surgery: “a hospital-tailored implementation strategy aimed at overcoming the specific barriers to implementation in each participation hospital”. Implemented in 4 hospitals in the Netherlands between 2005 and 2007. Key outcomes were maintained 5 years postimplementation. |
| 1. Belizan  2. 2011 (2) | The Perinatal Problem Identification Programme (PPIP) | To introduce HCPs to an audit tool for the improvement of the quality of perinatal care in the public healthcare sector. | 1. Computer, database  2. PIPP database is a programme developed in 1990s which determines perinatal mortality rates plus avoidable factors assoc. with each death. It is an audit and feedback system that helps structure data and makes audit easier Not a national database. Feedback at multiple levels including daily/weekly ward meetings where deaths occurred; monthly hospital review meetings; annual regional, provincial or national levels to report on aggregated death rates and causes of death. | 1. MRC unit provides training sessions and updates in PIPP software “on a regular basis when there is a demand”. Co-ordinators now frequently manage the process and collate data, but managers or clinicians “take responsibility for installing software and organising the data entry”  2. Online audit database  3. Online | 1. Data is entered as required, feedback has an important role in implementing and maintaining the audit programme.  2. NR 3. NR | 4 essential factors important for sustainability of audit system: 1) drivers (agents of change) and teamwork, 2) clinical outreach visits and supervisory activities, 3) institutional perinatal review and feedback meetings, and 4) communication and networking between health system levels, health care facilities and different role-players. During the pre-implementation phase high perinatal mortality rates highlighted the problem and indicated the need to implement an audit programme (stage 1). Commitment to implementing the programme was achieved by obtaining buy-in from management, administration and health care practitioners (stage 2). Preparations in the implementation phase included the procurement and installation of software and training in its use (stage 3). Implementation began with the collection of data, followed by feedback at perinatal review meetings (stage 4). The institutionalisation phase was reached when the results of the audit were integrated into routine practice (stage 5) and when data collection had been sustained for a longer period (stage 6). |
| 1. Bergh (3)  2. 2014 | Kangaroo mother care (KMC) Ghana initiative | To decrease the risk of death in neonates weighing < 2000 g | 1. KMC progress monitoring tool (see <https://www.ncbi.nlm.nih.gov/pmc/articles/PMC4104737/#S1>)  2. Three phase outreach intervention incl. placing infant skin-to-skin against the mother’s chest (i.e. continuous KMC, 24/7 and/or intermittent KMC (recurrent but not continuous skin-to-skin contact between mother and baby, a few times a day) over a variable no. of days); exclusive breastfeeding where possible; and early discharge from the health facility, with appropriate follow-up systems. | 1. Project manager (medical doctor) plus 3 international facilitators with backgrounds in health systems research, neonatology, and nursing education and management, coordinated in collaboration with regional KMC steering committees (SC)  2. Face-to-face; 1:1  3. Hospitals and other outreach health facilities | 1. Intervention regimen varied depending on continuous or intermittent contact.  2. Context appropriate adaptations. Based on an earlier RCT which recommended “choice of outreach strategy should be guided by local circumstances, cost, and availability of skilled facilitators”  3. Yes. See notes above. | “Institutions that fared better had a longer history of KMC implementation or had been developed as centres of excellence or had strong leaders championing the implementation process. Variation existed in the quality of implementation between facilities and across countries. Important factors identified in implementation are: training and orientation; supportive supervision; integrating kangaroo mother care into quality improvement; continuity of care; high-level buy in and support for kangaroo mother care implementation; and client-oriented care” |
| 1. Bernstein  2. 2009 (4) | Taking Screening, brief intervention techniques and referral to treatment (SBIRT) in ED | To screen patients and refer them community resources or substance abuse treatment | 1. Space for consultation; phone, computer; transportation (if required)  2. ED-SBIRT is a multi-component intervention based on brief motivational interviewing. HCPs obtain patient permission, patient perceptions of the pros and cons of substance abuse, feedback on guidelines, reflection on the discrepancies between current life circumstances and future goals, assessment of readiness to change, eliciting reasons for change, identifying strengths and prior successes, providing resources and developing a specific action plan. Follow-up and referrals (if indicated). | 1. Delivered by HCPs in ED. Originally 12 champions were trained and then they hosted and taught local site SBIRT workshops to introduce the program and the skills set to the entire ED staff.  2. Face-to-face; 1:1  3. ED | 1. Brief (length of intervention not specified)  2. Yes, based on local context.  3. Yes. (“Integration of the HPAs into the ED culture and team required adaptation.”) | 5/7 sites were sustained through second year of the project despite funding cuts. Successful implementation depended on external start-up funding; local ED champions; sustainability planning; robust referral network. |
| 1. Bhanbhro  2. 2016 (5) | GetREAL - rehabilitation effectiveness for activities for life | To increase the confidence and skills of staff working in inpatient mental health rehabilitation units in engaging service users in activities | 1. Computer, internet, manual for the intervention, a fidelity checklist, an induction programme and training materials were produced (see Cook 2015 (6) for more details).  2. Three stages; predisposing, enabling and reinforcing. In the predisposing stage two senior members of the research team visited each unit to gain local “sign up” and ensure the intervention team would be appropriately supported. The enabling stage involved the intervention team working alongside the rehabilitation unit staff for five weeks to deliver training and modelling in specific processes and skills related to improving service user engagement in activities. At the end of the enabling period an Action Plan was agreed that clarified the changes to structures and processes the unit would continue with and identified a member of staff who would act as the Unit’s “champion”. The reinforcing stage aimed to maintain the new skills the staff had learnt and the changes to structures and process they had agreed on during the enabling stage by providing ongoing, regular email contact between the unit staff and the intervention team for 12 months. | 1. Research team, intervention team (an occupational therapist, an activity worker and a service user expert) and rehabilitation staff champion  2. Face-to-face; 1:1 and group  3. Inpatient mental health rehabilitation units | 1. At least 5 weeks with follow-up over 12 months.  2. Unclear. Training sessions were mandated in some units and not others.  3. Unclear. | The intervention was evaluated using a cRCT in 35 units “but there was no clinical advantage over UC and did not increase service user engagement in activities”. The authors of the qualitative case study noted that “in the context of long-term change, there was no single measure that sustains long- term change in practice for NHS rehabilitation units. Rather, that several interconnected measures need to be considered prior, during and after a new programme is introduced”. |
| 1. Bouamrane and Mair  2. 2014 (7) | Integrated preoperative care pathway and regional electronic clinical portal for preoperative assessment (ICP/eForm) | To streamline, standardise and integrate preoperative assessment processes as part of the Planned Care Improvement Programme (PCIP) | 1. Computer, internet  2. Implemented an electronic preoperative integrated care pathway (eForm) allowing all hospitals to access a comprehensive patient medical history via a clinical portal on the health-board intranet. Development of standard preoperative processes across the health-board was later instrumental in allowing the implementation of an electronic Integrated Care Pathway (ICP/eForm) to support documentation and information- sharing tasks across the MDT. | 1. NA  2. Online  3. Hospital intranet | 1. NR  2. NR  3. NR | “adoption of the eForm due to:(i)a policy context – including performance targets – promoting the rationalisation of surgical pre-assessment pathways, (ii) financial and organisational resources to support service redesign and the use of IT for operationalising the standardisation of preoperative processes, (iii) a sustained engagement with stakeholders throughout the iterative phases of the preoperative clinics redesign, guidelines standardisation and the eForm development, (iv) the use of a pragmatic and domain-agnostic technology solution and finally: (v) a consensual and contextualised implementation” |
| 1. Brady  2. 2014 (8) | Rapid and sustained evidence adoption for children on oral antibiotic therapy | QI project with SMART aim of rapidly increasing the percentage of patients’ routine osteomyelitis discharged from the hospital medicine service on oral antibiotic therapy | 1. Computer, internet, SDM tool available at https://www.cincinnatichildrens.org/service/j/anderson-center/evidence-based-care/shared  2. Multifaceted intervention – bundle of 7 interventions (see Figure 1, (8)). Specifically, four improvement activities: (1) education to hospital medicine faculty, residents and students, (2) a process, we term ‘identify and mitigate,’ where a research assistant used the electronic health record to identify patients with potential osteomyelitis and then contacted the faculty and residents caring for the patient to remind the providers about decision support tools to promote evidence adoption, including, (3) a shared decision making (SDM) tool to engage patients and families in decision and (4) a computerised order set linked to an evidence statement. | 1. Hospital medicine faculty members were educated on the evidence and application of early transition to oral antibiotic therapy. Education then spread to the remaining hospital faculty and residents/ students. Local expert opinion was also sought. An RA identified and new patients/ week.  2. Face-to-face; 1:1 and Group; Email  3. Large academic children’s hospital | 1. All patients were identified “in near-real time (within 1-2 days)  2. Tailored for each patient.  3. Yes. SDM not appropriate in one case. | “We achieved significant and sustained improvement through a series of interventions that were tested and implemented into our care process within a 7-month period of time”  “Improvement has been sustained for 1 year. Treatment failure and complications were uncommon in preintervention and postintervention phases. No significant differences in length of stay or charges were detected” |
| 1. Bridges  2. 2017 (9) | Creating Learning Environments for Compassionate Care (CLECC) | To create a sustainable expansive learning environment, through leadership and team practices, that enhances team capacity to provide compassionate care. | 1. Meeting rooms  2. CLECC is a workplace educational intervention focused on developing sustainable leadership and work-team practices (dialogue, reflective learning, mutual support, role modelling), designed to support team relational capacity and compassionate care delivery.  Combination of activities: regular CLECC meetings between ward manager and matron; ward manager action learning sets, including one on influencing senior managers; team learning activities, including climate analysis and values clarification; peer observations of practice; team study days; mid-shift 5 min cluster discussions; and twice weekly reflective discussions. Teams also develop a learning plan to be shared with a senior hospital manager that includes sustainability measures for practices that underpin the delivery of compassionate care. See Table 1 for summary of set activities | 1. 4-month implementation period facilitated by senior practice development nurse  2. Face-to-face; 1:1 and Group  3. Hospital ward | 1. Regular meetings between ward manager and manager; peer observations of practice; team study days; mid-shift 5min cluster discussions; and twice weekly reflective discussions  2. Yes. The PDNs described in the paper had different leadership approaches for organising CLECC activities.  3. Unclear | “Frontline staff were keen to participate in CLECC, were able to implement many of the planned activities and valued the benefits to their well-being and to patient care. Nonetheless, factors outside of the direct influence of the ward teams mediated the impact and sustainability of the intervention. These factors included an organisational culture focused on tasks and targets that constrained opportunities for staff mutual support and learning” |
| 1. Campbell  2. 2011 (10) | The Ottawa Model for Smoking Cessation (OMSC) | To help inpatients stop smoking | 1. Phone, patient records  2. Hospital-based smoking cessation program. Multi-component intervention with 5 activities: identify smokers on admission, document smoking status on patient record, provide advice and behavioural support with quitting, offer smoking cessation medication during the hospital stay, offer follow up support upon discharge. Follow up is monitored by an automated interactive voice response system that tracks patients for six months | 1. Smoking cessation coordinator at each hospital (usually unit nurses, program managers or dedicated role); hospitals were asked to make OMSC activities part of normal hospital routine.  2. Face-to-face; 1:1, interactive voice system  3. Hospital ward | 1. NR  2. Yes. Tailored to individual patient  3. Yes. Hospitals implemented the intervention in different ways. See additional file 2 for more details. | “Success of the program is dependent upon the ability of hospitals to sustain the program in the clinical setting over time, despite competing priorities. Using program champions, incorporating relevant performance feedback, conducting ongoing education, training, and promotion, designating a hospital-based coordinator role, and demonstrating program effectiveness emerged as important factors for sustainability of the OMSC” |
| 1. Fleiszer  2. 2015 (11) | Best practice guidelines (BPG): organisation level perspective | To improve nursing care and patient outcomes, harmonize practices, expand knowledge translation and performance measurement capacities and develop nursing leadership competence across the organization | 1. Computer, database, internet, reports  2. Patient safety oriented BPG program consisting of:   - Organisation-wide implementation: of three Registered Nurses’ Association of Ontario (RNAO) nursing BPGs: falls prevention, pressure ulcer prevention, and pain management. - Nursing department level: multiple changes were made to establish the BPG program across the health centre. These included designating program co- chairs, creating a steering committee, formalizing BPG- specific task forces, training volunteer practice change advocates, employing a part-time program coordinator, achieving RNAO “Best Practice Spotlight Organization” program status, and securing financial support from numerous sources. - Activities at the department level were related to education (e.g., organization-wide workshops about BPGs and evidence- informed practice change), equipment (e.g., purchase of BPG-recommended patient care items), and evaluation (e.g., establishment of an annual prevalence survey and modification of BPG-related data collection and reporting systems). - Activities at nursing unit level: to improve staff nurses’ performance of patient care and documentation practices based on the BPGs. Department-level BPG task force members and change advocates worked with individual nursing units over two to three month-long periods to sup- port the initial implementations of each of the three BPGs. Those externally facilitated implementation “start-ups” used a combination of educational sessions, nurse BPG champion support, and audit and feedback activities to help nursing units integrate BPG standards into daily practice. | 1. NA. Variety of roles in the program (e.g., program co-directors, task force members, coordinator, change advocates)  2. Face-to-face, remote, online, 1:1 and group.  3. Large, tertiary/quaternary urban academic acute health centre with 6 hospital sites, eight clinical missions/programs, 47 inpatient units, approximately 1000 staffed beds, and more than 3000 nurses | 1. NR  2. Yes. Depended on target level of change.  3. Unclear | “A constellation of 11 factors most influenced the long-term sustainability of the program. These factors were innovation-, context-, leadership-, and process-related. Three key interactions between factors influencing program sustainability and characteristics of program sustainability accounted for how the program had been sustained. These interactions were between leadership commitment and benefits; complementarity of leadership actions and both institutionalization and development; and a reflection-and-course-correction strategy and development” |
| 1. Fleiszer  2. 2016 (12) | As above but focus of this study was unit level perspective | As above | As above | As above | As above | “Program sustainability was characterized by three elements: benefits, routinization, and development. Seven key factors most accounted for the differences in the level of program sustainability between subcases. These factors were: perceptions of advantages, collaboration, accountability, staffing, linked levels of leadership, attributes of formal unit leadership, and leaders' use of sustainability activities. Some prominent relationships between characteristics and factors explained long-term program sustainability. Of primary importance was the extent to which unit leaders used sustainability-oriented activities in both regular and responsive ways to attend to the relationships between sustainability characteristics and factors” |
| 1. Frykman  2. 2017 (13) | Implementation of multi-professional teamwork: behaviour change interventions | Teamwork was implemented as a response to directives introduced by the county council to reduce waiting times. | 1. Work room and examination rooms  2. The main feature of the teamwork was the allocation of each patient to a specific team consisting of a physician, an RN, and an LPN. Each team was assigned a work room and specific examination rooms. A routine of holding team start-up meetings at the beginning of the shift was introduced. At the end of the shift, feedback and a short evaluation of the shift’s teamwork were performed. The desired teamwork behaviours were care team members assemble when tasks have been performed, work in parallel, communicate the work plan, team members coordinate work, and communicate decision to change the plan. | 1. A physician (a resident in emergency medicine) was assigned the role of full-time change facilitator and formed a change team together with the group leaders (RNs managing the daily operations of the ED). The change team was assisted by two external performance consultants since the decision to implement teamwork.  2. Face-to-face, 1:1 and groups  3. Section for internal medicine at an ED at a university hospital in Sweden. The ED employed 120 nurses (registered nurses (RNs) and licensed practical nurses (LPNs)) and approximately 180 physicians who were employed within different specialties at the hospital and worked shifts at the ED as part of their employment contracts. | 1. NR  2. Yes. Patient focused.  3. Yes. Schedule, staff resources and facilities were adapted (see Appendix) | “Teamwork behaviours were not sustained. A substantial fallback in managerial activities in combination with a complex context contributed to reduced direction, opportunity, and motivation. Reduced direction made staff members unclear about how and why they should work in teams. Deterioration of opportunity was evident from the lack of problem-solving resources resulting in accumulated barriers to teamwork. Motivation in terms of management support and feedback was reduced” |
| 1. Glasgow  2. 2013 (14) | The Flow Improvement Inpatient Initiative (FIX): A national improvement collaborative | To improve inpatient hospital flow by reducing hospital length of stay (LOS) and increase percentage of patients discharged before noon | 1. Computer, phone, internet access  2. “Each region had a leadership team that consisted of two co-directors (responsible for overall leadership, recruiting and serving as core faculty) and two co-coordinators (responsible for day-to-day management including communication, agenda logistics, facilitating phone call sessions and troubleshooting). These individuals were selected to provide an optimal mix of positional authority, teaching skills, knowledge, internal veterans affairs (VA) experience and the ability to work effectively in a high-performing team. QI coaches were recruited based on their expertise in improvement methods and experience with inpatient flow and were responsible for working with the QI teams and for providing verbal and written feedback throughout the change journey. A close connection was maintained between the QI teams and their regional leadership team as well as the national steering committee, ensuring alignment to the core messages, content and strategies. Teams were encouraged to follow the VA–TAMMCS improvement framework which calls for first selecting a topic and focus (vision, analysis), identifying a team (T), adopting clear aims (A), flow-mapping and measuring the process (MM), running plan, do, study, act change cycles (C), and lastly working to sustain and spread improvements (S). Each team worked to translate the FIX principles into solutions addressing their unique flow concerns, as such solutions varied across sites” (15). | 1. Each hospital ensured that two to three individuals from the improvement team (one of whom had to be a hospital executive) participated in each learning session, although some individuals varied across learning sessions.  2. Face-to-face, 1:1, group, online  3. Participation in the collaborative was mandatory, with the 130 participating hospitals organised into five geographic regions (Northeast, Southeast, Central, Midwest and West) consisting of approximately 26 hospitals each (15). | 1. The improvement teams participated in a telephonic ‘pre-work’ session as well as three face-to-face 1.5–2-day long learning sessions that focused on teaching key flow principles for measuring and addressing demand variation as well as change management tools (15).  2. Unclear.  3. Unclear. | “The decision trees did not find any predictive associations in this sample of 100 hospitals participating in a national QI collaborative. Further model review identified that measures of QI Experience were associated with an ability to make improvements, whereas measures of Staffing and Culture were associated with an ability to sustain improvements. A key area for future research is to understand the challenges faced as QI teams’ transition from improving care to sustaining quality and to ascertain what organizational characteristics can best overcome those challenges” |
| 1. Gould  2. 2016 (16) | Infection prevention control | To address failings in prevention and control of health care infections especially C. Difficile | 1. Handwashing facilities, policy documents, deep cleaning equipment  2. Action plan involved:   - Renewed hand hygiene campaign, - Deep cleaning of all clinical areas, - 2h targets for isolation infections and reduction in infections, - Champion group, - Emphasising on specific policies, - Attendance at committee meetings, - Regular discussions, monthly metrics, - Review of infection cases - Identification of strategies to reduce C. Difficile   Further details are shown in Figure 2 (16). | 1. Everyone involved in healthcare delivery but led by a C. diff champion group convened with membership comprising general managers, divisional medical and nursing directors meeting every 2 weeks  2. Face-to-face, 1:1 and groups  3. Health board serves an urban and rural population of 600,000 people in South Wales, and provides a full range of acute, intermediate, primary and community care services. Acute care is concentrated in four hospitals. The organization employs 10,000 staff directly involved in patient care | 1. Not specified but regular meetings in place - see procedures section.  2. NR  3. NR | “Six themes emerged through inductive analysis. Theme 1: ‘Ability to make sense of ownership’ provided evidence of the first element of NPT (coherence). Regardless of occupational group or seniority, informants understood the importance of IPC ownership and described what it entailed. They identified three prerequisites: ‘Always being vigilant’ (Theme 2), ‘Importance of access to information’ (Theme 3) and ‘Being able to learn together in a no-blame culture’ (Theme 4). Data relating to each theme provided evidence of the other elements of NPT that are required to embed change: planning implementation (cognitive participation), undertaking the work necessary to achieve change (collective action), and reflection on what else is needed to promote change as part of continuous quality improvement (reflexive monitoring). In- formants identified barriers (e.g. workload) and facilitators (clear lines of communication and expectations for IPC)… Eighteen months after implementing the action plan incorporating IPC ownership, there was evidence of continuous service improvement and significant reduction in infection rates” |
| 1. Gramlich  2. 2017 (17) | Enhanced Recovery After Surgery (ERAS) | ERAS care system includes an evidence-based guideline, an implementation program, and an interactive audit system to support practice change to enhance surgical care in colorectal surgery | 1. Guideline documentation, databases, educational materials  2. ERAS – also reported in Ament 2017 study (see earlier entry). Guidelines bundle 22 interventions for colorectal surgery (before, during and after).   - Perioperative interventions included: PAC patient education, PAC shared decision making, PAC nutrition, PAC medical optimization, Fluid and carb loading, no prolonged fasting, no/selective bowel prep, antibiotic prophylaxis. - Intraoperative interventions included: thromboprophylaxis, no premedication, nausea and vomiting prophylaxis, short-acting anaesthetic agents, no drains, avoidance of salt and water overload, maintenance of normothermia. - Postoperative interventions included: mid-thoracic epidural anaesthesia / analgesic, no nasogastric tubes, prevention of nausea and vomiting, avoidance of salt and water load, early removal of catheter, early oral nutrition, non-opioid oral analgesia / NSAIDs, early mobilisation, stimulation of gut motility, audit of compliance and outcomes   The implementation program in each site followed QUERI and Plan Do Study Act cycles and staff were provided support (see WHO section) | 1. HCPs in surgical unit. The ERAS implementation program (EIP) included detailed coaching and supervision of an implementation team in “Train the Trainer” sessions, including a surgeon as the local leader in practice, an anesthesiologist, and a nurse leader acting as the coordinator, at a given site in a particular surgical area.  2. Face-to-face, 1:1 and group.  3. Surgical units | 1. NR  2. Yes. tailored for each individual  3. Yes. Local modifications to protocol. | “Compliance with the evidence-based guidelines for ERAS in colorectal surgery at baseline was 40%. Post implementation compliance, consistent with adoption of best practice, improved to 65%. Barriers and enablers were categorized as clinical practice (22%), individual provider (26%), organization (19%), external environment (7%), and patients (25%). In the Alberta context, 26% of barriers and enablers to ERAS implementation occurred at the site and unit levels, with a provider focus 26% of the time, a patient focus 26% of the time, and a system focus 22% of the time” |
| 1. Green  2. 2017 (18) | Two implementation initiatives:  a) COPD care bundle and  b) diabetic foot care bundle | Two care bundles that explicitly aimed to implement clinical guidelines through developing a new care bundle for use in the acute medical setting and demonstrated improvements in compliance with process measures. One focused on chronic obstructive pulmonary disease (COPD) and the other on diabetic foot care. | 1. Computer, written materials for patient, inhalers  2. COPD bundle:   - Respiratory nurse notified of admission - Smokers offered smoking cessation assistance - Referral to pulmonary rehabilitation for assessment - Provision of written information about COPD - Assessment and demonstration of satisfactory inhaler use - Follow-up appointment with specialist   Diabetic foot bundle   - Presence or absence of Ulcers - Presence or absence of infection (Fever, Low BP, Red and/or warm foot, pain without trauma) - Presence or absence of ischaemia (Absent foot pulses, Cold or gangrenous foot) - Presence or absence of deformity [Charcot Foot] (Foot does not look normal) - Positive result- manage appropriately | 1. COPD bundle:  “Respiratory consultants, clinical nurse specialists, pharmacist, patient advisor, physiotherapist, smoking cessation specialist, project manager (nurse) and QI advisors”  Diabetic foot bundle:  “Endocrinology consultants, patient advisors, podiatrist ward/specialist nurses, project manager (nurse), research nurse and QI advisors”  2. NR  3. Both bundles were delivered in an acute medical unit/ ward | 1. NR but delivered during admission  2. Yes. Tailored to patient needs  3. NR | “Several factors were identified that directly influenced the implementation of the care bundles. Firstly, the availability of resources to support initiatives, which included training to develop quality improvement skills within the team and building capacity within the organisation more generally. Secondly, the perceived sustainability of changes by stakeholders influenced the embedding new care processes into existing clinical systems, maximising their chance of being sustained. Thirdly, senior leadership support was seen as critical not just in supporting implementation but also in sustaining longer-term changes brought about by the initiative. Lastly, practitioner incentives were identified as potential levers to engage junior doctors, a crucial part of the acute medical work force and essential to the initiatives, as there is currently little recognition or reward for involvement” |
| 1. Hommel  2. 2017 (19) | Swedish Association of Local Authorities and Regions (SALAR) national pressure ulcer patient safety study | To prevent pressure ulcers in hospital settings | 1. Action plans, pressure relieving equipment  2. Multicomponent interventions based on best practice guidelines: timely identification of high-risk patients, systematic skin inspection, preventive interventions such as heel protection/floating heels and sliding sheets, pressure reducing cushions, nutritional support, repositioning and mobilisation. One part of the agreement was economic compensation for registration of PU in a national registry, Senior Alert. | 1. Managers, physicians, registered nurses, enrolled nurses with different kind of responsibilities  2. Face-to-face, 1:1  3. National programme but this study was based on 6 hospitals (two university hospitals, two central hospitals and two local hospitals) in different county councils in Sweden | 1. NR  2. Yes. Individualised (“Nurse managers who coach their front-line nurses to adapt patient care processes in response to identified patient needs”)  3. NR | “Three main categories were identified as successful factors to prevent pressure ulcer in hospitals: creating a good organisation, maintaining persistent awareness and realising the benefits for patients. The goal for all healthcare personnel must be delivering high-quality, sustainable care to patients. Prevention of pressure ulcer is crucial in this work. It seems to be easier for small hospitals (with a low number of units/beds) to develop and sustain an effective organisation in prevention work. The nurse managers’ attitude and engagement are crucial to enable the personnel to work actively with pressure ulcer prevention” |
| 1. Hovlid  2. 2012 (20) | QI for elective surgery pathway | To redesign the pathway for elective surgery to reduce cancellations | 1. Computer, meeting room  2. Four different project groups with a total of 40 employees were formed. Each group was given a mandate to re- design parts of the pathway. The changes that were implemented included one common entry point for all referrals, earlier clinical patient assessment, improved information flow among staff members, patient participation in selecting the date for surgery, and improved coordination and scheduling of operations. | 1. Unclear, but “40 different [HCP, admin and managers] employees were involved”  2. Face-to-face, group  3. District general hospital in a small town in Norway, population 10,000. The hospital has 7 operating suites and 34 surgical beds. The project involved the surgical departments at the hospital (ophthalmology, general surgery, gynaecology, orthopaedics, and ear, nose, and throat). | 1. NR  2. Yes. Patients selected date for surgery.  3. Yes. (“Clinicians were involved in modifying and adapting the interventions to the context.”) | “Clinicians and leaders shared information about their everyday work and related this knowledge to how the entire clinical pathway could be improved. In this way they developed a revised and deeper understanding of their clinical system and its interdependencies. They became increasingly aware of how different elements needed to interact to enhance the performance and how their own efforts could contribute. The improved understanding of the clinical system represented a change in mental models of employees that influenced how the organization changed its performance. By applying the framework of organizational learning, we learned that changes originating from a new mental model represent double-loop learning. In double-loop learning, deeper system properties are changed, and consequently changes are more likely to be sustained” |
| 1. Ilott  2. 2016 (21) | Dysphagia innovation | To promote dysphagia as a patient safety issue | 1. Computer, internet, educational policies, e-learning programmes, the South Yorkshire dysphagia Toolkit available at <http://www.dysphagiatoolkit.nihr.ac.uk>  2. A locally developed innovation that involved using the Inter-Professional Dysphagia Framework to structure education for the workforce. Interventions were categorised according to Ovretveit's framework (e.g. organisation wide education policies, a train-the-trainer intervention, additional activities in response to requests). The Inter-Professional Dysphagia Framework (IPDF) was developed by several professional bodies to offer a consistent approach to competency development for knowledge and skills in dysphagia in the UK. Comprises 5 levels, from Awareness which introduces the risks of dysphagia, through to Consultant Dysphagia Practitioners who undertake specialist investigations, manage complex cases and contribute to research. We chose to focus on the second level: Assistant Dysphagia Practitioner because it covers the knowledge and skills needed to support safe swallowing and applies to anyone who assists patients to eat and drink. | 1. Speech and language therapist  2. Face-to-face, group, online  3. Publicly funded health care facility in England (NHS Trust). Employed around 15,500 staff serving in excess of 1 million patients each year. The clinical level included hospital wards and a community unit providing acute care and rehabilitation for patients on the care pathways for stroke and fractured neck of femur. Care pathways comprised 7 wards, 5 in hospital and 2 in the community | 1. Three-hour session with a speech and language therapist (SLT), a Dysphagia Toolkit with teaching resources and information, three e-learning programmes which contained the essential knowledge, out-reach visits by the SLT, written and verbal feedback; and additional activities in response to requests.  2. Not explicit but additional activities suggest tailoring.  3. Yes. Adaptation reported in Table 2. Examples included additional activities such as staff notices, adapted crockery /cutlery plus informal sharing knowledge etc | Leadership, critical junctures, temporality and making the innovation routine were aspects of hierarchical control. Participatory adaptation was evident on the care pathways through stakeholder responses, workload and resource pressures. Six of the 25 ward-based trainers cascaded the dysphagia training. The expected outcomes were achieved when the top-down mandate (hierarchical control) was supplemented by local engagement and support (participatory adaptation) …. the findings illustrate the dualities of organisational change as universal and context specific …appreciating these dualities may contribute to understanding why many innovations fail to become routine.” |
| 1. Jangland and Gunningberg  2. 2017 (22) | The Tell-us card | To stimulate patient participation in surgical care units | 1. Book about patient experiences used as a reflective tool, data from national surveys, written information and reminders for patients, the Tell-us card, flyers and department website.  2. Based on PCC, the Tell-us card was used during interactions between patients and health care professionals such as nurses’ rounds, ward rounds and discharge information sessions. Patients filled in their goals for the day as well as their concerns on these cards, which were used as a tool for communication with all health care professionals. It was important to invite the patient to participate and to ask, ‘What matters to you?’. Activities are detailed in Table 1 (22). The Tell-us card is shown in Figure 1 (22). | 1. The leaders of the department supported the project. A project group was put together including external and internal facilitators (n = 8) from each unit. The internal facilitators were RNs with the specific responsibility of implementing the Tell-us card in their units and educating their colleagues about the value of patient participation. These internal facilitators were identified by the nurse manager through the interest they showed in the project. The project was led and planned by an external facilitator, the first author, with long clinical experience in quality improvement  2. Face-to-face, 1:1 or group (if families present) and online materials.  3. Surgical department in a large university hospital in Sweden. The department consisted of five surgical units. Admitted mainly adult patients from the emergency department, or on a waiting list for surgery. About 350 health professionals worked in the department. Each unit had 15–23 beds with a nurse manager responsible for nursing care and a senior consultant responsible for medical care. All nurse managers had their office on the ward and worked closely with staff on a daily basis. | 1. NR  2. Yes. Although the tell-us card was standardised, other parts of the intervention were individualised.  3. NR | “the long-term implementation project did not improve patient participation in the units, the nurse managers described a changing culture in which staff grew to accept patients’ involvement in their own care. Several barriers to change and sustainability were acknowledged” |
| 1. Matthew-Maich  2. 2013 (23) | Breastfeeding best practice guidelines (BPG) implementation and uptake in nursing practice | To improve implementation of breastfeeding BPG | 1. Relevant policies, documentation forms, patient teaching resources, memos, newsletters, and BPG reports. Updated guidelines are available at <https://rnao.ca/bpg/guidelines/breastfeeding-promoting-and-supporting-initiation-exclusivity-and-continuation-breast>  2. The guidelines include 16 recommendations covering a range of topics, including assessment of the breastfeeding process, skin-to-skin contact, responsive cue-based breastfeeding, and implementing the provisions of the Baby-Friendly Initiative. | 1. Nurses and midwives  2. Face-to-face; 1:1  3. Three acute care hospitals | 1. NR  2. Yes. Tailored to patient.  3. NR | “Two sites showed BPG uptake while one did not. Factors present in the uptake sites included, ongoing passionate frontline leaders, the use of multifaceted strategies, and processes that occurred at organizational, leadership, individual and social levels. Particularly noteworthy was the transformation of individual nurses to believing in and using the BPG. Impacts occurred at client, nurse, unit, inter-professional, organizational and system levels” |
| 1. Mazzocato  2. 2012 (24) | Lean inspired intervention to improve patient flow in paediatric ED | To improve all emergency patient flows which constituted over 60% of all hospital admissions, including the pediatric patient flow at the studied A&E. | 1. Meeting rooms, fax referrals, computer, internet.  2. Multicomponent intervention developed based on several Improvement Principles mainly drawn from lean thinking and suggested by coaches. The final ‘prototype” included  including:   - Multi-professional care team approach and physical work setting redesign - Centralised management and control of patient flow, and information technology - Increased staffing and involvement of senior physicians - Work schedule changes - New roles and job descriptions - Team approach to problem solving and continual improvement - Monthly meetings with the management group | 1. Recently qualified paediatrician led a team composed of nurses, nurse’s aides, and physicians in redesigning the paediatric care flow at the Children’s Hospital’s A&E. The team was guided by a group of process improvement coaches led by a senior cardiologist with extensive process improvement experience  2. Face-to-face, 1:1 and groups  3. Swedish paediatric Accident and Emergency department. | 1. NR  2. NR  3. Yes. Multiple iterations of the prototype | “Improvements in waiting and lead times (19-24%) were achieved and sustained in the two years following lean-inspired changes to employee roles, staffing and scheduling, communication and coordination, expertise, workspace layout, and problem solving. These changes resulted in improvement because they: (a) standardized work and reduced ambiguity, (b) connected people who were dependent on one another, (c) enhanced seamless, uninterrupted flow through the process, and (d) empowered staff to investigate problems and to develop countermeasures using a “scientific method”. Contextual factors that may explain why not even greater improvement was achieved included: a mismatch between job tasks, licensing constraints, and competence; a perception of being monitored, and discomfort with inter-professional collaboration” |
| 1. McClung  2. 2017 (25) | Bundles of health care associated infection interventions | To reduce healthcare associated infections | 1. Handwashing facilities, personal protective equipment, protocols.  2. Implemented bundles of evidence-based behavioural interventions to prevention/reduce the major types of HAI: central line–associated bloodstream infection (CLABSI), catheter-associated urinary tract infection (CAUTI), surgical site infection, methicillin-resistant *Staphylococcus aureus* bacteremia, and *Clostridium difficile* infection (CDI). Interventions include adherence to evidence-based practices, such as:   - handwashing, - eliminating unnecessary use of medical devices, - attention to insertion and maintenance protocols for devices, - consistent use of personal protective equipment   No other details provided. | 1. Healthcare professionals – involved in the implementation of HAI. This included nursing assistants, nurse champions, environmental service managers, trainee physicians, at- tending physicians, and physicians with administrative roles.  2. NR  3. Large university hospital with 592 staffed beds and a level 1 trauma centre | 1. NR  2. NR  3. Not explicit but authors report “tendency for ‘information overload’ with new policies and constant changes in protocols” | “..patient safety and improvement in clinical outcomes were the major motivators to reducing HAIs. Other important motivators included collaborative environment that valued individual input, transparency and feedback at both organizational and individual levels, leadership involvement, and refresher trainings and workshops. We did not find policy, regulatory considerations, or financial penalties to be important motivators” |
| 1. Mitchell  2. 2017 (26) | Project Re-Engineered Discharge (RED) | An evidence-based strategy to reduce readmissions disseminated and adapted by various health systems across the country. | 1. RED toolkit, education, computers, telephone, written discharge plan  2. Multicomponent intervention.  Twelve components focused on key aspects of the discharge process:   - Ascertain need for and obtain language assistance - Make appointments for follow-up medical appointments and post discharge tests/labs - Plan for the follow up of results from lab tests or studies that are pending at discharge - Organize post-discharge outpatient services and medical equipment - Identify the correct medicines and a plan for the patient to obtain and take them. - Reconcile the discharge plan with national guidelines - Teach a written discharge plan the patient can understand - Educate the patient about his or her diagnosis - Assess the degree of the patient’s understanding of the discharge plan - Review with the patient what to do if a problem arises - Expedite transmission of the discharge summary to clinicians accepting care of the patient - Provide telephone reinforcement of the Discharge Plan   See Additional File 1 for more details of each component. | 1. A team of researchers from Boston Medical Center where Project RED originated trained each hospital’s implementation team onsite for one day on implementing the RED toolkit (Additional file 1), and provided resources on how to deliver RED, how to monitor RED implementation and outcomes  2. Face-to-face; 1:1  3. Five hospitals in California (see Table 1 for detailed hospital profile) | 1. One day on-site training  2. Yes. Not explicit but patient education and medication reconciliation linked to individualised care  3. Yes. “adaption of Project RED is necessary for a sustainable implementation, but adaptations must maintain a high level of fidelity” | “Both internal and external contextual factors were identified that influenced hospitals' decisions on RED adaptation and implementation. These also impacted RED sustainability. External factors included: impending federal penalties for hospitals with high readmission rates targeting specific diagnoses, and access to external funding and technical support to help hospitals implement RED. Internal or organizational level contextual factors included: committed leadership prioritizing Project RED; RED adaptations; depth, accountability and influence of the implementation team; sustainability planning; and hospital culture. Only three of the five hospitals continued Project RED beyond the implementation period. The sustainability of RED in participating hospitals was only possible when hospitals approached RED implementation as a transformational process rather than a patient safety project, maintained a high level of fidelity to the RED protocol, and had leadership and an implementation team who embraced change and failure in the pursuit of better patient care and outcomes. Hospitals who were unsuccessful in implementing a sustainable RED process lacked all or most of these components in their approach.” |
| 1.Naldemirci  2. 2017 (27) | Person-centred care (PCC) framework | Not specifically stated but it is about delivering person-centred care | 1. PCC documentation  2. Three ‘routines’: collecting the patient’s narrative, establishing partnership by setting goals together and documenting this partnership.  “The PCC plan is developed with the patient and carers within 12-24 hours after admission or at the first outpatient attendance. This serves as a blueprint for ongoing care and treatment, with the patient’s goals set out alongside the routine medical information. Ongoing joint review results in care plans being changed over time to help tailor care to short term goals, such as the day for discharge, and longer-term ones, such as obtaining better sleep and reducing anxiety. The documentation of the PCC plan helps ensure continuity and provides transparency for all parties” (28) | 1. ‘Practitioners’ (“Practitioners took part in a 10-week PCC change management programme, dealing with the ethics of PCC and results from PCC studies. This includes training in developing tools such as care plans and interview techniques”). Lunch seminars, informal meetings, inter-professional discussions in small groups Invited lectures and seminars  2. Face-to-face; 1:1 and group  3. University hospital. Hospital wards varied in size, specialisation and patient group. | 1. NR but involved lunch seminars, informal meetings, inter-professional discussions in small groups, invited lectures and seminars (see Table 1).  2. Yes. Individualised for patient.  3. Yes. Consider deliberate strategies (before intervention initiating intervention and emergent strategies which “may help to explain the dynamic, often corrective and adaptive nature of implementation strategies. For complex interventions, there are often planned responses to expected problems. It is crucial to see how a set of practices are locally interpreted and modified in practice [12], which is often possible due to emergent strategies.  “ | “Emergent strategies to normalize PCC by (i) creating and sustaining coherence in small but continuously communicating groups (ii) interpreting PCC flexibly when it meets specific local situations and (iii) enforcing teamwork between professional groups. These strategies resulted in patients perceiving PCC as bringing about (i) a sense of ease (ii) appreciation of inter-professional congruity (ii) non-hierarchical communication” |
| 1. Nordmark  2. 2016 (29) | Discharge planning process (DPP) | To create work methods and ICT solutions that increased the accessibility, safety, quality, and efficiency of healthcare, lowering costs and creating regional growth.  Part of a larger project called Future’s Innovative Work Practices in Healthcare and Welfare (FIA). | 1. Phone, fax, mobile phones, computers, internet access, communication tool sent updated texts to patients and HCP  2. Five ICT solutions aiming to support the DPP; an electronic shared calendar, videoconference as a way of meeting for DPC, development of the electronic information system offering an attached file with the patient’s status in the request for DPC, follow-up of the agreed discharge plan, and a surveillance list to keep track of discharged patients’ in need of follow-up.  A specific electronic information system was developed and implemented to secure the information exchange between the hospital, primary healthcare and community care during the DPP. Patient-related information had to be doubly documented, first in the medical record system for internal use and then in the electronic information system for external use. | 1. Electronic system. The physician at the hospital ward was responsible for the DP, according to the law and the regulation. However, it was the RN at the hospital who performed the DP, from admission to discharge. The RN, DN, and HCO were central in the performance of the DPP.  2. Face-to-face (when possible), group, online  3. Five primary healthcare centres | 1. NR  2. Yes. Tailored for patients  3. Yes. (“DPC planning had improved because they started to use the electronic schedule consisting of a shared Microsoft Excel spread- sheet. This spreadsheet reduced meeting changes to nearly none, which saved time, but RNs believed that broad implementation countywide was needed”) | “Staff had reached a consensus of opinion of what the process was (coherence) and how they evaluated the process (reflexive monitoring). However, they had not reached a consensus of opinion of who performed the process (cognitive participation) and how it was performed (collective action). This could be interpreted as the process had not become normalized in daily practice. The result shows necessity to observe the implementation of old practices to better understand the needs of new ones before developing and implementing new practices or supportive tools within healthcare to reach the aim of development and to accomplish sustainable implementation” |
| 1. Parand  2. 2012 (30) | UK Safer Patients Initiative (SPI) | the aim of generating a sustained improvement in quality and patient safety by using continuous quality and process improvement techniques at 20 UK NHS organizations between 2006 and 2008 | 1. Videoconferencing equipment, computers, databases, internet access  2. Complex large scale multicomponent intervention (31). Focused on improving the reliability of specific processes of care where there were known strategies for improving safety.  Five core interventions:   - Leadership - Perioperative care - Medicines management - Critical care - General ward care   Interventions was designed to standardise care and reduce variation in practice, thus reducing the harm caused to patients from:   - clinical deterioration - ventilator associated pneumonia - central line bloodstream infections - MRSA bloodstream infections - anti-coagulation medication and - surgical site infections   They regularly brought together teams of 15-20 clinicians and leaders from each site, encouraging collaborative learning through face-to-face learning sessions, site visits and conference calls. Clinical improvement experts provided technical input and coaching to support teams in developing their improvement skills and implementing the interventions  Common measures and tools facilitated the sharing of data, information and learning across hospitals.  A ‘buddy’ model was later employed to encourage further collaboration to maximise achievements and the spread of learning. Concurrently, the initial four trusts from SPI 1 moved into a second phase, acting as ‘exemplar’ sites in order to help spread more widely their knowledge and learning on how to reduce adverse events.  Specific details about drivers and change package are described in Appendices p31-36 (31) | 1. ‘Collaborative’ methodology to support participating hospitals (31)  2. Face-to-face, groups, 1:1, outreach and conference calls  3. Four core hospital areas were the focus – see procedures for more detail. | 1. NR  2. Yes. For example, “SPI activity names were changed to everyday language” to allow for better local integration  3. NR | “three overarching factors for the sustainability of SPI: (i) using programme improvement methodology and measurement of its outcomes; (ii) organizational strategies to ensure sustainability and (iii) alignment of goals with external requirements. Within these were eight themes identified by the coordinators as helping to sustain the efforts of the SPI programme and its successes” |
| 1. Robert  2. 2011 (32) | The Productive Ward (PW) | National QI programme that aims to engage nursing staff implement change at ward level. Specifically, the programme aims to: increase the proportion of time nurses spend in direct patient care; improve experience for staff and patients; make structural changes to the use of ward spaces to improve efficiency in terms of time, effort and money | 1. Internet access and computer,  Online learning modules and toolkit are freely available to NHS organisations via the NHSI website. Hospitals also have the option of purchasing ‘Standard’ or ‘Accelerated’ packages from the NHSI to assist with local implementation. See <https://www.england.nhs.uk/improvement-hub/productives/>  2. Draws on Lean Thinking to reduce activities that don't add value (e.g. releasing more staff time for work that directly meets patient needs); involves modules and a toolkit to engage staff in the initiation and implementation of change at ward level; has packages to help implementation. The modules are “about ‘the how not the what’ and use a learning by doing approach which builds knowledge and skills to support frontline teams to make real and lasting improvements for themselves” (33) | 1. Online modules, some of the hospitals employed dedicated PW staff  2. Online  3. General ward. No other details available. | 1. Modules online. Not clear how long each takes to complete  2. Yes. “emphasising local ownership of the programme and empowerment of ward staff, rather than using a directive approach”  3. Unclear. | “Since the launch of the programme in May 2008 staff in approximately 85% of NHS acute hospitals had either downloaded Productive Ward materials or formally purchased a support package (as of March 2009). On a narrower measure, 40% (140) of all NHS hospitals had adopted the programme (i.e. purchased a support package) with large variation between geographical regions. Four key interactions in the diffusion of innovations framework appeared central to the rapid adoption of the programme. Despite widespread perception of significant benefits, frontline nursing staff report that more needs to be carried out to ensure that impact can be demonstrated in quantifiable terms and include patient perspectives” |
| 1. Rotteau  2. 2015 (34) | The Ontario ED process improvement program | To improve ED length of stay and improve patient flow | 1. Management tools and implementation tools. Details not provided.  2. Multicomponent: Systemwide interventions such as a pay- for-performance incentive program for eligible hospitals, funding for hospital-level interventions such as clinical decision units to manage short-stay patients, and a Lean-based QI initiative known as the ED Process Improvement Program. Key elements were:   - Ordering hospitals to report a common data set to the government monthly - •  Setting targets for length of stay (LOS) of ED patients based on triage acuity and disposition—the target for patients being admitted or complex patients (based on arrival acuity) being discharged home is 8 hours from arrival to departure to unit or home and for noncomplex (low‐acuity) patients is 4 hours from arrival to discharge - • Hospitals receive additional funds each year contingent on meeting hospital‐specific improvements in achieving targets. This is called pay for performance: the “money.” - • Hospital performance is publicly reported (the “shame”).   Other features include an expert panel to advise government on program planning, an intensive coaching program for hospitals to provide staff with information on best practices and to introduce them to “Lean” management techniques, and a major effort to improve hospital outflow by reducing the number of patients in hospital wards waiting for care in nonacute facilities (35).  The program provided one external lean coach to train and mentor improvement teams at each hospital, on-site support from lean management experts, training on the program methodology and tools for implementation, data management tools to track and report performance, linkages across sites to facilitate peer-to-peer mentoring, and forums for teaching and sharing progress among program sites. The program required that additional staff fill the positions of some team members while they were leading the program. Thus, in the second and third waves, to be eligible for the process improvement program, sites had to be participants in the pay-for-results program, which provided financial incentives to hospitals to improve ED waiting times (though not all hospitals in the pay-for-results program became process improvement program sites) (36) | 1. Dedicated hospital improvement teams, composed of senior leaders, managers, and staff from a variety of departments within participating hospitals, led the implementation of the Lean program at each hospital  2. Face-to-face, 1:1, group, online, published reports  3. The Ontario ED process improvement program was launched at the end of March 2009, with 5 hospitals in a single health region as the first wave. The second (beginning in November 2009) and third (May 2010) waves comprised 16 and 15 hospitals, respectively, from across Ontario (36) | 1. NR  2. Yes. Local context considered  3. Yes. “some modifications to the intervention delivery framework and training segments in subsequent waves. In addition, the external lean experts varied from wave to wave”.(36) | “four themes that were identified as significantly affecting the implementation experience: local contextual factors, relationship between improvement team and support players, staff engagement, and success and sustainability. The results demonstrate the importance of the context of implementation, establishing strong relationships and communication strategies, and preparing for implementation and sustainability prior to the start of the project” |
| 1. Sanchez  2. 2014 (37) | Medication reconciliation implementation | To improve medication reconciliation and reduce prescribing errors | 1. Medication list, computer, patient documentation  2. Medication reconciliation is the process of creating a best possible list of medications being used by a patient and comparing that list with the provider’s admission, transfer, and/or discharge orders. This occurs in 3 steps: verification (collecting the patient’s medication history), clarification (ensuring that the medications and doses are appropriate), and reconciliation (documenting changes in the orders) | 1. Implementation planning included physician managers, nurse managers, quality specialists, pharmacy managers, information technologists.  2. Face-to-face, 1:1 and online  3. Large urban academic tertiary care center and an affiliated Veterans Affairs (VA) hospital in New York City | 1. NR  2. Yes. Intervention tailored for patient.  3. NR | “Respondents described a resource- and time- intensive medication reconciliation planning process. The planning teams’ membership and functioning were recognized as important factors to a successful planning process. Implementation was facilitated by planners’ understanding of the principles of performance improvement, in particular, fitting the new process into the workflow of multiple disciplines. Nevertheless, a need for significant professional role changes was recognized. Staff training was recognized to be an important part of roll-out, but training had several limitations. Planners monitored compliance to help sustain the process, but acknowledged that this did not ensure that medication reconciliation actually achieved its primary goal of reducing error” |
| 1. Stacey  2. 2015 (38) | The CF lung transplant referral patient decision aid | To help adults with cystic fibrosis (CF) make a decision about lung transplantation | 1. Printed copies of the decision aid (English and French) http://decisionaid.ohri.ca/ decaids.html, one-page summary report, online tutorial, Implementation toolkit and workshop objectives are available at: https://decisionaid.ohri.ca/training.html  2. CF lung transplant referral patient decision aid based on the Ottawa Decision Support Framework and the International Patient Decision Aid Standards. Elements in the decision aid include focus on an explicit decision, best available evidence on treatment options for end-stage CF lung disease (transplant versus supportive care), probabilities of benefits and risks, an explicit values-clarification exercise, and structured guidance in making the decision. This patient decision aid includes a one-page summary report to facilitate sharing the patients’ knowledge, values, and preferences with HCPs. This one-page report can be filed on the patients’ health record. A 5 hour workshop was provided to address these and increase knowledge and skills. the Ottawa Decision Support tutorial was provided. Printed copies of the patient decision aid in English and French were provided. Conference calls were used to reinforce learning and provide ongoing support. | 1. Unclear.  2. Face-to-face; 1:1, group, TC, online  3. CF clinics | 1. 5-hour workshop, 1-hour presentation, Calls occurred every 3 months in the first year and every 6 months in the second year  2. Yes. intervention was tailored on the basis of healthcare professionals' perceived barriers to implementation.  3. Unclear | “Of 23 adult CF clinics, 18 participated (78.2%) and 13 had healthcare professionals attend training. Baseline barriers were healthcare professionals’ inadequate knowledge for supporting patients making decisions (55%), clarifying patients’ values for outcomes of options (58%), and helping patients handle conflicting views of others (71%). Other barriers were lack of time (52%) and needing to change how transplantation is discussed (42%). Baseline facilitators were healthcare professionals feeling comfortable discussing bad transplantation outcomes (74%), agreeing the decision aid would be easy to experiment with (71%) and use in the CF clinic (87%), and agreeing that using the decision aid would not require reorganization of the CF clinic (90%). After implementing the decision aid with interventions tailored to the barriers, decision aid use increased from 29% at baseline to 85% during year 1 and 92% in year 2 (p < 0.001). Compared to baseline, more healthcare professionals at the end of the study were confident in supporting decision-making (p = 0.03) but continued to feel inadequate ability with supporting patients to handle conflicting views (p = 0.01)” |
| 1. White  2. 2011 (39) | Methods to optimise medication reconciliation | To increase and sustain completion of medication reconciliation at admission to greater than 90% for inpatient medical services. | 1. Computers, reconciliation tools, educational programs, labels  2. Multicomponent intervention (outlined in Figure 2) focused on five main areas: leadership and support from senior physicians and nurses to sustain a culture of safety; simplification and standardisation of the electronic medication reconciliation application; clarifying roles and responsibilities; creating a highly reliable and visible system; and sustainability.  Specifically -   - Identify senior physicians and nursing leaders as process owners - Electronic medication reconciliation tool enhancements, integration of medication reconciliation with clinical order entry and discharge summary - Post physician and nurse performance on respective units/ divisions; develop a group email list to communicate staff performance - Identify and mitigate during rounds and build in reminders – labels on laptops - Rearrange updater admission process for all admits - Updated/revised education programs for staff; new resident training, roll out unit level education with clinical systems improvement, plus inpatient, practice council, education council support. | 1. Nurses and doctors on ward; the improvement team, consisted of physician and nursing leaders, frontline nurses and physicians, a quality-improvement consultant, a data analyst, and a patient safety project manager  2. Face-to-face, 1:1, group and email  3. Large urban paediatric medical centre | 1. Reconciliation was defined as recording a complete and accurate list of each patient’s medications within 20 min of admission by the nurse and reconciliation of those medications within 24 h of admission by the physician.  2. Yes. Tailored to patient needs  3. NR | “At baseline, only 62% of patients had their medications reconciled within 24 h of admission. Over a 9-month period, >/=90% medication reconciliation was achieved within 24 h of admission. These results have been sustained for 27 months” |

**References**

1. Ament SMC, Gillissen F, Moser A, Maessen JMC, Dirksen CD, von Meyenfeldt MF, et al. Factors associated with sustainability of 2 quality improvement programs after achieving early implementation success. A qualitative case study. J Eval Clin Pract. 2017;23(6):1135-43.

2. Belizan M, Bergh AM, Cilliers C, Pattinson RC, Voce A, Synergy G. Stages of change: A qualitative study on the implementation of a perinatal audit programme in South Africa. BMC Health Serv Res. 2011;11:243.

3. Bergh AM, Kerber K, Abwao S, de-Graft Johnson J, Aliganyira P, Davy K, et al. Implementing facility-based kangaroo mother care services: lessons from a multi-country study in Africa. BMC Health Serv Res. 2014;14:293.

4. Bernstein E, Topp D, Shaw E, Girard C, Pressman K, Woolcock E, et al. A preliminary report of knowledge translation: lessons from taking screening and brief intervention techniques from the research setting into regional systems of care. Acad Emerg Med. 2009;16(11):1225-33.

5. Bhanbhro S, Gee M, Cook S, Marston L, Lean M, Killaspy H. Recovery-based staff training intervention within mental health rehabilitation units: a two-stage analysis using realistic evaluation principles and framework approach. BMC Psychiatry. 2016;16:292.

6. Cook S, Mundy T, Killaspy H, Taylor D, Freeman L, Craig T, et al. Development of a staff training intervention for inpatient mental health rehabilitation units to increase service users’ engagement in activities. British Journal of Occupational Therapy. 2015;79(3):144-52.

7. Bouamrane MM, Mair FS. Implementation of an integrated preoperative care pathway and regional electronic clinical portal for preoperative assessment. BMC Med Inform Decis Mak. 2014;14:93.

8. Brady PW, Brinkman WB, Simmons JM, Yau C, White CM, Kirkendall ES, et al. Oral antibiotics at discharge for children with acute osteomyelitis: a rapid cycle improvement project. BMJ Qual Saf. 2014;23(6):499-507.

9. Bridges J, May C, Fuller A, Griffiths P, Wigley W, Gould L, et al. Optimising impact and sustainability: a qualitative process evaluation of a complex intervention targeted at compassionate care. BMJ Qual Saf. 2017;26(12):970-7.

10. Campbell S, Pieters K, Mullen KA, Reece R, Reid RD. Examining sustainability in a hospital setting: case of smoking cessation. Implement Sci. 2011;6:108.

11. Fleiszer AR, Semenic SE, Ritchie JA, Richer MC, Denis JL. An organizational perspective on the long-term sustainability of a nursing best practice guidelines program: a case study. BMC Health Serv Res. 2015;15:535.

12. Fleiszer AR, Semenic SE, Ritchie JA, Richer MC, Denis JL. A unit-level perspective on the long-term sustainability of a nursing best practice guidelines program: An embedded multiple case study. Int J Nurs Stud. 2016;53:204-18.

13. Frykman M, von Thiele Schwarz U, Muntlin Athlin A, Hasson H, Mazzocato P. The work is never ending: uncovering teamwork sustainability using realistic evaluation. J Health Organ Manag. 2017;31(1):64-81.

14. Glasgow JM, Yano EM, Kaboli PJ. Impacts of organizational context on quality improvement. Am J Med Qual. 2013;28(3):196-205.

15. Glasgow JM, Davies ML, Kaboli PJ. Findings from a national improvement collaborative: are improvements sustained? BMJ Qual Saf. 2012;21(8):663-9.

16. Gould DJ, Hale R, Waters E, Allen D. Promoting health workers' ownership of infection prevention and control: using Normalization Process Theory as an interpretive framework. J Hosp Infect. 2016;94(4):373-80.

17. Gramlich LM, Sheppard CE, Wasylak T, Gilmour LE, Ljungqvist O, Basualdo-Hammond C, et al. Implementation of Enhanced Recovery After Surgery: a strategy to transform surgical care across a health system. Implement Sci. 2017;12(1):67.

18. Green SA, Bell D, Mays N. Identification of factors that support successful implementation of care bundles in the acute medical setting: a qualitative study. BMC Health Serv Res. 2017;17(1):120.

19. Hommel A, Gunningberg L, Idvall E, Baath C. Successful factors to prevent pressure ulcers - an interview study. J Clin Nurs. 2017;26(1-2):182-9.

20. Hovlid EB, O.;Haug, K.;Aslaksen, A. B.;von Plessen, C. Sustainability of healthcare improvement: what can we learn from learning theory? BMC health services research. 2012;12:235.

21. Ilott I, Gerrish K, Eltringham SA, Taylor C, Pownall S. Exploring factors that influence the spread and sustainability of a dysphagia innovation: an instrumental case study. BMC Health Serv Res. 2016;16(1):406.

22. Jangland E, Gunningberg L. Improving patient participation in a challenging context: a 2-year evaluation study of an implementation project. J Nurs Manag. 2017;25(4):266-75.

23. Matthew-Maich N, Ploeg J, Dobbins M, Jack S. Supporting the Uptake of Nursing Guidelines: what you really need to know to move nursing guidelines into practice. Worldviews Evid Based Nurs. 2013;10(2):104-15.

24. Mazzocato PH, R. J.;Brommels, M.;Aronsson, H.;Backman, U.;Elg, M.;Thor, J. How does lean work in emergency care? A case study of a lean-inspired intervention at the Astrid Lindgren Children's hospital, Stockholm, Sweden. BMC health services research. 2012;12:28.

25. McClung L. Health care worker perspectives of their motivation to reduce hospital-acquired infections. Journal of Investigative Medicine. 2017;65(4):824.

26. Mitchell SE, Weigel GM, Laurens V, Martin J, Jack BW. Implementation and adaptation of the Re-Engineered Discharge (RED) in five California hospitals: a qualitative research study. BMC Health Serv Res. 2017;17(1):291.

27. Naldemirci O, Wolf A, Elam M, Lydahl D, Moore L, Britten N. Deliberate and emergent strategies for implementing person-centred care: a qualitative interview study with researchers, professionals and patients. BMC Health Serv Res. 2017;17(1):527.

28. Ekman I, Hedman H, Swedberg K, Wallengren C. Commentary: Swedish initiative on person centred care. BMJ. 2015;350:h160.

29. Nordmark S, Zingmark K, Lindberg I. Process evaluation of discharge planning implementation in healthcare using normalization process theory. BMC Med Inform Decis Mak. 2016;16:48.

30. Parand A, Benn J, Burnett S, Pinto A, Vincent C. Strategies for sustaining a quality improvement collaborative and its patient safety gains. Int J Qual Health Care. 2012;24(4):380-90.

31. Foundation. H. Learning report: Safer Patients Initiative. UK: The Health Foundation; 2011.

32. Robert G, Morrow E, Maben J, Griffiths P, Callard L. The adoption, local implementation and assimilation into routine nursing practice of a national quality improvement programme: the Productive Ward in England. J Clin Nurs. 2011;20(7-8):1196-207.

33. NNRU. National Nursing Research Unit and the NHS Institute for Innovation and Improvement. The productive ward: releasing time to care. Learning and impact review. Warwick: NHS Institute for Innovation and Improvement; 2010.

34. Rotteau L, Webster F, Salkeld E, Hellings C, Guttmann A, Vermeulen MJ, et al. Ontario's emergency department process improvement program: the experience of implementation. Acad Emerg Med. 2015;22(6):720-9.

35. Ovens H. ED overcrowding: the Ontario approach. Acad Emerg Med. 2011;18(12):1242-5.

36. Vermeulen MJ, Stukel TA, Guttmann A, Rowe BH, Zwarenstein M, Golden B, et al. Evaluation of an emergency department lean process improvement program to reduce length of stay. Ann Emerg Med. 2014;64(5):427-38.

37. Sanchez SH, Sethi SS, Santos SL, Boockvar K. Implementing medication reconciliation from the planner's perspective: a qualitative study. BMC Health Serv Res. 2014;14:290.

38. Stacey D, Vandemheen KL, Hennessey R, Gooyers T, Gaudet E, Mallick R, et al. Implementation of a cystic fibrosis lung transplant referral patient decision aid in routine clinical practice: an observational study. Implement Sci. 2015;10(1):17.

39. White CM, Schoettker PJ, Conway PH, Geiser M, Olivea J, Pruett R, et al. Utilising improvement science methods to optimise medication reconciliation. BMJ Qual Saf. 2011;20(4):372-80.
