# Supplementary material for: The barriers and facilitators influencing the sustainability of hospital-based interventions: a systematic review
Source: BMC Health Serv Res. 2020 Jun 28;20:588. doi: 10.1186/s12913-020-05434-9 (PMC7321537; doi:10.1186/s12913-020-05434-9)
Supplement: Supplementary file 5 — Additional file 5. Methodological quality assessment for qualitative studies. [file 12913_2020_5434_MOESM5_ESM.docx]

**ADDITIONAL FILE 5. METHODOLOGICAL QUALITY ASSESSMENT FOR QUALITATIVE STUDIES**

Colour code key: Green = yes; Orange = can’t tell; Red = no.

Abbreviations: AEA: Appropriate ethical approval, EC: Ethically Conducted, FA: Framework Analysis, NCI: No competing interests, PS: Purposive sampling, QCS: Qualitative Case Study, QS: Qualitative study, RE: Realist evaluation, RPR: Researcher participants relationship. #: number

| **First author (Year)** | **CASP 1: Clear aim?** | **CASP 2: Appropriate methodology?** | **CASP 3: Appropriate design?** | **CASP 4: Appropriate recruitment?** | **CASP 5: Appropriate data collection?** | **CASP 6: Relationship researcher-participants?** | **CASP 7: Ethical issues considered?** | **CASP 8: Rigorous data analysis?** | **CASP 9: Clear findings?** | **CASP 10: How valuable is the research?** |
| --- | --- | --- | --- | --- | --- | --- | --- | --- | --- | --- |
| Ament (2017) (1) | Intro.,para 3 |  | Indirectly -refer to protocol published elsewhere (p1136) | PS, snowball sampling (para 2) (noting potential limitation due to selection bias). | Para: 'Data collection' comprehensive. Performance data used in interviews to probe discussion (noted as study strength).  ‘Data analysis’ (2.5) discusses data saturation. | Author info and contributions provided.  Participants’ details limited due to guarantee of anonymity (incl. as a limitation).  RPR unclear. | No competing interests. AEA, EC.  Transparent reporting of decisions throughout. | 'Data analysis' (2.5) and 'Trustworthiness' (2.6) | ‘Trustworthiness' (2.6). 'Results' (3) organised by CFIR factor and those not fitting reported, and summarised | Findings compared/contrasted with other studies (e.g. determinants of sustainability different from those of implementation) |
| Belizan (2011) (2) | Audit important.  Low/middle income countries have little on implementation, facilitators/barriers, sustainability. This study aims to address this. | QS: explore clinicians' experiences of initiation, implementation and maintenance | Decisions guided by those with direct experience of implementing and sustaining. | Well-documented PS, invited most experienced in implementing and sustaining to participate.  Analysis focused on 'insider' knowledge even though some with 'outsider' knowledge took part.  # invitees/participants not stated.  # sites reported. | ‘Methods’: Workshop format and data collection well described.  Rationale for mixing participants (session 2) not stated. | Researchers' own role / reflexivity not discussed explicitly. Research does focus on most experienced and allows for contextual variation.  Transparency around changes in study design. | AEA, EC.  Unclear why participants' managers had to sign forms giving permission to attend. | 'Methods' /'Analytical framework'. 'Discussion:' limited # of sites able to collect data for 5+ years. | 'Results', (i) four inter-related themes, then (ii) discussion on stages of change. | Discussion: Findings compared with other studies. Transferability discussed.  Findings translated into practical tool.  . |
| Bhanbhro (2016) (3) | Explore factors associated with variation in sustainability across units of recovery-oriented practice  during the recovery-focused staff training intervention (GetREAL)" | RE, QS, theory testing and refinement to ask for whom and in what circumstances was the intervention 'reinforced' (sustained) | QCS blending RE and FA. | Sample limited by time/resources.  4- unit section criteria outlined.  Case study and remaining 3 units discussed in detail (table 3). | Data not collected for the purpose of this evaluation. ‘Data collection’ section comprehensive.  . | RPR – researcher overlap with evaluation, design of RCT from which data drawn.  Support of participants acknowledged.  RPR not discussed in detail.  . | AEA, EC.  Dataset available from corresponding author (not shared due to anonymity/confidentiality constraints) | ‘Data analysis’ detailed, decisions justified, processes documented.  Reliability/validity discussed. | 4 themes linked to maintenance/inhibition of long-term change.  7 candidate theories tested.  . | Comprehensive 'Discussion' |
| Bouamrane and Mair (2014) (4) | Aim: to inform future implementation of standard pre-operative nurse-led assessment clinics supported by a pre-operative electronic form by identifying complex sociotechnical factors involved in successful adoption | Reflects messy reality of aim and NHS | Pragmatic, purposive decision-making, integration of policy, theory, and literature. | Limitation: #interviews small, however qual/quant of data produced high, and interviewees regarded as highly purposive and successful. | Pragmatic data collection reflecting distributed and iterative nature of the implementation. | Not explicitly discussed, other than to say the researcher was "invited" to attend meetings which were used as part of the analysis. | AEA, EC for initial meetings however, consent not discussed for 3^rd^ meeting. Ethics not otherwise discussed. | 'Data analysis': describes process. | 'Results': 2 themes emerging from the data but also form 2 of research aims (incl. design process and implementation). Case study presented.  Discussion: factors facilitating successful implementation using NPT.  Conclusion: key factors. | Clear discussion of policy/practice context and use of NPT.  Identify impact.  Discussion of future research with larger sample. |
| Bridges (2017) (5) | Draws on NPT implementing an intervention supporting the delivery of compassionate care by hospital teams" | Experience of implementation over time | Introduction and methods: well- argued case. | PS.  Longitudinal approach.  Detail given of numbers, roles and # interviews (taking place, declined). Recruitment: ward level. | ‘Methods’: Interviews, field notes, observations, staffing data via q’nnaire. | Different roles of NHS / university researchers in research activities are indicated, but no other discussion. | Short description of AEA, otherwise not discussed. | ‘Methods’: purpose to gather multiple perspective and elicit complex and situated understanding. | Individual elements implemented but sustainability difficult to achieve for some wards (explanation provided in ‘findings’. | Covered comprehensively in 'Discussion' |
| Campbell (2011) (6) | Understand how hospitals were sustaining OMSC. | Sought to understand approach taken to sustainability. | PS of sites and interviews. | Constrained by budget and time: 2 informants interviewed per hospital via PS.  ‘Data collection’ describes recruitment.  Details of #recruited /declined/ reasons. | Interviews developed with advisory group. Interviews audio recorded. Range: (16-59 minute interviews via phone). | ‘Limitations’: possible social desirability bias, impact of interviewer not being member of staff at U of Ottawa Heart Institute (funders). | AEA. | ‘Analysis': description of transcription/coding/consensus/comparative table /use of Gruen et al. model. | ‘Results' : hospital participation, sustainability and interaction themes (Gruen et al. model), the latter with supporting quotes. | Clear where differences/similarities in sustainability occurred.  ‘Conclusion’: importance of interactions occurring in hospital context. ‘Discussion’ how relates to other literature. |
| Fleiszer (2016) (7) | Understand how a nursing program sustained in an acute healthcare centre. | QS. | QCS with embedded comparative case studies. | PS of units and individuals. | Interviews, framework-guided, audio recorded.  Justification for estimate of numbers, interview guide, location. Includes additional data sources. | Not reported other than Table 2 (credibility, trusting relationship, confirmability) | Table 2 (EC, AEA) | Table 2 (rigour) 2.4: detail about sources, consistency, tactics, corroboration. Also detail on process of coding, composing, condensing, writing, techniques/objectives. | 3: Results in sections that match determinant framework. Presented in figure 1 graphically | 4.1: Methodological strengths and limitations, 4.2 Theory and research implications (including Table 5 parallels with NPT) and 4.3 Practice implications. |
| Fleiszer (2015) (8) | How nursing best practice guideline programme sustained in acute healthcare centre. | QCS | Interviews, documents, observation. Limitation of going for depth at expense of breadth acknowledged, as is retrospective rather than prospective focus. | Key informants (nurses with department leadership positions). PS.  All agreed to participate. | Data sources and characteristics' section. 14 individual semi-structured, framework-guided interviews, observation and documentary analysis. | Not reported, other than that process for researchers included staying close to participant and "writing reflective memos". | AEA, EC. Limitations on anonymity reported. | 'Data analyses' ,'Strategies for study rigor'. Discussion on frequency, “redundancy” for stopping.  Documents/observation use to enhance data.  Reported adherence to RATS guidelines. | Organised by 3 research questions and framework; 3 characteristics of innovation sustainability, 4 categories of factors (containing 11 factors). Also presented graphically. Informant quotations in Table 2. | Discussion of conceptual framework , relationship to previous research, methodological strengths and limitations, implications for theory and research and implications for practice. |
| Frykman (2017) (9) | Uncover mechanisms influencing sustained behaviour change following implementation of teamwork at an ED. | QS | RE | ‘Phases 3 and 4’: selection criteria. No indication that anyone declined. | Data collection and analysis' section, Table 1 (overview) and Figure 2 (timeline of stages/data collection.  Included observation, documents, interviews to generate a program theory, and realist interview approach, all well described. | 2^nd^/3^rd^/last author are original research team. 3^rd^ author also RN with ED experience and was employed in the organization (though not in clinic) | APA (p.70), EC (discussion of confidentiality of data provided) | Analysis ongoing (in-line with RE). Analysis of transcripts provided, inc. decision to analyse independently, assess agreement, then reach consensus through discussion (p.69). | Results (p.70) presented as 5 refined CIMOs (Context, Intervention, Mechanism, Outcome (p.71). A strength is the comparison with teamwork at the implementation stage. | Discussion (p.74) refers to DCOM dimensions to explain the low sustainability and relate this to other previous research. Also 'Methodological considerations' includes discussion of credibility, limitations and transferability. |
| Gould (2016) (10) | Explore meaning of IPC ownership to health workers, evaluate impact of an action plan to encourage IPC ownership across NHS health boards in Wales. | QS | Independent, retrospective evaluation using NPT | PS.  Maximum variability for different types of staff (occupational group and seniority) 'Findings' sampling info. incl. role, male/female, minimum length of NHS employment, were no refusals. | ‘Sampling and study procedures': use and content of topic guide.  Data collection ceased when saturation reached. 20 informants, ~1hr interview, recorded, transcribed. | Independent evaluation - but implications and detail of the relationship not discussed | AEA. | 'Sampling and study procedures’: Inductive analysis.  'Theoretical framework': rationale for framework and use of NPT. | Described with 4 NPT constructs. 'Facilitators and inhibitors to taking ownership' and outcome measures of success. Sections also related to the 6themes derived inductively. | Discussion is short; links with similar studies and contracts with earlier research.  Discusses retrospective use of NPT, sampling implications. Conclusions: include frontline as well as senior staff in future. |
| Gramlich (2017) (11) | What are the barriers and enablers to ERAS implementation within a health care system? | Identifying barriers and facilitators to implementation in social contexts | Comprehensive use of frameworks to thematically analyse a variety of qualitative data sources | Data came from 6 participating hospitals in 1 health system: 2 early adopter (mid-2013); 4 additional (2014). Active implementation phase 9-12 months. Data analysed after all sites implemented intervention for >=12months. | Multiple qualitative data sources Patient, provider and system level included (no. participants / roles detailed). | Not discussed, but roles in project and healthcare are described in 'Authors' contributions'. | AEA. Describe how quality improvement does not need ethics approval and consent from patients. Approval for participation in focus groups, survey and interviews via University of Alberta. | It is difficult to follow exactly what was done due to the variety of data sources and frameworks, but it seems rigorous and there are no red flags. | 'Results' are presented under two sub-headings: 'Context - implementation, impact on outcome' and 'Knowledge translation data analysis'. | ‘Discussion' : contribution of an end-to-end implementation report using theoretical frameworks and data to learn from and guide practical action over time in context. Limitations are discussed. |
| Green (2017) (12) | Identify factors that support successful implementation of 2 care bundles in acute medical settings that used QI methods. | QS | 2 care bundle implementation initiatives (as case studies) supported by a CLAHRC | Progress reviews by implementation teams "intended to be opportunities for each team to reflect on implementation and progress to date." | Data was review reports, review minutes and audio recordings of review meetings where available (Table 2). | Competing interests declared (e.g. main author was member of implementation team as a tech. QI advisor on behalf of the funder, but implications not discussed in main text. | AEA, EC. | ‘Analytical approach': rational for choice of CFIR and partial application outlined. Used to create a coding framework, largely deductive but allowing for other themes. | Structured according to included domains with supporting quotes. Discussion identifies four key factors. | Discussion links to other research / policy, and Strengths and limitations are acknowledged. |
| Hommel (2016) (13) | Explore successful factors to prevent pressure ulcers in hospital settings | ~QS | Interviews, focus groups. | Clear criteria for sampling settings BUT reader is left to assume the change in prevalence was positive. Patient safety facilitator at hospital "identified respondents that could participate" - no info. potential sample/mix of participants, although the 39 participants came from 4 groups (managers, physicians, registered nurses, enrolled nurses). | Interviews + focus groups but no rationale about decision on mode of participation.  'Data collection': reasonably comprehensive. It is unusual to have a second researcher in an interview; the potential impact of this is not discussed. | All researchers conducted the analyses, who are all researchers within the area of PU; this could influence the interpretation of the text. | The principles set out in Declaration of Helsinki, as well as national and local ethical guidelines for research, were followed. EC. | 'Data analysis' describes process briefly. How PARIHS was integrated is not clear. Quotes are unattributed to profession or hospital type. | Findings clearly presented.  Case made for sampling/data collection allowing for triangulation.  However, the style of writing means the findings are presented in a general way. | Section on contribution to global clinical community. The Discussion relates findings to other literature and makes recommendations. Potential transferability is discussed. |
| Hovlid (2012) (14) | Use of an organizational learning framework to explore a case that demonstrated sustained improvement. | QS | ‘Design’: Given the scarce knowledge on sustainability of healthcare improvements, the character of our study is explorative. QCS, grounded in theoretical framework of learning theory. | Selected case is "an example of sustained improvement through organizational learning". Projects were multi-professional. PS of individuals.  No info. given about # individuals approached / declined. | Administrative documents used to provide background (no formal documentary analysis). 'Data collection': interviewees, number, length, case notes. | Not discussed. | ‘Ethical considerations': explains why full ethical review deemed unnecessary, and who had oversight of collecting and handling data (incl. consent, anonymity) | ‘Analysis': outlines process and who was involved at each stage. Fig. 1: Relationship between themes | ‘Results': structured around the 4 main themes of the ELO model, with quotes. 'Discussion' findings in relation to the theoretical framework. | Sections in Discussion 'Our findings in relation to earlier studies' and 'Implications for quality improvement in health care' (also in Table 2) and 'Limitations, relevance and further research'. |
| Illot (2016) (15) | To track the spread and sustainability of a locally developed dysphagia recommendation over time (April 2011 to January 2014) at organisational and clinical levels. | Prospective, longitudinal, theoretically driven instrumental case study. | Individual and group interviews and document review. | PS of interviewees according to roles/clinical levels.  Table 3: demographic details. | Interview topics, interviewers, mean duration, recording method described. Reviewed documents described. | Researchers are employees of the organisation – provided insider knowledge, facilitated collaboration and trust.  The knowledge broker role was a potential source of bias due to the researchers' commitment to dysphagia as a patient safety issue.  Bias was addressed through reflexivity, by acknowledging the dilemmas when moving between the 'insider' and 'outsider' researcher positions, through discussion and in the field notes." | AEA, EC. | Analysis' section is comprehensive. | Complex analysis - would have been helpful to have more information introducing why the findings were presented this way. 'Results': lays out the processual approach under 5 headings related to the Buchanan theory. These are integrated throughout with the spread theory. A final section looks at 'Outcomes and impact'. | ‘Discussion': provision of generalizable insights.  Comprehensive exploration of strengths, limitations, and generalisability. |
| Matthew-Maich (2013) (16) | Aim: generate a grounded theory of the processes that support the implementation and uptake of BPGs in nursing practice | Background, Discussion: Rationale behind using grounded theory | 'Background' and 'Design'. | Comprehensively described and justified in 'Research Setting and Participant Recruitment' and 'Sampling'. Success of recruitment strategy reported clearly in ‘Findings: Sites and Participants' and Table 1. | Comprehensively described and justified in 'Data Collection' and 'Rigour'. | The researchers are largely absent from the text, although they are referred to as "Three experienced qualitative researchers" | ‘Ethics section': AEA, EC.  Informed consent: written for face-to-face, verbal for phone, reason for difference not explained. Voluntary participation, withdrawal covered. The $20 gift certificate for participants declared but not discussed / explained. | ‘Data Analysis': comprehensive and consistent with grounded theory. Supporting quotes used. | SUNG framework (grounded theory) is presented (fig. 1). Visual representation in the findings. The 2 uptake sites are compared/contrasted with the minimal uptake site. | Thorough exploration in 'DISCUSSION' of what the SUNG framework has in common with existing knowledge translation theories, and where it complements them. 'Limitations and Strengths' and 'Implications for Practice, Education and Research' are comprehensive. |
| McClung (2017) (17) | Examine health care worker motivation for reducing hospital acquired infection - but also (under 'Study design) "perspectives of facilitators and barriers of HAI prevention bundle implementation." | Phenomenological QS | Assumption that qualitative methods are best to meet aim. | One hospital. Wide range of professions involved in implementation of HAI prevention.  Recruited by email, then "a snowball sampling strategy was used". 10 interviewed/5 further did not respond.  Table 1 shows timeline. | ‘Data collection and analysis': Brief description of interviews.  ‘Respondents’: Interviewed until theoretical saturation.  ‘Results’  'Results' : focus groups chosen to mitigate social desirability bias. | Not discussed. | Short statement that study is except from ethics approval because it is QI. | Very brief section in 'Data collection and analysis' - multiple reading of transcripts to identify concepts / themes not already in CFIR, then coding to model plus "any novel themes". However, it is not made clear what these were. | Laid out as CFIR constructs, with Table 2 summarising with exemplar quotes | Relevant but light touch study (due to small sample size from single institution, reliance on self-report, and self-selection bias all introduced limitations). Discussion briefly relates findings to other literature and makes recommendations based on findings. |
| Mitchell (2017) (18) | Contextual factors influencing the decision making process and motivations behind adaptations of the RED protocol and the impact on sustainment of RED. | QS | Qualitative appropriate for contextual factors and decision making | 64 individuals involved, roles / numbers in Table 2. Included RED implementers at different levels, non-RED staff, and community partners. Limitations specified (incl. not all desired interviews took place). | Interview guides available.  64 individuals involved in 1:1 interviews / focus groups. Researchers also shadowed RED providers to assess fidelity of implementation. Limited detail. 3/5 sustainably integrated RED, allowing for comparison. | Although state "All of these sites received Project RED training by the BMC RED research team prior to their implementation of RED" there is no discussion about the implications of this for the study. | AEA. EC. Written informed consent. "All hospitals compensated $750 for their involvement in the study." | ‘Data analysis': modified grounded theory approach (Fig. 2). Includes key defns. | ‘Results' presented as sections in line with conceptual model of components needed for sustainable implementation (Figure 1). Quotes included. | 5 components identified amongst sites sustaining RED. These discussed in detail.  Limitations discussed. |
| Naldermici (2017) (19) | 1) Explore the deliberate and emergent strategies of key stakeholders to specific contextual challenges encountered when implementing the GPCC framework; 2) explore how the recipients of PCC perceived the effects of these strategies. | QS | Discussed in Background. 'Sample and design': describe what they did but not what they could have done instead. ‘Discussion’combines 3 stakeholder groups’ perspectives on the implementation of a specific PCC framework". | 'Sample and design': Different sampling strategies used for each group, and numbers / roles / circumstances provided. Eligibility criteria discussed. | ‘Data collection': interview description comprehensive. | Not reported. | 'Data collection' - pseudonyms, information, consent, approval. Also in 'Sample and design', ward manager consent and role (although implications of their dual management / recruitment role not discussed). | ‘Data analysis' describes process, and use of NPT. | ‘Results' : barriers to normalization (using 4 NPT mechanisms) and emergent strategies of responding to them (Mintzberg and Water 1985) based on analysis of researcher and practitioner data (summary Table 1). Explanation of how effect of strategies was experienced by patients. | Main findings discussed in relation to literature. Limitations offers ("interpretive and not exhaustive", "points to some possible directions for future research" and "may not be transferable". Strong 'Conclusions'. |
| Nordmark (2016) (20) | Explore the embedding and integration of the DPP from the perspective of registered nurses, district nurses and homecare organizers." | Exploration of perspectives | Secondary analysis of data from different perspectives, plus additional relevant but previously unanalysed data | Set within context of larger project. Participant settings included 5 hospital wards with highest frequency of discharge planning, and primary healthcare centres selected purposively. Info given on professionals who agreed to participate, but not those who potentially could have or did not. | Variety of documentary sources from 2009-2010 - workshops, survey, interviews. | Role of first author presented as both a strength and limitation (p.8 of 10) as both informal project leader and researcher. Individuals informed as to role of this individual during the study. | AEA, EC. | Data analysis' process, including integration with NPT, described. | Results presented under NPT framework (4 constructs). | ‘Discussion' summarises findings and how the use of NPT uncovered implementation issues not recognised in In previous reflections, and how they guarded against bias in the analysis. Some reference to literature. |
| Parand (2012) (21) | Examination of strategies to promote sustainability of an organizational safety improvement programme | QS appropriate to gather information about strategies | Careful thought given to when interviews conducted and rationale provided. | PS (details provided in Table 1) . No mention of how many approached / refused. | 2 purposively selected time points. Numbers, type, times of interviews stated. Eg questions Five interviewers. | No information provided, although funding for the research was provided by one of the same organisations (Health Foundation) which initially funded the programme. | Brief mention regarding EC (information sheet, anonymity discussion, consent form) | ‘Data analysis' : process of and people involved in transcription and analysis leading to model of factors and sub-factors. Multi-disciplinary team for analysis - credentials presented. | Factors and sub-factors presented (Table 3) and narratively with quotes. Additional 'case study examples' (Table 4). | ‘Discussion' : 3 main factors and related to other research. Limitations detailed. Strength of longitudinal approach for studying sustainability. |
| Rotteau (2015) (22) | Describe experiences during program implementation the implementation team’s perceptions of factors influencing success/failure | QS | Descriptive QS. Selection: 3 hospitals and 2 hospitals with largest/least improvement respectively. Limitation: cannot compare hospitals or identify factors present in successful but not unsuccessful hospitals. | Maximum variation sampling, aim 5 respondents per hospital with a wide range of perspectives.  Table 1: Characteristics of hospitals and participants (table 1 and 2).  Respondents were the implementation teams. Nothing on how recruitment accomplished. | Rationale given for interview time scale.  Topic guides based on review of QI literature and Ministry which launched the strategy.  Brief information given about interview guides. | Not discussed. | AEA. EC. | ‘Study protocol': discusses interview process and use of thematic analysis.  'Data Analysis': discusses numbers, coding, consensus reaching. | Organised by participants' experience of the stages of the projects, grouped in to 4 themes. Also shown in Tables 3-6. | ‘Discussion’, ‘Limitations’: link to previous research. ‘Conclusions’: 5 key factors.  . |
| Sanchez (2014) (23) | Factors affecting implementation of medicine reconciliation from the perspectives of those involved in planning it. | QS appropriate to explore perspectives on implementation at two hospital where implementation had been in place for 3 years | Description of a ‘grounded theory approach' not convincing for what was actually done in the study. | Target group clearly described but snowball sampling description was used "until there were no further individuals involved in the medication reconciliation planning process to interview", but not clear how they knew this. 14 invited for interview, 1 not available. Time of involvement, roles in reconciliation and profession of interviewees reported. 2 hospitals included, but no info. given about why 2, or why these 2, or why they differed. | Rationale for use of interviews provided. Topic guide reported. Interview time 30-60 minutes. | Study limitation: “that one author (KSB) was a member of the participating organizations during medicine reconciliation planning and implementation, which may have influenced data collection and interpretation." Strength: same settings as their previous research so could build on this experience.  2 researchers per interview:  rational given but implications for respondents not discussed. | AEA, EC. | 'Data analysis': coherent approach is not clear (e.g. "Codes were derived from a combination inductive approach using a constant comparison method" - but what is a "combination inductive approach"?)  Although put forward as grounded theory, themes were listed and labelled from CFIR constructs. | Results presented as 5 themes, each related to a CFIR construct. | Related to PDSA model as well as CFIR.  Unanticipated findings included: (1) need for effectiveness data identified; (2) patient experience inadequately considered. |

**References**

1. Ament SMC, Gillissen F, Moser A, Maessen JMC, Dirksen CD, von Meyenfeldt MF, et al. Factors associated with sustainability of 2 quality improvement programs after achieving early implementation success. A qualitative case study. J Eval Clin Pract. 2017;23(6):1135-43.

2. Belizan M, Bergh AM, Cilliers C, Pattinson RC, Voce A, Synergy G. Stages of change: A qualitative study on the implementation of a perinatal audit programme in South Africa. BMC Health Serv Res. 2011;11:243.

3. Bhanbhro S, Gee M, Cook S, Marston L, Lean M, Killaspy H. Recovery-based staff training intervention within mental health rehabilitation units: a two-stage analysis using realistic evaluation principles and framework approach. BMC Psychiatry. 2016;16:292.

4. Bouamrane MM, Mair FS. Implementation of an integrated preoperative care pathway and regional electronic clinical portal for preoperative assessment. BMC Med Inform Decis Mak. 2014;14:93.

5. Bridges J, May C, Fuller A, Griffiths P, Wigley W, Gould L, et al. Optimising impact and sustainability: a qualitative process evaluation of a complex intervention targeted at compassionate care. BMJ Qual Saf. 2017;26(12):970-7.

6. Campbell S, Pieters K, Mullen KA, Reece R, Reid RD. Examining sustainability in a hospital setting: case of smoking cessation. Implement Sci. 2011;6:108.

7. Fleiszer AR, Semenic SE, Ritchie JA, Richer MC, Denis JL. A unit-level perspective on the long-term sustainability of a nursing best practice guidelines program: An embedded multiple case study. Int J Nurs Stud. 2016;53:204-18.

8. Fleiszer AR, Semenic SE, Ritchie JA, Richer MC, Denis JL. An organizational perspective on the long-term sustainability of a nursing best practice guidelines program: a case study. BMC Health Serv Res. 2015;15:535.

9. Frykman M, von Thiele Schwarz U, Muntlin Athlin A, Hasson H, Mazzocato P. The work is never ending: uncovering teamwork sustainability using realistic evaluation. J Health Organ Manag. 2017;31(1):64-81.

10. Gould DJ, Hale R, Waters E, Allen D. Promoting health workers' ownership of infection prevention and control: using Normalization Process Theory as an interpretive framework. J Hosp Infect. 2016;94(4):373-80.

11. Gramlich LM, Sheppard CE, Wasylak T, Gilmour LE, Ljungqvist O, Basualdo-Hammond C, et al. Implementation of Enhanced Recovery After Surgery: a strategy to transform surgical care across a health system. Implement Sci. 2017;12(1):67.

12. Green SA, Bell D, Mays N. Identification of factors that support successful implementation of care bundles in the acute medical setting: a qualitative study. BMC Health Serv Res. 2017;17(1):120.

13. Hommel A, Gunningberg L, Idvall E, Baath C. Successful factors to prevent pressure ulcers - an interview study. J Clin Nurs. 2017;26(1-2):182-9.

14. Hovlid EB, O.;Haug, K.;Aslaksen, A. B.;von Plessen, C. Sustainability of healthcare improvement: what can we learn from learning theory? BMC health services research. 2012;12:235.

15. Ilott I, Gerrish K, Eltringham SA, Taylor C, Pownall S. Exploring factors that influence the spread and sustainability of a dysphagia innovation: an instrumental case study. BMC Health Serv Res. 2016;16(1):406.

16. Matthew-Maich N, Ploeg J, Dobbins M, Jack S. Supporting the Uptake of Nursing Guidelines: what you really need to know to move nursing guidelines into practice. Worldviews Evid Based Nurs. 2013;10(2):104-15.

17. McClung L. Health care worker perspectives of their motivation to reduce hospital-acquired infections. Journal of Investigative Medicine. 2017;65(4):824.

18. Mitchell SE, Weigel GM, Laurens V, Martin J, Jack BW. Implementation and adaptation of the Re-Engineered Discharge (RED) in five California hospitals: a qualitative research study. BMC Health Serv Res. 2017;17(1):291.

19. Naldemirci O, Wolf A, Elam M, Lydahl D, Moore L, Britten N. Deliberate and emergent strategies for implementing person-centred care: a qualitative interview study with researchers, professionals and patients. BMC Health Serv Res. 2017;17(1):527.

20. Nordmark S, Zingmark K, Lindberg I. Process evaluation of discharge planning implementation in healthcare using normalization process theory. BMC Med Inform Decis Mak. 2016;16:48.

21. Parand A, Benn J, Burnett S, Pinto A, Vincent C. Strategies for sustaining a quality improvement collaborative and its patient safety gains. Int J Qual Health Care. 2012;24(4):380-90.

22. Rotteau L, Webster F, Salkeld E, Hellings C, Guttmann A, Vermeulen MJ, et al. Ontario's emergency department process improvement program: the experience of implementation. Acad Emerg Med. 2015;22(6):720-9.

23. Sanchez SH, Sethi SS, Santos SL, Boockvar K. Implementing medication reconciliation from the planner's perspective: a qualitative study. BMC Health Serv Res. 2014;14:290.
