# Supplementary material for: The barriers and facilitators influencing the sustainability of hospital-based interventions: a systematic review
Source: BMC Health Serv Res. 2020 Jun 28;20:588. doi: 10.1186/s12913-020-05434-9 (PMC7321537; doi:10.1186/s12913-020-05434-9)
Supplement: Supplementary file 6 — Additional file 6. Methodological quality assessment for mixed method design studies. [file 12913_2020_5434_MOESM6_ESM.docx]

| **First author (Year)** | **MMAT: Clear aim?** | **MMAT Appropriate data collection** | **MMAT Qual Relevant data sources** | **MMAT Qual Relevant data analysis** | **MMAT Qual Context relevant findings** | **MMAT Qual Researcher's influence** | **MMAT Quant 3. Recruitment to minimise selection bias** | **MMAT Quant 3. Appropate measurements** | **MMAT Quant 3. Difference between groups** | **MMAT 3. Complete outcome data/response rate** | **MMAT Quant 4. Relevant sampling strategy** | **MMAT Quant 4. Representative sample?** | **MMAT Quant 4. Appropriate measures** | **MMAT 4. Acceptable response rate** | **MMAT Mixed 5. Relevant design** | **MMAT Mixed 5. Relevant integration** | **MMAT Mixed 5. Limitations of integration** |
| --- | --- | --- | --- | --- | --- | --- | --- | --- | --- | --- | --- | --- | --- | --- | --- | --- | --- |
| Bergh (2014) | "to provide a systematic 'snapshot' of the implementation status of facility-based KMC services in four countries in sub-Saharan Africa to inform further roll-out of KMC and other health system interventions." | Stated as cross-sectional mixed method design. MMAT: Qualitative treated as 1.E. Case study Quantitative treated as 3.D. Cross-sectional analytic study Mixed method treated as C. Triangulation design | Sampling was from countries expected to yield rich data on basis of perceived progress and existing platforms for delivering newborn care. Relevant sources, but limited details provided: national plenary events (or in one country interviews) to provide "the back-drop"; range of numbers given. Also interviews at site visits, but no detail of numbers. | Relevant, but VERY limited on detail: "…the themes derived from the open-ended items on the questionnaires were then compared with the themes recorded in the stakeholder meetings and interviews" | As a multi-country assessment, the relevance of context is considered throughout, while looking for common barriers and facilitators. (See eg Discussion para 1) | Not discussed | The authors call it a convenience sample (and acknowledge in limitations as meaning is "not generalizable to the remainder of the facilities in the countries surveyed") but it was also a purposive sample to get the richest data. | Interview questionnaire and observation inventory (previously applied in and adapted for other countries) used to generate descriptive statistics and an implementation progress score. | See section "Country progress with the implementation of facility-based KMC" and additional file 2 'Summary of key findings by country' | Assessor-completed on site visits, so assume 100%. | **NA** | **NA** | **NA** | **NA** | This is an evaluation of the stage a facility has reached; it also identifies barriers and facilitators. Authors clearly account for context by integrating stakeholder meeting analysis with site analysis. | Covered in Methods section across Sampling, Data collection and Data analysis | Not addressed specifically by authors but implicit in "The final interpretation was confirmed by key role players in each country." |
| Bernstein (2009) | "This article describes and evaluates this dissemination of SBIRT to seven EDS across the state." | Stated as mixed method.  MMAT: Qualitative treated as 1.F. Qualitative description Quantitative treated as 4.A. Incidence or prevalence study without comparison group Mixed method treated as 5.C. Embedded design | Little detail reported. Interview data collected anonymously (as per ethical conditions) in purposively selected sites by phone (24 conducted out of 30 given the number): "Because the responses were anonymous, we could not identify non-responders to characterize differences." Limited detail about roles of those included appears in the Abstract. | Little detail of analysis process given - see p.1227 METHODOLOGY. Deductive analysis and thematic coding using RE-AIM. | See THE SITE SELECTION PROCESS - criteria for site funding - and METHODOLOGY rationale for interviews at five continuing sites only (other two did not have sustainability). Cyktural Context section under Adoption. | Discussed in LIMITATIONS para 1 in terms of selection bias. Independence of interviewers stated in METHODOLOGY ("given a number to call to speak to a person they did not know who was external to the ED SBIRT project". Also rationale behind RE-AIM selection described. | **NA** | **NA** | **NA** | **NA** | Measure = services provided. Data reported in 'Implementation (Documented Service Outcomes). | Data are provided on the number attending ED and the number of screens, and limitations on this. Attempt to show representativeness: "Patients receiving an intervention reflected the demographic distribution of the ED census at the seven sites" | Basic descriptive measures. | Depends on criteria applied, but well reported. Negative screens recorded by count; process for positive screens (e.g. built-in accuracy checks) outlines on p.1227. | Quantative element used to measure implementation (services provided). Qualitative element used "to document reach and evaluate the success of SBI adoption and maintenance." Data "limited to process markers." | NA  (Integration isn't really relevant, as the two parts are performing different but complementary functions.) | NA  Integration isn't really relevant, as the two parts are performing different but complementary functions.) |
| Glasgow (2013) | "As a first step to understanding the necessary organizational components for successful and sustained QI, the authors examine how a collection of survey measures of hospital characteristics related to QI success during a recent Veterans Health Affairs QI collaborative." | MMAT treated as: Quantitative 3.D Cross-sectional analytic study "Given the breadth of available and potentially relevant survey measures, particularly in relation to the number of participating hospitals, they use a data-mining decision tree to analyze these data in a manner that is not constrained by power limitations associated with frequentist statistical analysis." | **NA** | **NA** | **NA** | **NA** | Recruitment was aimed at all 130 VHA hospitals participating in a quality improvement Collaborative. Survey 1 had a 100% response from all VHA acute hospitals (n=130); survey 2 had an 86% response from all VHA hospitals (n=160). | Collaborative (n=130 VHA hospitals): Flow Improvement Inpatient Initiative. Two specific improvement measures evaluated: reduced hospital length of stay, and increased percentage of patients discharged before noon. Four categories: sustain / improve / no trend / no benefit.  Survey information mapped to analytic framework. Two variables added: annual patient volume; percentage of rural patients. | Data-mining is new to me but the modelling process (including decision trees) is reported in detail in the 'Data Evaluation' section, and the 'Results' are presented cautiously in line with its status as a "preliminary study". | "Complete data from the surveys were available for 100 of the 130 facilities that participated in FIX [the Collaborative]…Tests comparing the subset of hospitals with complete data with the full data set did not identify any sources of systematic nonresponse based on hospital characteristics such as facility size or geographic location." | **NA** | **NA** | **NA** | **NA** | **NA** | **NA** | **NA** |
| Jangland (2017) | "to conduct an evaluation of an implementation project on patient participation using two specific research questions" | "The study is a descriptive design using quantitative and qualitative methods." MMAT: Qualitative treated as 1.F. Qualitative description Quantitative treated as 3.D. Cross-sectional analytic study Mixed methods treated as 5.D. Embedded design | Nurse managers (n=5, one from each participating unit.) Summary details of gender, age, time in position, academic qualifications and experience. None refused. | Figure 2 - details of interviews. Table 2 - process of analysis. 'Data analysis' and 'Rigour' sections give brief information. | Methods' includes a section on 'Setting'. Section in 'Results' on 'Trying to move forward in a busy context'; also addressed in 'Discussion' | States that external facilitator of implementation project was first author and that the other author invited nurse managers to interview and carried them out. | Participants' sections: consecutive sample of admitted patients >18 years >1 day stay. Target sample = 200 | Questionnaire 'Quality from the Patient's Perspective' is relevant to the research question. States has been tested for validity and reliability (referenced). Further information in Table 3. | Table 3 gives the background information of participants. Stated rather than reflected on. | Response rate "approximately 62%" (but see limitations - assumed based on previous study not this one). Table 4 = Patients' (n=198) perception of the quality of care, with number answering each dimension/factor other than 'not applicable' also given. | **NA** | **NA** | **NA** | **NA** | The quantitative element examined patients' perception of the quality of care, and the qualitative the nurse managers' views on the care intervention. This is used to discuss why the intervention may not have had the desired impact on patient care. | During interpretation, as in 'Discussion' | Integration of data isn't relevant, as the two parts are performing completely different but complementary functions so integration is in the interpretation. Limation listed = "the number of internal dropouts" |
| Mazzocato (2012) | "we aimed to unpack how and why such a lean application may work" | Described as a "mixed methods explanatory single case study" MMAT treated as: Qualitative: 1.E. Case study Quantitative: unsure - 4.A. Incidence or prevalence study without comparison group Mixed methods: 5.D. Embedded design | Focus of non-participant observation described. Focus of semi-structured interviews with key people described. Documents collected. Period of data collection stated. Limited detail of interviewees (job role) and participation. | 40 hours of non-participant observation, meetings, 13 semi-structured interviews with key people, documents. Focus of each described, including before/after intervention and flow. Intervention phase retrospective, implementation phase prospective. 'Data analysis' section: case description in 3 steps, case analysis process summarised, steps to strengthen quality. | As a single case study, detail about the context is included, and limitations on generalisability acknowledged. | Not discussed | **NA** | **NA** | **NA** | **NA** | "Performance and patient volume data were collected for 52 weeks before and 104 weeks after the implementation of lean-inspired changes" - proportion of patients leaving A&E within 4 hours and waiting time from triage to first A&E physician consultation collected as hospital's weekly averages. | Includes all patients going through A&E | Detail given in 'Data analysis' about ANOVA complemented by statistical process control charts, with planning built in to detect special-cause variation (i.e. a possible effect of the intervention rather than natural variation). | As was a measure of all patients going through A&E and no mention is made of a problem, have to assume that the data is largely complete | In-depth case study showed how results (quantitative) were achieved and sustained (qualitative) | Integration is relevant to the interpretation of the case, as the data from the two parts are performing completely different but complementary functions. | See 'Methodological considerations and future research' |
| Robert (2011) | "to explore the local adoption, implementation and assimilation of one such innovation into routine nursing practice by applying an evidence-based diffusion of innovations framework to a national quality improvement programme." | "We adopted a mixed-method approach, guided by the diffusion of innovation framework". MMAT treated as: Qualitative ('Organisational case studies'): 1.E. Case study Quantitative: 3.D. Cross-sectional analytic study Mixed method: 5.C. Triangulation design | Purposive selection of sites (Table 2) and interviewees described, no. of interviews (58). Interviewees nominated by leads "to gain a pciture of implementation 'ward to board'." No further detail given, other than that the research "combined first-hand accounts, documentary analysis and observation of local implementation." | Interviews recorded and transcribed for analysis. Little detail given about process, other than issues in interview schedule but 'Methods' section gives an overall account of the approach and "narrative strategy" and refers readers to the protocol. Important limitation to analysis included: no inclusion of those who did not implement the intervention. State that cross-case summary tables were used to synthesise key findings from primary data. | Built in to design. | "our stance" outlined in 'Methods'. | National online survey targeted acute hospitals implementing or considering the intervention. No information given about how it was circulated to reach the intended staff with experience of organisational-level implementation, but 150 responses received. Brief information about the breakdown of role in relation to the intervention suggests 72% were very closely involved. | Open and closed questions used: around framework, local implementation and availability / use of local data. Tables 3 and 4 give detailed breakdown of responses to a number of questions (strength of agreement) around engagement and support, and organisation's characteristics respectively. | Built in to study design. | NA  (Purpose was "to collect data sufficiently broad to address the aim of the study" which 150 relevant responses would do) | **NA** | **NA** | **NA** | **NA** | Yes, both are used "to collect data sufficiently broad to address the aim of the study" and "construct 'stories' from the various strands of data." | "For the purposes of this paper, we have focused on synthesis of the survey and case study data - to draw out lessons for policy, practice and research." Readers referred to full report for further detail of process. Also collected routine data to provide complementary information mapping the adoption of the intervention nationally. Reporting refers to what survey respondents / interviewees / both said. | 3 main limitations specified: measuring adoption; lack of inclusion of non-adopters; lack of comparable quantitative measures of impact. |
| Stacey (2015) | "to evaluate a sustainable approach for implementing the lung transplant referral patient decision aid into clinical practice in adult CF clinics" - 2 specific objectives also stated. | Two types of survey: patient decision aid use (survey repeated after 2 years, complemented by tracking logs); barriers assessment (baseline and re-administered 2 years later). Surveys sent to all 23 accredited adult CF clinics in Canada.  MMAT treated as: Quantitative: 3.B. Cohort study | **NA** | **NA** | **NA** | **NA** | All 23 clinics invited to participate: 18 agreed, 3 agreed to provide information on use only (none gave a rationale and subsequently reported intervention not used), and 2 declined because routinely refer on without taking patient preference into account. Of the 31 responses to the healthcare professionals barrier survey, at least one came from each participating clinic. | Survey items "previously validated in a study of physicians using principal component analysis and subsequently validated in studies of nurses and other non-physician healthcare professionals". Identified barriers used to design interventions. | As much information is given as authors have access to, e.g. 15 of the 18 clinics who agreed to participate actually took part. "Despite improved use of the patient decision aids with 15 participating CF clinics, the other 8 CF clinics in Canada that did not fully participate in the study had no or unclear use of the patient decision aid. Therefore, the tailored interventions were not adequate for all eligible CF clinics and qualitative research could be helpful for exploring reasons for non-participation and/or non-use". | 15 of the 18 clinics who agreed to participate actually did so. 78% of CF clinics involved fully, 13% partially. Detail given on 'Sustained use of the patient decision aid'. 28/31 healthcare professionals completed the barriers survey at baseline and end (3 baseline only). | **NA** | **NA** | **NA** | **NA** | **NA** | **NA** | **NA** |

**ADDITIONAL FILE 6. METHODOLOGICAL QUALITY ASSESSMENT FOR MIXED METHOD STUDIES**

Colour code key: Green = yes; Orange = can’t tell; Red = no.

Abbreviations: NA: not applicable
