# Supplementary material for: The barriers and facilitators influencing the sustainability of hospital-based interventions: a systematic review
Source: BMC Health Serv Res. 2020 Jun 28;20:588. doi: 10.1186/s12913-020-05434-9 (PMC7321537; doi:10.1186/s12913-020-05434-9)
Supplement: Supplementary file 7 — Additional file 7. Methodological quality assessment for quality improvement studies. [file 12913_2020_5434_MOESM7_ESM.docx]

**ADDITIONAL FILE 7. METHODOLOGICAL QUALITY ASSESSMENT FOR QUALITY IMPROVEMENT STUDIES**

Colour code key: Green = yes; Orange = can’t tell; Red = no.

| **First author (Year)** | **SQUIRE 1: Title?** | **SQUIRE 2: Abstract?** | **SQUIRE 3: Introduction, problem description?** | **SQUIRE 4: Available knowledge?** | **SQUIRE 5: Rationale?** | **SQUIRE 6: Specific aims?** | **SQUIRE 7: Methods context?** | **SQUIRE 8: Interventions?** | **SQUIRE 9: Study of the interventions?** | **SQUIRE 10: Measures?** | **SQUIRE 11: Analysis** | **SQUIRE 12: Ethical consideration?** | **SQUIRE 13: Results** | **SQUIRE 14: Discussion summary?** | **SQUIRE 15: Interpretation?** | **SQUIRES 16: Limitations?** | **SQUIRES 17: Conclusions** | **SQUIRES 18: Other information (funding)** |
| --- | --- | --- | --- | --- | --- | --- | --- | --- | --- | --- | --- | --- | --- | --- | --- | --- | --- | --- |
| Brady (2014) (1) | Title includes 'a rapid cycle improvement project' | Structured and detailed | Succinct | Contrasted traditional treatment with recent studies | Under 'Planning the intervention' (seven key drivers) | Paragraph on purpose in introduction; specific aim under 'Planning the intervention' | Setting described in detail | Under 'Planning the intervention' - team and four improvement activities described; these activities each have detailed sub-sections | Clear criteria / rationale for identifying eligible subjects; process for obtaining / recording baseline and post- intervention patient data described | Section 'Outcome assessment' - survey and observational time series study processes and balancing measure described. | Analysis' section - all quantitative. | Human subject’s protection' section; also reference to 'ethical imperative' on p.506 | Detailed | Para 1 of 'Discussion' | Clearly address all elements | Final paragraph | Some of these addressed in Discussion rather than Conclusions section | Full detail on Contributors, one author supported by a funding award |
| White (2011)  (2) | "Utilising improvement science methods to optimise…" | Structured and detailed | Succinct | Brief | Brief | Final paragraph of introduction | Subsection 'Setting'. | Subsection 'Planning the intervention' with more detail in 'Improvement activities' | Section 'Improvement activities', figure 1 flow diagram and figure 2 driver diagram. | Small section 'Measures' gives primary outcome measure. 'Data collection' describes process | Section 'Analysis' includes detail on variation | Section 'Human subject protection' | Section 'results' | Opening paragraph of 'Discussion'. | Remainder of 'Discussion' | Lengthy 'Limitations' section | Brief | Competing interests listed as 'none' but no specific mention of funding |

**References**

1. Brady PW, Brinkman WB, Simmons JM, Yau C, White CM, Kirkendall ES, et al. Oral antibiotics at discharge for children with acute osteomyelitis: a rapid cycle improvement project. BMJ Qual Saf. 2014;23(6):499-507.

2. White CM, Schoettker PJ, Conway PH, Geiser M, Olivea J, Pruett R, et al. Utilising improvement science methods to optimise medication reconciliation. BMJ Qual Saf. 2011;20(4):372-80.
