# Supplementary material for: The barriers and facilitators influencing the sustainability of hospital-based interventions: a systematic review
Source: BMC Health Serv Res. 2020 Jun 28;20:588. doi: 10.1186/s12913-020-05434-9 (PMC7321537; doi:10.1186/s12913-020-05434-9)
Supplement: Supplementary file 8 — Additional file 8. Table of included studies mapped to Moore (2017) definition of sustainability. [file 12913_2020_5434_MOESM8_ESM.docx]

**ADDITIONAL FILE 8. TABLE OF INCLUDED STUDIES MAPPED TO MOORE (2017) DEFINITION OF SUSTAINABILITY BY TWO INDEPENDENT REVIEWERS.**

Moore *et al^1^* created a revised definition of sustainability comprising the following five key constructs: (a) After a defined period of time; (b) The programme, clinical intervention and/or implementation strategies *continue to be delivered; (c)* Individual behaviour change is maintained. (d) The programme and individual behaviour change may evolve or adapt and (e) Continuing to produce benefits for individuals/systems.

**Abbreviations:** BCI – behavioural change intervention, N – no, NR – not reported, P – partially reported, Y – yes

| **Author (year)** | **(A) Defined period of time?**  **(months)** | **(B) Continues to be delivered?** | **(C) Behaviour change maintained?** | **(D) Evolved or adapted?** | **(E ) Longer term benefits?** |
| --- | --- | --- | --- | --- | --- |
| 1. Ament  2. 2017 ^2^ | Y  (36 – 72) | Y  (Informal lunches / 3 months) | P  (Sustainability varied between hospitals) | Y  (Adaptability reported as a factor for sustainability) | P  (Early implementation success) |
| 1. Belizan  2. 2011 ^3^ | Y  (36 – 60) | Y  (Review meetings, regional/provincial coordinator to ensure sustainability) | Y  (All sites reached the institutionalision phase where the audit becomes part of the routine) | NR | NR |
| 1. Bergh ^4^  2. 2014 | P  (Implementation started Malawi 1999; Uganda 2002; Rwanda 2007; Mali 2008. Unclear when study was actually conducted) | P  (Length of delivery and consistency is unclear) | P  (Maintained in 2/4 countries) | NR | P  (Govt. Policy documents amended) |
| 1. Bernstein  2. 2009 ^5^ | Y  (18) | Y  (Throughout - Monthly teleconferences and quarterly educational workshops to review / share successes /challenges) | Y  (Only interviewed at sites that maintained change) | NR | Y  (At the outset training conducted to make program self-sufficientmaintain and easy to. Investment in marketing to key stakeholders) |
| 1. Bhanbhro  2. 2016 ^6^ | Y  (12) | Y  (Regular meetings) | P  (Legacy described as mixed; staff reverted to previous demarcations and behaviours. Reasoning: insufficient prep; brevity of engagement of intervention team; misunderstanding aims of intervention; resources (lack of); role boundaries; leadership; competing priorities) | NR | P  (Mixed evidence across units) |
| 1. Bouamrane and Mair  2. 2014 ^7^ | Y  (24) | Y  (Embedded in existing systems) | Y  (Form was embedded) | Y  (Ability to adapt the system and keep it up to date) | Y  (Consensual and contextualised implementation key to embedding into routine use) |
| 1. Brady  2. 2014 ^8^ | Y  (>12) | NR | NR | NR | NR |
| 1. Bridges  2. 2017 ^9^ | Y  (12) | P  (Evidenced via interviews) | P  (All staff were able to articulate ongoing activitives associated with the intervention. However ownership for maintaining the intervention variable) | Y  (Describes evidence of PDN working with staff to make intervention more flexible and fit better with resource pressures) | NR |
| 1. Campbell  2. 2011 ^10^ | NR  (Implemented in 2002 in 1 hospital; more hospitals in 2006 and 2008. Estimate between 2 – 9 years, although not explicitly reported) | NR | Y  (Only included units that achieved sustainability) | NR | NR  (Not explicitly reported but hospitals describe improvement over time) |
| 1. Fleiszer  2. 2015 ^11^ | Y  (96) | Y  (Continuously revisiting) | Y  (Results sustained over 8 year period (though they would have liked to promote further improvement) | Y  (Evolution had occurred: adaption of original program and implementation of a subsequent related innovation) | Y  (Hospitals were selected where benefits were maintained) |
| 1. Fleiszer  2. 2016 ^12^ | Y  (84-96) | P  (Educational sessions; unit based champion support; audit and feedback) | P  (For some units became routized and sustained. Practice change, sustained-orieted acivities, favorable influencing factors) | P  (Development, specifically in terms of adaption of the unit context had occurred. This was seen to contribute to program sustainability) | P  (Persistent benefits over the long term seen in some cases) |
| 1. Frykman  2. 2017 ^13^ | Y  (24) | P  (Reduced frequency and adaptation for a few strategies: low level of sustainability was concomitant with only marginal changes in context but a substantial fallback in BCI) | P  (Decreased evidence of interventions, but not reported that it has disappeared. However, would say only minimal behaviour change reported) | NR | NR |
| 1. Glasgow  2. 2013 ^14^ | Y  (60) | P  (Audit and time series analysis done on the data) | NR | NR | NR |
| 1. Gould  2. 2016 ^15^ | Y  (18) | P  (Charts for feeding back information, audits) | NR  (Audit shows infection rates reduced) | NR | NR |
| 1. Gramlich  2. 2017 ^16^ | Y  (32) | Y  (Audit) | Y  (Increase in % adhering to best practice) | NR | Y  (Work begun on overcoming barriers identified in this study e.g. rural patients) |
| 1. Green  2. 2017 ^17^ | Y  (6-18) | Y  (6 and 18 month progress review) | NR | Y  (Iterative development of the initiatives with aims reviewed and adapted) | NR |
| 1. Hommel  2. 2017 ^18^ | Y  (36) | Y  (Some used audit/regular collection of data) | Y  (Feedback via audit/meetings suggested so) | NR | Y  (Part of the inclusion criteria) |
| 1. Hovlid  2. 2012 ^19^ | Y  (24) | NR | NR | Y  (Frontline employees engaged in adaptions and modifications of the interventions) | Y  (The improvement strategy  triggered clinicians to inquire about their system and opened an arena for information sharing and for relating these activities  to the context of the whole clinical system) |
| 1. Ilott  2. 2016 ^20^ | Y  (34) | P  (Mealtime observations, knowledge and attitude survey, interviews) | P  (Implied by responses and issues raised in data collected [JC]) | NR | P  (Policy changes and directive introduced) |
| 1. Jangland and Gunningberg  2. 2017 ^21^ | Y  (24) | Y  (External facilitators) | No  (Change was not embedded) | NR | N  (Change was not embedded) |
| 1. Matthew-Maich  2. 2013 ^22^ | Y  (6-24) | Y  (Interviews, field notes, questionnaires) | P  (Sustained at 2 sites) | Y  (Tailored strategies used by frontline staff to foster change) | Y  (Recognition of need to engage in evidence-based practice) |
| 1. Mazzocato  2. 2012 ^23^ | Y  (24) | NR | NR | NR | Y  (Clear definition of roles and their interdependencies) |
| 1. McClung  2. 2017 ^24^ | Y  (10 -48) | Y  (Feedback and monitoring) | NR  (Degree of sustainability is not reported but report that bundles have been in place for several months to years) | NR | NR |
| 1. Mitchell  2. 2017 ^25^ | P  (>6) | P  (‘Yes for sites with sustained change, but not for all patients’) | P  (Might be due to other initiatives) | Y  (Reference to adaption the original protocol whilst maintaining a high level of fidelity) | Y  (Staff feel experts / consultants in the area addressed by the interventions. Sustainability needs to be part of the initial vision) |
| 1.Naldemirci  2. 2017 ^26^ | NR  (Estimate 24 – between dates of related publications) | Y  (Implied from interview analysis that was still delivered. Engaged in continuous education, small group discussions) | P  (Sustainability is implied) | Y  (Discussions had as to how to practice the intervention on their ward) | NR |
| 1. Nordmark  2. 2016 ^27^ | Y  (36) | N  (Workshops, interviews, surveys) | P  (It is not normalised into everyday behaviour but established roles nay help with this) | NR | P  (More defined roles will help normalise the change) |
| 1. Parand  2. 2012 ^28^ | Y  (12) | NR | NR | NR | NR |
| 1. Robert  2. 2011 ^29^ | Y  (15) | Y  (Surveys, interviews though not reported when these were conducted) | P  (40% hospitals adopted the programme) | NR | NR |
| 1. Rotteau  2. 2015 ^30^ | Y  (6-8) | Y  (Reported within the 6-8 months via inteviews | P  (Slippage reported in some cases) | NR | P  (Supported engagement in other QI initiatives) |
| 1. Sanchez  2. 2014 ^31^ | Y  (36) | Y  (Monitoring performed) | NR | Y  (Recognised process of implementation needs to be dynamic and adapted over time) | NR |
| 1. Stacey  2. 2015 ^32^ | Y  (24) | Y  (Results of survey at 24 months) | Y  (Sustained use was measured for 80% eligible patients) | NR | NR |
| 1. White  2. 2011 ^33^ | Y  (27) | Y  (Audit over duration of the study. Annual training) | Y  (Increase in adherence measured at end of 27 months) | NR | NR |

**References**

1. Moore JE, Mascarenhas A, Bain J, Straus SE. Developing a comprehensive definition of sustainability. *Implement Sci* 2017;**12**:110. <http://dx.doi.org/10.1186/s13012-017-0637-1>

2. Ament SMC, Gillissen F, Moser A, Maessen JMC, Dirksen CD, von Meyenfeldt MF*, et al.* Factors associated with sustainability of 2 quality improvement programs after achieving early implementation success. A qualitative case study. *J Eval Clin Pract* 2017;**23**:1135-43. <http://dx.doi.org/10.1111/jep.12735>

3. Belizan M, Bergh AM, Cilliers C, Pattinson RC, Voce A, Synergy G. Stages of change: A qualitative study on the implementation of a perinatal audit programme in South Africa. *BMC Health Serv Res* 2011;**11**:243. <http://dx.doi.org/10.1186/1472-6963-11-243>

4. Bergh AM, Kerber K, Abwao S, de-Graft Johnson J, Aliganyira P, Davy K*, et al.* Implementing facility-based kangaroo mother care services: lessons from a multi-country study in Africa. *BMC Health Serv Res* 2014;**14**:293. <http://dx.doi.org/10.1186/1472-6963-14-293>

5. Bernstein E, Topp D, Shaw E, Girard C, Pressman K, Woolcock E*, et al.* A preliminary report of knowledge translation: lessons from taking screening and brief intervention techniques from the research setting into regional systems of care. *Acad Emerg Med* 2009;**16**:1225-33. <http://dx.doi.org/10.1111/j.1553-2712.2009.00516.x>

6. Bhanbhro S, Gee M, Cook S, Marston L, Lean M, Killaspy H. Recovery-based staff training intervention within mental health rehabilitation units: a two-stage analysis using realistic evaluation principles and framework approach. *BMC Psychiatry* 2016;**16**:292. <http://dx.doi.org/10.1186/s12888-016-0999-y>

7. Bouamrane MM, Mair FS. Implementation of an integrated preoperative care pathway and regional electronic clinical portal for preoperative assessment. *BMC Med Inform Decis Mak* 2014;**14**:93. <http://dx.doi.org/10.1186/1472-6947-14-93>

8. Brady PW, Brinkman WB, Simmons JM, Yau C, White CM, Kirkendall ES*, et al.* Oral antibiotics at discharge for children with acute osteomyelitis: a rapid cycle improvement project. *BMJ Qual Saf* 2014;**23**:499-507. <http://dx.doi.org/10.1136/bmjqs-2013-002179>

9. Bridges J, May C, Fuller A, Griffiths P, Wigley W, Gould L*, et al.* Optimising impact and sustainability: a qualitative process evaluation of a complex intervention targeted at compassionate care. *BMJ Qual Saf* 2017;**26**:970-7. <http://dx.doi.org/10.1136/bmjqs-2017-006702>

10. Campbell S, Pieters K, Mullen KA, Reece R, Reid RD. Examining sustainability in a hospital setting: case of smoking cessation. *Implement Sci* 2011;**6**:108. <http://dx.doi.org/10.1186/1748-5908-6-108>

11. Fleiszer AR, Semenic SE, Ritchie JA, Richer MC, Denis JL. An organizational perspective on the long-term sustainability of a nursing best practice guidelines program: a case study. *BMC Health Serv Res* 2015;**15**:535. <http://dx.doi.org/10.1186/s12913-015-1192-6>

12. Fleiszer AR, Semenic SE, Ritchie JA, Richer MC, Denis JL. A unit-level perspective on the long-term sustainability of a nursing best practice guidelines program: An embedded multiple case study. *Int J Nurs Stud* 2016;**53**:204-18. <http://dx.doi.org/10.1016/j.ijnurstu.2015.09.004>

13. Frykman M, von Thiele Schwarz U, Muntlin Athlin A, Hasson H, Mazzocato P. The work is never ending: uncovering teamwork sustainability using realistic evaluation. *J Health Organ Manag* 2017;**31**:64-81. <http://dx.doi.org/10.1108/JHOM-01-2016-0020>

14. Glasgow JM, Yano EM, Kaboli PJ. Impacts of organizational context on quality improvement. *Am J Med Qual* 2013;**28**:196-205. <http://dx.doi.org/10.1177/1062860612456730>

15. Gould DJ, Hale R, Waters E, Allen D. Promoting health workers' ownership of infection prevention and control: using Normalization Process Theory as an interpretive framework. *J Hosp Infect* 2016;**94**:373-80. <http://dx.doi.org/10.1016/j.jhin.2016.09.015>

16. Gramlich LM, Sheppard CE, Wasylak T, Gilmour LE, Ljungqvist O, Basualdo-Hammond C*, et al.* Implementation of Enhanced Recovery After Surgery: a strategy to transform surgical care across a health system. *Implement Sci* 2017;**12**:67. <http://dx.doi.org/10.1186/s13012-017-0597-5>

17. Green SA, Bell D, Mays N. Identification of factors that support successful implementation of care bundles in the acute medical setting: a qualitative study. *BMC Health Serv Res* 2017;**17**:120. <http://dx.doi.org/10.1186/s12913-017-2070-1>

18. Hommel A, Gunningberg L, Idvall E, Baath C. Successful factors to prevent pressure ulcers - an interview study. *J Clin Nurs* 2017;**26**:182-9. <http://dx.doi.org/10.1111/jocn.13465>

19. Hovlid EB, O.;Haug, K.;Aslaksen, A. B.;von Plessen, C. Sustainability of healthcare improvement: what can we learn from learning theory? *BMC health services research* 2012;**12**:235.

20. Ilott I, Gerrish K, Eltringham SA, Taylor C, Pownall S. Exploring factors that influence the spread and sustainability of a dysphagia innovation: an instrumental case study. *BMC Health Serv Res* 2016;**16**:406. <http://dx.doi.org/10.1186/s12913-016-1653-6>

21. Jangland E, Gunningberg L. Improving patient participation in a challenging context: a 2-year evaluation study of an implementation project. *J Nurs Manag* 2017;**25**:266-75. <http://dx.doi.org/10.1111/jonm.12459>

22. Matthew-Maich N, Ploeg J, Dobbins M, Jack S. Supporting the Uptake of Nursing Guidelines: what you really need to know to move nursing guidelines into practice. *Worldviews Evid Based Nurs* 2013;**10**:104-15. <http://dx.doi.org/10.1111/j.1741-6787.2012.00259.x>

23. Mazzocato PH, R. J.;Brommels, M.;Aronsson, H.;Backman, U.;Elg, M.;Thor, J. How does lean work in emergency care? A case study of a lean-inspired intervention at the Astrid Lindgren Children's hospital, Stockholm, Sweden. *BMC health services research* 2012;**12**:28.

24. McClung L. Health care worker perspectives of their motivation to reduce hospital-acquired infections. *Journal of Investigative Medicine* 2017;**65**:824. <http://dx.doi.org/http://dx.doi.org/10.1136/jim-2017-000448.40>

25. Mitchell SE, Weigel GM, Laurens V, Martin J, Jack BW. Implementation and adaptation of the Re-Engineered Discharge (RED) in five California hospitals: a qualitative research study. *BMC Health Serv Res* 2017;**17**:291. <http://dx.doi.org/10.1186/s12913-017-2242-z>

26. Naldemirci O, Wolf A, Elam M, Lydahl D, Moore L, Britten N. Deliberate and emergent strategies for implementing person-centred care: a qualitative interview study with researchers, professionals and patients. *BMC Health Serv Res* 2017;**17**:527. <http://dx.doi.org/10.1186/s12913-017-2470-2>

27. Nordmark S, Zingmark K, Lindberg I. Process evaluation of discharge planning implementation in healthcare using normalization process theory. *BMC Med Inform Decis Mak* 2016;**16**:48. <http://dx.doi.org/10.1186/s12911-016-0285-4>

28. Parand A, Benn J, Burnett S, Pinto A, Vincent C. Strategies for sustaining a quality improvement collaborative and its patient safety gains. *Int J Qual Health Care* 2012;**24**:380-90. <http://dx.doi.org/10.1093/intqhc/mzs030>

29. Robert G, Morrow E, Maben J, Griffiths P, Callard L. The adoption, local implementation and assimilation into routine nursing practice of a national quality improvement programme: the Productive Ward in England. *J Clin Nurs* 2011;**20**:1196-207. <http://dx.doi.org/10.1111/j.1365-2702.2010.03480.x>

30. Rotteau L, Webster F, Salkeld E, Hellings C, Guttmann A, Vermeulen MJ*, et al.* Ontario's emergency department process improvement program: the experience of implementation. *Acad Emerg Med* 2015;**22**:720-9. <http://dx.doi.org/10.1111/acem.12688>

31. Sanchez SH, Sethi SS, Santos SL, Boockvar K. Implementing medication reconciliation from the planner's perspective: a qualitative study. *BMC Health Serv Res* 2014;**14**:290. <http://dx.doi.org/10.1186/1472-6963-14-290>

32. Stacey D, Vandemheen KL, Hennessey R, Gooyers T, Gaudet E, Mallick R*, et al.* Implementation of a cystic fibrosis lung transplant referral patient decision aid in routine clinical practice: an observational study. *Implement Sci* 2015;**10**:17. <http://dx.doi.org/10.1186/s13012-015-0206-4>

33. White CM, Schoettker PJ, Conway PH, Geiser M, Olivea J, Pruett R*, et al.* Utilising improvement science methods to optimise medication reconciliation. *BMJ Qual Saf* 2011;**20**:372-80. <http://dx.doi.org/10.1136/bmjqs.2010.047845>
