# Supplementary material for: The barriers and facilitators influencing the sustainability of hospital-based interventions: a systematic review
Source: BMC Health Serv Res. 2020 Jun 28;20:588. doi: 10.1186/s12913-020-05434-9 (PMC7321537; doi:10.1186/s12913-020-05434-9)
Supplement: Supplementary file 9 — Additional file 9. Key examples of barriers and facillitators within Initiative design and delivery. [file 12913_2020_5434_MOESM9_ESM.docx]

**ADDITIONAL FILE 9. DEFINITION AND KEY EXAMPLES OF BARRIERS AND FACILITATORS IDENTIFIED IN THE INITIATIVE DESIGN AND DELIVERY THEME**

| **THEME: INITIATIVE DESIGN AND DELIVERY** | **CONSTRUCT** | **DEFINITION (AS DESCRIBED BY LENNOX ET AL. 2018)(1)** | **KEY EXAMPLE (BARRIERS)** | **KEY EXAMPLE (FACILITATORS)** |
| --- | --- | --- | --- | --- |
|  | Demonstrating effectiveness | Assessing or measuring project outcomes and impact. | “lower subs case informants reported that, with time, nurses perceived that there were minimal or no benefits for patients or themselves. Nurses began to believe that routine adherence to guidelines required too many "additional" tasks, instead of being "savings" opportunities for patients and themselves” (Fleiszer 2016, p210) (2) | “Some respondents in the ERAS case noted an economic advantage of early discharge. Respondents from the SSP case mentioned that the proven early postimplementation cost-effectiveness of the program was a factor related to sustainability” (Ament, 2017, p1141) (3) |
|  | Evidence base for the initiative | The evidence that the initiative will provide stated benefits and can credibly achieve them through the initiative plan. | “Lack of sound evidence to underpin IPC was raised as a barrier, especially for medical staff” (Gould 2016, p378) (4) | “The strength of the evidence behind the bundle protocols is a strong motivator to comply” (McClung 2017, p1066) (5) |
|  | Expertise | Having adequate expert knowledge and experience to carry out an initiative. | “There was a learning curve for data accuracy, as several of the HPAs had minimal computer skills and none had previously entered data.” (Bernstein, 2009, 1229) (6) | “personnel who were confident in their role were better able to handle others' expectations and had a better understanding of when, what, how and with whom information should be exchanged. They observed that confidence came with work experience” (Nordmark 2016, p7) (7) |
|  | Improvement methods | The use of improvement methods to support initiative success and sustainability. | “[Limited] continuous problem solving“(Frykman 2017, p72) (8) | “Hospitals used this performance feedback to make changes to their processes. When two hospitals noticed a decrease in the number of smokers being identified, one began the process to integrate a late-career nurse to provide support to the program” (Campbell 2011, p7) (9) |
|  | Monitoring progress over time | The ability to monitor the initiative using standardised systems or mechanisms over time. | “RNs thought it was difficult to know the reasons for patients' re-admission due to the absence of information about follow-up in the information system” (Nordmark 2016, p8) (7) | “different types of meetings with different functions were mentioned: monthly perinatal review meetings at hospital level were very important and provided a means of encouraging continuous data collection” (Belizan 2011, p4) (10) |
|  | Project duration | How long the project is expected to last and how long resources are available. | “the availability of external grant funding without a mandatory matching pledge of hospital resources limited the required engagement of hospital leaders in certain hospitals to ensure that RED implementation was an organizational priority with strong leadership commitment. This was best exemplified by the lack of accountability linked to the outcomes of the RED implementation effort, most noticeable when grant funding ended.” (Mitchell 2017, p5) (11) | “It took six years for the department to implement the BPGs across almost all of its nursing units…the protracted time period had offered leaders the opportunity to reflect on their experiences of facilitating change with the first cohorts of units and to use the reflections to improve implementation with the later cohorts. [Examples of program adaptation given: support provided by external facilitators to nursing units became more structured..."mini implementations" were done on units (e.g. psychiatry) where the BPGs were less relevant. "Tailored implementations" were done on units where a common type of patient sub-population (e.g. those suffering from dementia) prevented direct adherence to the standard BPG recommendations]” (Fleiszer 2015, p11) (12) |
|  | Project type | The type and design of the initiative. | NONE | “The two implementation teams had identified these issues and aimed to ensure that steps in the implementation process were kept to a minimum.” (Green 2017, p4) (13) |
|  | The problem | The recognition, concern and acceptance of a problem that supports an initiative to address it. | “One challenge for the hospital medicine service to adopt new evidence for children with acute haematogenous osteomyelitis was the fact that our infectious diseases specialists, who were frequently consulted at baseline, rarely recommended treatment with oral antibiotics.” (Brady 2014, p505) (14) | “Key informants (i.e. SCCs and DMs) viewed smoking cessation as **an important health issue** that fit with the hospitals' corporate objectives of restoring health, or with the hospital's smoke-free property initiative “  (Campbell 2011, p5) (9) |
|  | Training and capacity building | Orienting and training staff to be able to deliver the initiative successfully, as well as putting ongoing educational and skill building support in place for new workers. | “Despite help with specific problems, the two hospitals with lower OMSC activity levels indicated that they did not always feel supported” (Campbell 2011, p7) (9) | “Our education intervention was important in presenting and discussing the evidence with physicians” (Brady, 2014, p506) (14) |

**References**

1. Lennox L, Maher L, Reed J. Navigating the sustainability landscape: a systematic review of sustainability approaches in healthcare. Implement Sci. 2018;13(1):27.

2. Fleiszer AR, Semenic SE, Ritchie JA, Richer MC, Denis JL. A unit-level perspective on the long-term sustainability of a nursing best practice guidelines program: An embedded multiple case study. Int J Nurs Stud. 2016;53:204-18.

3. Ament SMC, Gillissen F, Moser A, Maessen JMC, Dirksen CD, von Meyenfeldt MF, et al. Factors associated with sustainability of 2 quality improvement programs after achieving early implementation success. A qualitative case study. J Eval Clin Pract. 2017;23(6):1135-43.

4. Gould DJ, Hale R, Waters E, Allen D. Promoting health workers' ownership of infection prevention and control: using Normalization Process Theory as an interpretive framework. J Hosp Infect. 2016;94(4):373-80.

5. McClung L. Health care worker perspectives of their motivation to reduce hospital-acquired infections. Journal of Investigative Medicine. 2017;65(4):824.

6. Bernstein E, Topp D, Shaw E, Girard C, Pressman K, Woolcock E, et al. A preliminary report of knowledge translation: lessons from taking screening and brief intervention techniques from the research setting into regional systems of care. Acad Emerg Med. 2009;16(11):1225-33.

7. Nordmark S, Zingmark K, Lindberg I. Process evaluation of discharge planning implementation in healthcare using normalization process theory. BMC Med Inform Decis Mak. 2016;16:48.

8. Frykman M, von Thiele Schwarz U, Muntlin Athlin A, Hasson H, Mazzocato P. The work is never ending: uncovering teamwork sustainability using realistic evaluation. J Health Organ Manag. 2017;31(1):64-81.

9. Campbell S, Pieters K, Mullen KA, Reece R, Reid RD. Examining sustainability in a hospital setting: case of smoking cessation. Implement Sci. 2011;6:108.

10. Belizan M, Bergh AM, Cilliers C, Pattinson RC, Voce A, Synergy G. Stages of change: A qualitative study on the implementation of a perinatal audit programme in South Africa. BMC Health Serv Res. 2011;11:243.

11. Mitchell SE, Weigel GM, Laurens V, Martin J, Jack BW. Implementation and adaptation of the Re-Engineered Discharge (RED) in five California hospitals: a qualitative research study. BMC Health Serv Res. 2017;17(1):291.

12. Fleiszer AR, Semenic SE, Ritchie JA, Richer MC, Denis JL. An organizational perspective on the long-term sustainability of a nursing best practice guidelines program: a case study. BMC Health Serv Res. 2015;15:535.

13. Green SA, Bell D, Mays N. Identification of factors that support successful implementation of care bundles in the acute medical setting: a qualitative study. BMC Health Serv Res. 2017;17(1):120.

14. Brady PW, Brinkman WB, Simmons JM, Yau C, White CM, Kirkendall ES, et al. Oral antibiotics at discharge for children with acute osteomyelitis: a rapid cycle improvement project. BMJ Qual Saf. 2014;23(6):499-507.
