# Supplementary material for: The barriers and facilitators influencing the sustainability of hospital-based interventions: a systematic review
Source: BMC Health Serv Res. 2020 Jun 28;20:588. doi: 10.1186/s12913-020-05434-9 (PMC7321537; doi:10.1186/s12913-020-05434-9)
Supplement: Supplementary file 10 — Additional file 10. Key examples of barriers and facilitators identified within the Negotiating initiative processes theme. [file 12913_2020_5434_MOESM10_ESM.docx]

**ADDITIONAL FILE 10. DEFINITION AND KEY EXAMPLES OF BARRIERS AND FACILITATORS IDENTIFIED IN THE NEGOTIATING INITIATIVE PROCESSES THEME.**

| **Theme: Negotiating initiative processes** | **CONSTRUCT** | **DEFINITION (AS DESCRIBED BY LENNOX ET AL. 2018)(1)** | **KEY EXAMPLE (BARRIERS)** | **KEY EXAMPLES (FACILITATORS)** |
| --- | --- | --- | --- | --- |
|  | **Accountability of roles and responsibilities** | Roles and responsibilities involved in the initiative are clearly defined and outlines with necessary distribution across teams as necessary so there is no reliance on specific individuals. | Not all ideas were implemented in practice, which appeared to be linked with uncertainty about whose role it was to realise them or to authorise them (Bridges 2017, p974)(2) | To assure a firm commitment to uptake, hospital and ED administrators at selected sites signed a memorandum of understanding detailing responsibilities and deliverable services (Bernstein, 2009, 1227)(3) |
|  | **Belief in the initiative** | The belief that, the initiative will be of value, it will produce the benefits intended and deliver the stated improvements to care. | Most nurses were initially resistant to try the innovations; they did not trust the practice change(s). They were angry that their workload would be even more demanding… Nurses expressed doubts and fears about the effectiveness and safety of the practice changes...It was challenging to change long-standing practices...They explained that this lasted approximately 4 months, at which time the grumbling stopped and practice changes occurred. This did not occur in the minimal uptake site as no change process was experienced for them. (Matthew-Maich, 2013, p110)(4) | The nurses believed that because the BPG was research-based and came from the nurses' provincial, professional association, this rendered it credible and relevant for their practice. (Matthew-Maich, 2013, p107) (4) |
|  | **Complexity** | The difficulty and complexity of understanding, delivering and maintaining the initiative. | Complex work processes / In both 2010 and 2013, the complexity of the work processes at the ED made it difficult to foresee how changes in one area would affect the other. (Frykman 2017, p73)(5) | The improved team working reduced the burden for some staff and provided opportunities to undertake [patient care] activities that previously would have been rare occurrences. (Bridges 2017, p975)(2) |
|  | **Defining aims and shared vision** | Taking the time to define and understand what people want to achieve and why. Working with stakeholders to establish a shared aim and vision. | In 2013, staff had different views on whether teamwork was the formal way of working, what teamwork encompassed, and how teamwork was connected to the goals at the ED. Direction was weakened as the connection between teamwork and the overall goals of the ED became increasingly unclear to staff. (Frykman 2017, p72)(5) | Findings from both stages emphasised that value was placed on a collaborative goal working approach and service user feedback, as this improved inter-staff relationships, performance and a sense of shared ownership. (Bhanbro 2016, p12)(6) |
|  | **Incentives** | Motivation gained from rewards or benefits that drive individuals and organisations to engage with an initiative and continue to deliver it overtime. | Junior doctors were rarely involved in a structured and sustained way, often with no formal recognition as part of the implementation team. (Green 2017, p4)(7) | Value-added services played a strong role in three hospitals agreeing after 1 year of operation to underwrite part of the HPA salary cost. (Bernstein, 2009, p1231)(3) |
|  | **Job requirements** | Specific job requirements have been established and included in job descriptions and roles which are able to be accomplished with the given skills set of workers. | Team-based work was also viewed by some nurses as "too inflexible" compared to the way work was organised before. [need for more than one nurse in the team] (Mazzocato 2012, p10)(8) | The volume of work now associated with the programme meant that all sites were working to expand towards PW teams with substantive posts and dedicated functions (Robert 2011, p1202)(9) |
|  | **Workload** | The added effort and change to workload when a new initiative in implemented. Staff have the necessary time to complete tasks and the initiative has fair divisions of labour and does not require special or extra effort. | Limited sustainability: Challenges to sustainability were discussed by many of the participants, citing issues such as workload, organizational structures, and lack of accountability. (Rotteau 2015, p726)(10) | The team-based care approach also contributed to the achievement of simple and direct work and patient flows as it connected all care givers involved in a patient's care process. Indeed, some informants argued that the care team approach improved continuity of care as less people were involved in each case. Moreover, as team members were responsible to carry out all the work required to meet the needs of a designated patient, ambiguity was reduced. (Mazzocato 2012, p10)(8) |

**REFERENCES**

1. Lennox L, Maher L, Reed J. Navigating the sustainability landscape: a systematic review of sustainability approaches in healthcare. Implement Sci. 2018;13(1):27.

2. Bridges J, May C, Fuller A, Griffiths P, Wigley W, Gould L, et al. Optimising impact and sustainability: a qualitative process evaluation of a complex intervention targeted at compassionate care. BMJ Qual Saf. 2017;26(12):970-7.

3. Bernstein E, Topp D, Shaw E, Girard C, Pressman K, Woolcock E, et al. A preliminary report of knowledge translation: lessons from taking screening and brief intervention techniques from the research setting into regional systems of care. Acad Emerg Med. 2009;16(11):1225-33.

4. Matthew-Maich N, Ploeg J, Dobbins M, Jack S. Supporting the Uptake of Nursing Guidelines: what you really need to know to move nursing guidelines into practice. Worldviews Evid Based Nurs. 2013;10(2):104-15.

5. Frykman M, von Thiele Schwarz U, Muntlin Athlin A, Hasson H, Mazzocato P. The work is never ending: uncovering teamwork sustainability using realistic evaluation. J Health Organ Manag. 2017;31(1):64-81.

6. Bhanbhro S, Gee M, Cook S, Marston L, Lean M, Killaspy H. Recovery-based staff training intervention within mental health rehabilitation units: a two-stage analysis using realistic evaluation principles and framework approach. BMC Psychiatry. 2016;16:292.

7. Green SA, Bell D, Mays N. Identification of factors that support successful implementation of care bundles in the acute medical setting: a qualitative study. BMC Health Serv Res. 2017;17(1):120.

8. Mazzocato PH, R. J.;Brommels, M.;Aronsson, H.;Backman, U.;Elg, M.;Thor, J. How does lean work in emergency care? A case study of a lean-inspired intervention at the Astrid Lindgren Children's hospital, Stockholm, Sweden. BMC health services research. 2012;12:28.

9. Robert G, Morrow E, Maben J, Griffiths P, Callard L. The adoption, local implementation and assimilation into routine nursing practice of a national quality improvement programme: the Productive Ward in England. J Clin Nurs. 2011;20(7-8):1196-207.

10. Rotteau L, Webster F, Salkeld E, Hellings C, Guttmann A, Vermeulen MJ, et al. Ontario's emergency department process improvement program: the experience of implementation. Acad Emerg Med. 2015;22(6):720-9.
