# Supplementary material for: The barriers and facilitators influencing the sustainability of hospital-based interventions: a systematic review
Source: BMC Health Serv Res. 2020 Jun 28;20:588. doi: 10.1186/s12913-020-05434-9 (PMC7321537; doi:10.1186/s12913-020-05434-9)
Supplement: Supplementary file 11 — Additional file 11. Key examples of barriers and facilitators identified within the people involved theme. [file 12913_2020_5434_MOESM11_ESM.docx]

**SUPPLEMENTARY FILE 11. DEFINITION AND KEY EXAMPLES OF BARRIERS AND FACILITATORS IDENTIFIED IN THE PEOPLE INVOLVED THEME**

| **THEME: PEOPLE INVOLVED** | **CONSTRUCT** | **DEFINITION (AS DESCRIBED BY LENNOX ET AL. 2018)[1]** | **KEY EXAMPLE (BARRIERS)** | **KEY EXAMPLE (FACILITATORS)** |
| --- | --- | --- | --- | --- |
|  | Community Participation | Participation of community members to direct, and shape initiatives to reflect their values, expectations and needs. | “Cultural beliefs (e.g. baby should be carried on the back)” (Bergh et al., 2014, Table 4, p6)[2] | NONE |
|  | Leadership and Champions | A person, or group of people who have the ability and skills to can advocate, communicate and support an initiative to achieve lasting change. | “…the lack of specific, trained and supported change agent/champion posts was associated with poorer long term outcomes in unit 3.” (Bhanbhro et al., 2016, p9-10) [3] | “Monthly meetings with the management group linked the problem solvers to the executive level with the information and authority needed to implement solutions that might otherwise have been beyond the reach of the improvement team, such as additional staffing or changes to work schedules. This helped open lines of communication across hierarchical levels in this large organization.” (Mazzacato et al., 2012 p10) [4] |
|  | Ownership | Organisations, communities and stakeholders taking ownership and responsibility to support, embed and sustain an initiative. | NONE | “Informants continually made reference to their responsibility to ensure that all staff remained aware of the importance of IPC. Senior staff engagement in IPC promoted the collective action of their staff. Clinicians promoted awareness throughout their teams during everyday practice.” (Gould et al, 2016, p377) [5] |
|  | Patient involvement | Involving patients in initiative processes to understand potential impact, values and preferences. | “They thought that patients appreciated the written information on the Tell-us card, but that some patients were not interested or were too tired to write down their own goals for today.” (Jangland et al 2017, 271) [6] | “That said, adapting the RED toolkit site-specifically was also essential in many cases…[one successful hospital] added technology to enhance RED efforts…This hospital was also the only site to add a 'patient advisor' to the implementation team [which] allowed their approach to reflect the needs of the local patient population.” (Mitchell et al., 2017, p8) [7] |
|  | Power | The ability of individuals to use their power to advocate or support the initiative. | “Inter-professional hierarchies (mainly between nurses and doctors)” (Naldermici et al 2017, Table 1, p4) [8] | “staff in our case study sites clearly perceived PW as being different to other quality improvement approaches in terms of giving them a sense of 'permission' to try new ideas and ways of working” (Robert et al., 2011, p1203) [9] |
|  | Relationships and collaboration and networks | Ability to build collaborations, partnerships and networks to support sustainability of the initiative | “because collaborations with other professions and departments had not been preserved or expanded over time, the program had remained largely limited to nursing, despite the necessity for multidisciplinary involvement / Interprofessional collaboration” (Fleizser et al. 2015, Table 2, p7) [10] | “Some hospitals were better at actively seeking support networks than others and had existing informal support networks through which to share their experiences of what works and what does not.” (Robert et al., 2011, p1205) [9] |
|  | Satisfaction | The level of enjoyment and reward stakeholders and staff get from participating in the initiative. | “this approach to improvement also led to some frustration. Due to the large number of employees, including rotating staff, some clinicians (especially those not on the improvement team) felt they could not influence changes and were frustrated about the numerous modifications to the care process. Some also reported uncertainty about how work should be carried out.” (Mazzacato et al., 2012, p10) [4] | “[Nurses] experienced enhanced breastfeeding knowledge, valuing, skill, confidence and autonomy to facilitate breastfeeding success as well as improved professional satisfaction.” (Matthew-Maich et al., 2013, p110) [11] |
|  | Stakeholder participation | The need for involvement and participation from stakeholders who are affected by the initiative | NONE | “The data clearly show that Action Plans in the selected three units were developed collaboratively with all staff members and included management and service users” (Bouamrane and Mair, 2014, p13) [12] |
|  | Staff involvement | Including staff responsible for implementing an initiative across multiple stages of planning, design, delivery and maintenance, valuing their input and taking feedback on board. | “the foundation grant funds were earmarked for nursing care initiatives, but did not set parameters for awardees about how much nursing to use in the implementation of RED. However, in some cases it did promote a strong nursing focus for RED implementation. This focus turned out to be detrimental in Hospital D where the RED implementation was viewed exclusively as a nursing initiative rather than a shared, hospital-wide priority with the multi-disciplinary support necessary to succeed.” (Mitchell et al, 2017, p4-5) [7] | “Team composition: Multiprofessional and equally representing implementation teams were considered important to program success” (Rotteau et al. 2015, Table 4, p724) [13] |

**References**

1. Lennox L, Maher L, Reed J: **Navigating the sustainability landscape: a systematic review of sustainability approaches in healthcare**. *Implement Sci* 2018, **13**(1):27.

2. Bergh AM, Kerber K, Abwao S, de-Graft Johnson J, Aliganyira P, Davy K, Gamache N, Kante M, Ligowe R, Luhanga R *et al*: **Implementing facility-based kangaroo mother care services: lessons from a multi-country study in Africa**. *BMC Health Serv Res* 2014, **14**:293.

3. Bhanbhro S, Gee M, Cook S, Marston L, Lean M, Killaspy H: **Recovery-based staff training intervention within mental health rehabilitation units: a two-stage analysis using realistic evaluation principles and framework approach**. *BMC Psychiatry* 2016, **16**:292.

4. Mazzocato PH, R. J.;Brommels, M.;Aronsson, H.;Backman, U.;Elg, M.;Thor, J.: **How does lean work in emergency care? A case study of a lean-inspired intervention at the Astrid Lindgren Children's hospital, Stockholm, Sweden**. *BMC health services research* 2012, **12**:28.

5. Gould DJ, Hale R, Waters E, Allen D: **Promoting health workers' ownership of infection prevention and control: using Normalization Process Theory as an interpretive framework**. *J Hosp Infect* 2016, **94**(4):373-380.

6. Jangland E, Gunningberg L: **Improving patient participation in a challenging context: a 2-year evaluation study of an implementation project**. *J Nurs Manag* 2017, **25**(4):266-275.

7. Mitchell SE, Weigel GM, Laurens V, Martin J, Jack BW: **Implementation and adaptation of the Re-Engineered Discharge (RED) in five California hospitals: a qualitative research study**. *BMC Health Serv Res* 2017, **17**(1):291.

8. Naldemirci O, Wolf A, Elam M, Lydahl D, Moore L, Britten N: **Deliberate and emergent strategies for implementing person-centred care: a qualitative interview study with researchers, professionals and patients**. *BMC Health Serv Res* 2017, **17**(1):527.

9. Robert G, Morrow E, Maben J, Griffiths P, Callard L: **The adoption, local implementation and assimilation into routine nursing practice of a national quality improvement programme: the Productive Ward in England**. *J Clin Nurs* 2011, **20**(7-8):1196-1207.

10. Fleiszer AR, Semenic SE, Ritchie JA, Richer MC, Denis JL: **An organizational perspective on the long-term sustainability of a nursing best practice guidelines program: a case study**. *BMC Health Serv Res* 2015, **15**:535.

11. Matthew-Maich N, Ploeg J, Dobbins M, Jack S: **Supporting the Uptake of Nursing Guidelines: what you really need to know to move nursing guidelines into practice**. *Worldviews Evid Based Nurs* 2013, **10**(2):104-115.

12. Bouamrane MM, Mair FS: **Implementation of an integrated preoperative care pathway and regional electronic clinical portal for preoperative assessment**. *BMC Med Inform Decis Mak* 2014, **14**:93.

13. Rotteau L, Webster F, Salkeld E, Hellings C, Guttmann A, Vermeulen MJ, Bell RS, Zwarenstein M, Rowe BH, Nigam A *et al*: **Ontario's emergency department process improvement program: the experience of implementation**. *Acad Emerg Med* 2015, **22**(6):720-729.
