# Supplementary material for: The barriers and facilitators influencing the sustainability of hospital-based interventions: a systematic review
Source: BMC Health Serv Res. 2020 Jun 28;20:588. doi: 10.1186/s12913-020-05434-9 (PMC7321537; doi:10.1186/s12913-020-05434-9)
Supplement: Supplementary file 12 — Additional file 12. Key examples of barriers and facilitators identified within the Resources theme. [file 12913_2020_5434_MOESM12_ESM.docx]

**ADDITIONAL FILE 12. DEFINITION AND KEY EXAMPLES OF BARRIERS AND FACILITATORS IDENTIFIED IN THE RESOURCES THEME**

| **Theme: Resources** | **CONSTRUCT** | **DEFINITION (AS DESCRIBED BY LENNOX ET AL. 2018)(1)** | **KEY EXAMPLE (BARRIERS)** | **KEY EXAMPLE (FACILITATORS)** |
| --- | --- | --- | --- | --- |
|  | General | Any resources needed to manage and maintain an initiative. | “Conversely, service user interviews revealed how resource issues had led to activities” (Bhanbhro 2016, p6) (2) | “Whether or not the activities went ahead as planned was mediated by the extent to which the proposed activity harmonised with the priorities of the wider hospital organisation and resources available to the ward team.” (Bridges 2017, p974) (3) |
|  | Funding | Having adequate funding for the initiative to be implemented, embedded and sustained. | “Despite the positive feedback on the measures collected by the OMSC, DMs felt that it is difficult to sustain programs that require data management without dedicated resources.” (Campbell 2011, p8) (4) | “the ‘gift’ of external funding for these positions” (Bernstein 2009, p1229) (5) |
|  | Infrastructure | The resources required to support the initiative to be delivered such as buildings, office space, materials and supplies. | “Supervision not sustained because of lack of transport” (Bergh, 2014, p6) (6) | “The visual management system (takt board) was used to identify and document flow problems during the day, to support continual improvement.” (Mazzocato 2012, p10) (7) |
|  | Staff | Having sufficient number of staff to meet the requirements of the initiative. | “Nursing staffing stability was the aspect of resources that most impacted routinization. All subcases struggled with the negative effects of staff turnover. [examples of retirement and historically problematic]” (Fleiszer 2016, p212) (8) | “providing sufficient resources and support, in particular allocated budgets for backfill of staff time” (Robert 2011, p1200) (9) |
|  | Time | Energy and time to dedicate to the initiative | “[micro level factors] most were 'struggling' to start cascading the training to their ward colleagues within the first month. This was because of staff shortages, clinical priorities, lack of time during a busy 12 h shift, and the availability of computers with internet access for the e-learning programmes” (Ilott 2016, p7) (10) | “However, we did find that hospitals with a SCC with some dedicated time (as little as 10%) to educate and train staff, promote the OMSC (either themselves or by enlisting champions), and ensure that patients are being identified, offered counselling, and follow-up had achieved OMSC activity rates that were higher than baseline. These actions may influence the sustainability of the program by enhancing the interactions between the health issues, stakeholders and program” (Campbell 2011, p8) (4) |

**REFERENCES**

1. Lennox L, Maher L, Reed J. Navigating the sustainability landscape: a systematic review of sustainability approaches in healthcare. Implement Sci. 2018;13(1):27.

2. Bhanbhro S, Gee M, Cook S, Marston L, Lean M, Killaspy H. Recovery-based staff training intervention within mental health rehabilitation units: a two-stage analysis using realistic evaluation principles and framework approach. BMC Psychiatry. 2016;16:292.

3. Bridges J, May C, Fuller A, Griffiths P, Wigley W, Gould L, et al. Optimising impact and sustainability: a qualitative process evaluation of a complex intervention targeted at compassionate care. BMJ Qual Saf. 2017;26(12):970-7.

4. Campbell S, Pieters K, Mullen KA, Reece R, Reid RD. Examining sustainability in a hospital setting: case of smoking cessation. Implement Sci. 2011;6:108.

5. Bernstein E, Topp D, Shaw E, Girard C, Pressman K, Woolcock E, et al. A preliminary report of knowledge translation: lessons from taking screening and brief intervention techniques from the research setting into regional systems of care. Acad Emerg Med. 2009;16(11):1225-33.

6. Bergh AM, Kerber K, Abwao S, de-Graft Johnson J, Aliganyira P, Davy K, et al. Implementing facility-based kangaroo mother care services: lessons from a multi-country study in Africa. BMC Health Serv Res. 2014;14:293.

7. Mazzocato PH, R. J.;Brommels, M.;Aronsson, H.;Backman, U.;Elg, M.;Thor, J. How does lean work in emergency care? A case study of a lean-inspired intervention at the Astrid Lindgren Children's hospital, Stockholm, Sweden. BMC health services research. 2012;12:28.

8. Fleiszer AR, Semenic SE, Ritchie JA, Richer MC, Denis JL. A unit-level perspective on the long-term sustainability of a nursing best practice guidelines program: An embedded multiple case study. Int J Nurs Stud. 2016;53:204-18.

9. Robert G, Morrow E, Maben J, Griffiths P, Callard L. The adoption, local implementation and assimilation into routine nursing practice of a national quality improvement programme: the Productive Ward in England. J Clin Nurs. 2011;20(7-8):1196-207.

10. Ilott I, Gerrish K, Eltringham SA, Taylor C, Pownall S. Exploring factors that influence the spread and sustainability of a dysphagia innovation: an instrumental case study. BMC Health Serv Res. 2016;16(1):406.
