# Supplementary material for: The barriers and facilitators influencing the sustainability of hospital-based interventions: a systematic review
Source: BMC Health Serv Res. 2020 Jun 28;20:588. doi: 10.1186/s12913-020-05434-9 (PMC7321537; doi:10.1186/s12913-020-05434-9)
Supplement: Supplementary file 13 — Additional file 13. Key examples of barriers and facilitators identified within the organisational setting theme. [file 12913_2020_5434_MOESM13_ESM.docx]

**ADDITIONAL FILE 13. DEFINITION AND KEY EXAMPLES OF BARRIERS AND FACILITATORS IDENTIFIED IN THE ORGANISATIONAL SETTING THEME**

| **THEME: ORGANISATIONAL SETTING** | **CONSTRUCT** | **DEFINITION (AS DESCRIBED BY LENNOX ET AL. 2018) (1)** | **KEY EXAMPLE (BARRIERS)** | **KEY EXAMPLE (FACILITATORS)** |
| --- | --- | --- | --- | --- |
|  | Integration with existing programs and policies | The need to ensure an initiative was embedded into organisational structures, programmes and policies. | “Lack of integrated planning of staff, operating rooms, and beds at institutional level was pointed out as impeding the sustainability of both programs.” (Ament 2017, p1139) (2) | “At an organisational level, aligning the anticipated benefits of the intervention with the needs and priorities of the organisation provided an opportunity to garner support for the initiative” (Green 2017, p5) (3) |
|  | Intervention adaptation and receptivity | The ability of an initiative to respond to change and adapt to fit with local contexts and requirements. | “There were no 'recipes' or simple guidelines on how to sustain audit” (Belizan 2011, p7) (4) | “Clinicians were involved in modifying and adapting the interventions to the context. Their new understanding of the clinical system influenced how this was done. With the new understanding, effects of the interventions were evaluated and adapted according to how the entire clinical pathway was thought should work.” (Hovlid 2012, p9) (5) |
|  | Opposition | Resistance from stakeholders to the initiative due to other priorities or competing interests. | Fatigue from previous implementations (Naldermirci 2017, p4) (6) | NONE |
|  | Organisational readiness and capacity | Capacity and readiness of the organisation to undertake the initiative. | “Challenges related to outdated, conflicting, and confusing information across multiple sites” (Gramlich 2017, p8) (7) | "Succession planning" was also mentioned as a prerequisite for sustainability” (Belizan 2011, p7) (4) |
|  | Organisational values and culture | Organisational beliefs and values that support a culture for the initiative and its sustainability. | “An important finding was that the nurse managers reported a change in attitude among staff, who accepted that patients should be involved in their own care. However, change was not embedded in the whole health care team and all interactions and relationships with patients, so traditional ward rounds with the surgeon continued to be performed without the involvement of the patient in important decisions.” (Jangland 2017, p273-4) (8) | “Culture was perceived to differ between wards. Surgery was perceived as a discipline that is relatively more open to trying out innovations than other disciplines. [Supporting quote included.]” (Ament et al., 2017, p1140) (2) |
|  | Support available | Support in the form of reminders, staff, technical and educations, available to enhance delivery and maintenance of the initiative. | “Our findings indicate that higher and more sustained impact for interventions such as CLECC may only be possible through more substantial restructuring that reshapes the conditions in which people are able to act…we have identified a number of concrete but modifiable barriers that merit attention in such design, including lack of time and institutional rules that undermine the value of staff-to-staff social support. They also include more clearly defining the role of nursing managers in signalling the legitimacy of staff providing each other with emotional support, supporting nursing teams to meet and learn together.” (Bridges 2017, p976) (9) | “In all three units, individual and group supervision was set as the norm and was regularly maintained and appeared to contribute to a supportive organisation”. (Bhanbhro 2016, p10) (10) |

**REFERENCES**

1. Lennox L, Maher L, Reed J. Navigating the sustainability landscape: a systematic review of sustainability approaches in healthcare. Implement Sci. 2018;13(1):27.

2. Ament SMC, Gillissen F, Moser A, Maessen JMC, Dirksen CD, von Meyenfeldt MF, et al. Factors associated with sustainability of 2 quality improvement programs after achieving early implementation success. A qualitative case study. J Eval Clin Pract. 2017;23(6):1135-43.

3. Green SA, Bell D, Mays N. Identification of factors that support successful implementation of care bundles in the acute medical setting: a qualitative study. BMC Health Serv Res. 2017;17(1):120.

4. Belizan M, Bergh AM, Cilliers C, Pattinson RC, Voce A, Synergy G. Stages of change: A qualitative study on the implementation of a perinatal audit programme in South Africa. BMC Health Serv Res. 2011;11:243.

5. Hovlid EB, O.;Haug, K.;Aslaksen, A. B.;von Plessen, C. Sustainability of healthcare improvement: what can we learn from learning theory? BMC health services research. 2012;12:235.

6. Naldemirci O, Wolf A, Elam M, Lydahl D, Moore L, Britten N. Deliberate and emergent strategies for implementing person-centred care: a qualitative interview study with researchers, professionals and patients. BMC Health Serv Res. 2017;17(1):527.

7. Gramlich LM, Sheppard CE, Wasylak T, Gilmour LE, Ljungqvist O, Basualdo-Hammond C, et al. Implementation of Enhanced Recovery After Surgery: a strategy to transform surgical care across a health system. Implement Sci. 2017;12(1):67.

8. Jangland E, Gunningberg L. Improving patient participation in a challenging context: a 2-year evaluation study of an implementation project. J Nurs Manag. 2017;25(4):266-75.

9. Bridges J, May C, Fuller A, Griffiths P, Wigley W, Gould L, et al. Optimising impact and sustainability: a qualitative process evaluation of a complex intervention targeted at compassionate care. BMJ Qual Saf. 2017;26(12):970-7.

10. Bhanbhro S, Gee M, Cook S, Marston L, Lean M, Killaspy H. Recovery-based staff training intervention within mental health rehabilitation units: a two-stage analysis using realistic evaluation principles and framework approach. BMC Psychiatry. 2016;16:292.
