# Supplementary material for: The barriers and facilitators influencing the sustainability of hospital-based interventions: a systematic review
Source: BMC Health Serv Res. 2020 Jun 28;20:588. doi: 10.1186/s12913-020-05434-9 (PMC7321537; doi:10.1186/s12913-020-05434-9)
Supplement: Supplementary file 14 — Additional file 14. Key examples of barriers and facilitators identified within the external environment theme. [file 12913_2020_5434_MOESM14_ESM.docx]

**ADDITIONAL FILE 14. DEFINITION AND KEY EXAMPLES OF BARRIERS AND FACILITATORS IDENTIFIED IN THE EXTERNAL ENVIRONMENT THEME**

| **Theme: External environment** | **CONSTRUCT** | **DEFINITION (AS DESCRIBED BY LENNOX ET AL. 2018)(1)** | **KEY EXAMPLE (BARRIERS)** | **KEY EXAMPLE (FACILITATORS)** |
| --- | --- | --- | --- | --- |
|  | **Awareness and raising the profile** | Ensuring that stakeholder such as the community are aware of the initiative and its benefits and strategic steps are taken to raise the profile of the project to garner further support though media, marketing and publications. | Less intensive communication between hospitals regarding care delivery in the postimplementation phase was mentioned in the ERAS case and was perceived as a barrier to sustainability by several respondents. [Supporting quote provided.] (Ament, 2017, p1140) (2) | Sustainability was facilitated by intensive communication and a strong network connecting different centers. Intensive relations with other centers meant that respondents felt able to share their experiences with the program. [Supporting quote provided.]...external networking with direct peers in other hospitals and sharing experiences with colon cancer and breast cancer teams (Ament, 2017, p1140) (2) |
|  | **Socioeconomic and political considerations** | Awareness of the potential impact of outside forces in the environment, government or society that may impact initiative funding, processes or priorities | RNs, DNs and HCOs tried to make the DPP work smoothly for the patients and their relatives but felt that society was not in step with the healthcare system. They stated that increasing elderly population, a non-captive market, the decreasing number of hospital beds, shorter hospital stays, the lower number of sheltered homes and limited personnel resources led to higher demands and a greater workload for all staff working with the DP and impeded the process. (Nordmark, 2016, p7)(3) | Benefit: Policy brings attention to a greater need for infection prevention, allowing necessary resources to be allocated. (McClung, 2017, p1066) (4) |
|  | **Spread to other organisations** | Ability of an innovation to show benefits which are either spread within an organisation or to other organisations. | NONE | Even stronger than external networking with direct peers in other hospitals and sharing experiences with colon cancer and breast cancer teams is spreading the experiences to professionals in other fields. This spread was perceived as an important factor related to sustainability as it was perceived as further confirmation of their effectiveness, and it increased the intention to sustain the care in accordance with these programs. [Examples given] (Ament, 2017, p1140) (2) |
|  | **Urgency** | The urgency or motivation to maintain an initiative based on its potential to support an important and relevant healthcare need. | NONE | Two prominent external contextual factors were 1) the focus of federal penalties on specific patient diagnostic populations (heart failure, pneumonia and heart attack) and 2) the increased availability of grant funding and technical assistance resources to incentivize US hospitals to improve care transitions (Mitchell et al, 2017, p7) (5) |

**REFERENCES**

1. Lennox L, Maher L, Reed J. Navigating the sustainability landscape: a systematic review of sustainability approaches in healthcare. Implement Sci. 2018;13(1):27.

2. Ament SMC, Gillissen F, Moser A, Maessen JMC, Dirksen CD, von Meyenfeldt MF, et al. Factors associated with sustainability of 2 quality improvement programs after achieving early implementation success. A qualitative case study. J Eval Clin Pract. 2017;23(6):1135-43.

3. Nordmark S, Zingmark K, Lindberg I. Process evaluation of discharge planning implementation in healthcare using normalization process theory. BMC Med Inform Decis Mak. 2016;16:48.

4. McClung L. Health care worker perspectives of their motivation to reduce hospital-acquired infections. Journal of Investigative Medicine. 2017;65(4):824.

5. Mitchell SE, Weigel GM, Laurens V, Martin J, Jack BW. Implementation and adaptation of the Re-Engineered Discharge (RED) in five California hospitals: a qualitative research study. BMC Health Serv Res. 2017;17(1):291.
